# Synthesis of Coumarin Derivatives: A New Class of Coumarin-Based G Protein-Coupled Receptor Activators and Inhibitors

**DOI:** 10.3390/polym14102021

**Published:** 2022-05-15

**Authors:** Zhe Fu, Linjie Zhang, Sijin Hang, Shiyi Wang, Na Li, Xiaojing Sun, Zian Wang, Ruilong Sheng, Fang Wang, Wenhui Wu, Ruihua Guo

**Affiliations:** 1 College of Food Science and Technology, Shanghai Ocean University, Shanghai 201306, China; fzshou2019@163.com (Z.F.); ljzhang20010428@163.com (L.Z.); sjhang2022@163.com (S.H.); ln16658@163.com (N.L.); sunxj1997@163.com (X.S.); wzamengnan1999@163.com (Z.W.); whwu@shou.edu.cn (W.W.); 2 AIEN Institute, Shanghai Ocean University, Shanghai 201306, China; wjcxx3111@163.com; 3 CQM—Centro de Química da Madeira, Campus da Penteada, Universidade da Madeira, 9000-390 Funchal, Portugal; ruilong.sheng@staff.uma.pt; 4 School Basic Medicine, Shanghai University of Medicine and Health Science, Shanghai 201318, China; wangf_18@sumhs.edu.cn; 5 Laboratory of Quality and Safety Risk Assessment for Aquatic Product on Storage and Preservation, Ministry of Agriculture, Shanghai 201306, China; 6 Engineering Research Center of Aquatic-Product Processing & Preservation, Shanghai 201306, China

**Keywords:** coumarin, G protein-coupled receptors, synthesis, structure–activity relationships

## Abstract

To expand the range of daphnetin-based inhibitors/activators used for targeting G protein-coupled receptors (GPCRs) in disease treatment, twenty-five coumarin derivatives 1–25, including 7,8-dihydroxycoumarin and 7-hydroxycoumarin derivatives with various substitution patterns/groups at C3-/4- positions, were synthesized via mild Pechmann condensation and hydroxyl modification. The structures were characterized by ^1^H NMR, ^13^C NMR and ESI-MS. Their inhibition or activation activities relative to GPCRs were evaluated by double-antibody sandwich ELISA (DAS–ELISA) in vitro. The results showed that most of the coumarin derivatives possessed a moderate GPCR activation or inhibitory potency. Among them, derivatives 14, 17, 18, and 21 showed a remarkable GPCR activation potency, with EC_50_ values of 0.03, 0.03, 0.03, and 0.02 nM, respectively. Meanwhile, derivatives 4, 7, and 23 had significant GPCR inhibitory potencies against GPCRs with IC_50_ values of 0.15, 0.02, and 0.76 nM, respectively. Notably, the acylation of hydroxyl groups at the C-7 and C-8 positions of 7,8-dihydroxycoumarin skeleton or the etherification of the hydroxyl group at the C-7 position of the 7-hydroxycoumarin skeleton could successfully change GPCRs activators into inhibitors. This work demonstrated a simple and efficient approach to developing coumarin derivatives as remarkable GPCRs activators and inhibitors via molecular diversity-based synthesis.

## 1. Introduction

**Figure 1:**
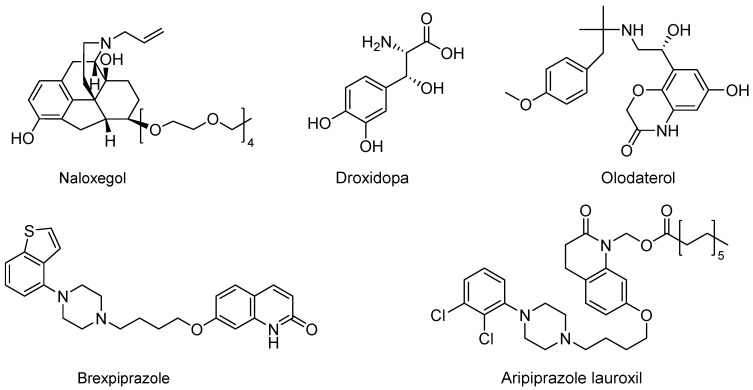
Chemical structures of drugs targeting GPCRs approved by the FDA.

G protein-coupled receptors (GPCRs) are a large family of membrane-protein receptors with a transmembrane α-helix structure which are involved in a variety of physiological functions and have a variety of complex and key signaling pathways for bioactivities [1,2]. The most used classification of GPCRs divides them into six categories: rhodopsin-like receptors (class A), secretin-like receptors (class B), metabotropic glutamate receptors (class C), pheromone receptors (class D), cAMP receptors (class E), and frizzled/smoothened family receptors (class F). Among them, class A is the most studied, and the structures of these receptors have also been identified. However, few studies on the structures of protein receptors have been reported for class B-F. In 2013, the first human glucagon-receptor structure of class B GPCRs was confirmed [3]. The GPCRs’ relevant activities are regulated by two signaling pathways: cAMP signaling and phosphatidylinositol signaling [4]. More than 30% of the drugs approved by the FDA are able to target GPCRs [5]. For instance (Figure 1), Naloxegol is used for opioid-induced constipation (a μ-opioid receptor antagonist) [6], Droxidopa is used for symptomatic postural hypotension [7], Aripiprazole Lauroxil is used for schizophrenia (a dopamine D2 receptor, and a partial agonist of the 5-HT_1A_ receptor and the 5-HT_2A_ receptor antagonist) [8], Brexpiprazole is used for depression (with mixed opioid receptor activity) [9], and Olodaterol is used for chronic obstructive pulmonary disease (a β 2-adrenergic receptor agonist) [10]. Many GPCRs-targeting drugs still have complicated chemical structures or tedious synthesis routes, which increase their costs in large-scale production. Therefore, it is necessary to develop simple-structured, easy-to-synthesize, and new GPCR-targeting drugs/compounds for disease treatment.

**Figure 2:**
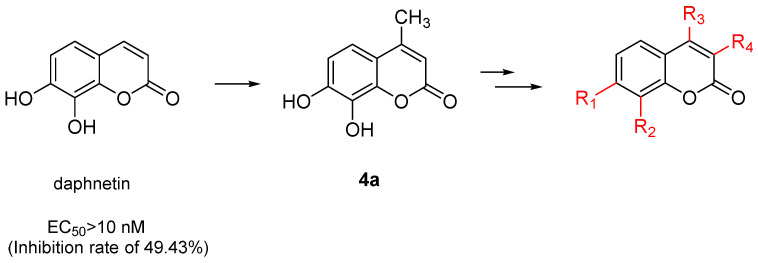
Chemical structures of daphnetin and its derivatives for the development of GPCR inhibitors/activators.

Natural products with various skeletons and diverse bioactivities are considered important sources for drug discovery [11,12,13,14,15,16,17,18,19,20,21,22,23,24,25]. Since the discovery of natural coumarin in 1820 [26], numerous coumarin derivatives have been extracted from the secondary metabolites of many plant species [27]. Coumarin derivatives exhibit diverse bioactivities, such as anti-bacterial [28,29], anticoagulant [30], anti-inflammatory [31,32], anti-tumor [33,34,35], and antioxidant effects [36], etc. [37,38,39,40,41,42]. Thus, they have received a large amount of attention from synthetic chemists. Up until now, some synthetic methodologies, including the Pechmann condensation [43], the Perkin reaction [44], Knoevenagel condensation [45], metal-catalyzed cyclization [46], the Wittig reaction [47], and other reactions [48,49], have been developed to prepare coumarin derivatives. Previously, we reported that daphnetin (a naturally occurring coumarin) derivatives (Figure 2) with various substitution patterns/groups exhibited activities from inhibitory potency to activation potency in GPCRs. The optimized compound 4a and its derivatives showed the highest inhibitory or activation potency in GPCRs [50,51].

In order to further improve the activities of coumarin derivatives by expanding their molecular diversity, 7, 8-dihydroxycoumarin and 7-hydroxycoumarin derivatives with various substitution patterns/groups at the C-3/-4 positions were synthesized and characterized in this study. Their pharmaceutical potency in GPCRs were evaluated by a double-antibody sandwich ELISA (DAS–ELISA) in vitro. The synthesized compounds were used as an antigen in the kit. The enzyme substrate and the specific antibody in the enzyme plate formed a ‘sandwich’. Finally, the substrate of the enzyme was added, and the content of antigen was determined based on the absorbance of the enzymolysis product. Detailed steps are shown in “2.4 DAS-ELISA evaluation of coumarin derivatives on GPCRs”. It was disclosed that derivatives 14, 17, 18, and 21 displayed a remarkable activation potency in GPCRs with EC_50_ values of 0.03, 0.03, 0.03, and 0.02 nM, respectively. On the other hand, derivatives 4, 7, and 23 possessed significant inhibitory potency on GPCRs with IC_50_ values of 0.15, 0.02, and 0.76 nM, respectively.

## 2. Materials and Methods

### 2.1. Materials and Methods

All chemicals and reagents used in the study were commercially available and used as received. Thin-layer chromatography (TLC) and column chromatography (300–400 mesh) were achieved using instruments from Qingdao Makall Group Co., Ltd. (Qingdao, China). ^1^H NMR and ^13^C NMR spectra were recorded on a Bruker DRX-500 (^1^H/^13^C, 500 MHz/125 MHz) spectrometer (Bruker, Bremerhaven, Germany) or a JEOL 400YH (^1^H/^13^C, 400 MHz/100 MHz) and chemical shifts were given in δ with the TMS as an internal reference. The ESI-MS spectroscopy was measured on an Advantage Max LCQ Thermo-Finnigan mass spectrometer. The ELISA kit was purchased from Shanghai Fusheng Industry Co., Ltd. (Shanghai, China), and the standard in the kit was Class B GPCRs. 

### 2.2. General Procedure for Preparation the Derivatives 1–25

#### 2.2.1. General Procedure for Preparation of Derivatives 1–6

Pyrogallol (1.0 equiv.) and scandium (III) trifluoromethanesulfonate (Sc(OTf)_3_) (0.1 equiv.) were added to a solution of appropriately different β-ketoesters (1.0 equiv.) at room temperature. The reaction mixture was stirred at 85 ℃ until the starting material disappeared on the TLC. The reaction mixture was quenched with 50 mL of water and extracted with CH_2_Cl_2_ (3 × 50 mL). The combined organic layer was washed three times with 50 mL of brine, dried over anhydrous Na_2_SO_4_, concentrated under reduced pressure, and then purified by a column chromatography on the silica gel to obtain derivatives 1–6 [52,53,54]. Finally, the molecular structures of the synthesized derivatives were fully characterized in the Supplementary Materials (Figures S1–S18).

#### 2.2.2. General Procedure for Preparation of Derivatives 7 and 8

To a solution of derivative 6 (1 equiv.) in pyridine (1.0 mL), DMAP (0.5 equiv.) and Acetic anhydride or propionic anhydride (4.0 equiv.) were added in and stirred at room temperature until the starting material disappeared on the TLC. Then, the reaction mixture was diluted with 50 mL of CH_2_Cl_2_ and washed three times with 50 mL of 5% HCl and saturated NaHCO_3_ (3 × 30 mL). After that, the organic layer was dried over anhydrous Na_2_SO_4_ and then concentrated under reduced pressure. The residue was purified by a column chromatography on the silica gel to access derivatives 7 [55] and 8. Finally, the molecular structures of the synthesized derivatives were fully characterized in the Supplementary Materials (Figures S19–S24).

#### 2.2.3. General Procedure for Preparation of Derivatives 9–20

Resorcinol (1.2 equiv.) and scandium (III) trifluoromethanesulfonate (Sc(OTf)3) (0.1 equiv.) were added to a solution of appropriately different β-ketoesters (1.0 equiv.) at room temperature. The reaction mixture was stirred at 85 ℃ until the starting material disappeared on the TLC. The reaction mixture was quenched with 50 mL of water and extracted with CH_2_Cl_2_ (3 × 50 mL). The combined organic layer was washed three times with 50 mL of brine, dried over anhydrous Na_2_SO_4_, concentrated under reduced pressure, and then purified by a column chromatography on the silica gel to obtain derivatives 9–20 [52,56,57,58,59]. Finally, the molecular structures of the synthesized derivatives were fully characterized in the Supplementary Materials (Figures S25–S57).

#### 2.2.4. General Procedure for Preparation of Derivatives 21

Derivative 9 (100 mg, 1.0 equiv.) was dissolved in a solution of sodium hydroxide (16.4 mg, 10 equiv.) in water (0.41 mL) and the mixture was maintained at an ice bath temperature. A solution of 2, 4-dichloroprymidine (122.1 mg, 2.0 equiv.) in acetone (1.6 mL) was added dropwise, and the mixture was stirred and refluxed until the starting material disappeared on the TLC [60]. The reaction mixture was quenched with 50 mL of water and extracted with CH_2_Cl_2_ (3 × 50 mL). The combined organic layer was washed three times with 50 mL of brine, dried over anhydrous Na_2_SO_4_, concentrated under reduced pressure, then purified by a column chromatography on the silica gel to obtain derivatives 21. Finally, the molecular structures of the synthesized derivatives were fully characterized in the Supplementary Materials (Figures S58–S61).

#### 2.2.5. General Procedure for Preparation of Derivatives 22 and 23

To a solution of derivatives 17 (1 equiv.) in acetone (2.0 mL), K_2_CO_3_ (10 equiv.) was added at room temperature. After half an hour, iodide methane or bromoethane (3.0 equiv.) was added and the resulting mixture was stirred at 65 ℃ until the starting material disappeared from the TLC. The residue was added with 50 mL water, and the following mixture solution was extracted with CH_2_Cl_2_ (3 × 50 mL). The combined organic layers were washed with brine, and then dried over anhydrous Na_2_SO_4_. After the removal of the solvent under reduced pressure, the crude products were produced; these were purified by column chromatography on silica gel to achieve derivatives 22 [61] and 23. Finally, the molecular structures of the synthesized derivatives were fully characterized in the Supplementary Materials (Figures S61–S66).

#### 2.2.6. General Procedure for Preparation of Derivatives 24 and 25

A solution of derivative 7 (1 equiv.), DMAP (0.5 equiv.) and acetic anhydride or propionic anhydride (4.0 equiv.) in pyridine (1.0 mL) was stirred at room temperature until the starting material disappeared on the TLC. Then the reaction mixture was diluted with 50 mL of CH_2_Cl_2_ and washed three times with 50 mL of 5% HCl and saturated NaHCO_3_ (3 ×30 mL). Following, the organic layer was dried over anhydrous Na_2_SO_4_ and then concentrated under reduced pressure. The residue was purified by a column chromatography on the silica gel to access derivatives 24 and 25. Finally, the molecular structures of the synthesized derivatives were fully characterized in the Supplementary Materials (Figures S67–S72).

### 2.3. Sample Pretreatment

All the synthesized coumarin derivatives were dissolved in 500 μL of DMSO, and then diluted to 1 mL by distilled water to get 1 mg/mL of stock solution. Finally, the stock solution was diluted to 1000 μg/L, 10 μg/L, 0.1 μg/L, and 0.001 μg/L in turn and stored in a refrigerator at 4 ℃ for reserve.

### 2.4. DAS-ELISA Evaluation of Coumarin Derivatives on GPCRs

The inhibitory/activation activities of all of the synthesized coumarin derivatives on GPCRs were evaluated through incubation using mouse GPCRs provided by the ELISA kit (Shanghai Fusheng Industrial Co., Ltd. Shanghai, China). Other components of the kit included a water solution (30 times concentrated), a HRP conjunction reagent, a sample diluent, a standard diluent, standard, a chromogenic reagent A, a chromogenic reagent B, and a stop solution.

The 15 μL of stock solution and 15 μL of diluted standard solution were thoroughly mixed and incubated at 37 ℃ for 10 mins to prepare a reaction solution. The assay was conducted according to the kit protocol, that is, by adding the 25 μL of reaction solution to a 96-well enzyme-labeled plate containing solid phase anti-GPCRs antibodies. Then the plate was incubated for 30 mins at 37 ℃. Then, 25 μL of Chromogener A and 25 μL of Chromogener B were then added to react. Finally, 25 μL of the termination solution was added to each well to terminate the reaction and the mixture was fully mixed. Derivatives were tested in four different concentrations: 10 ng/L, 1 ng/L, 0.1 μg/mL, and 10 μg/L. A diluent was prepared, dissolved and diluted with the other components of the kit according to the instructions in the kit, and a termination solution was added to the reaction. Then, the OD value was obtained by a microplate reader in 15 mins. All the derivatives were set into two independent experimental groups, and the inhibition of GCPRs, which was expressed as a percentage, was based on these groups [50,62,63,64].

## 3. Results and Discussion

### 3.1. Results

#### Chemistry

**Scheme 1:**
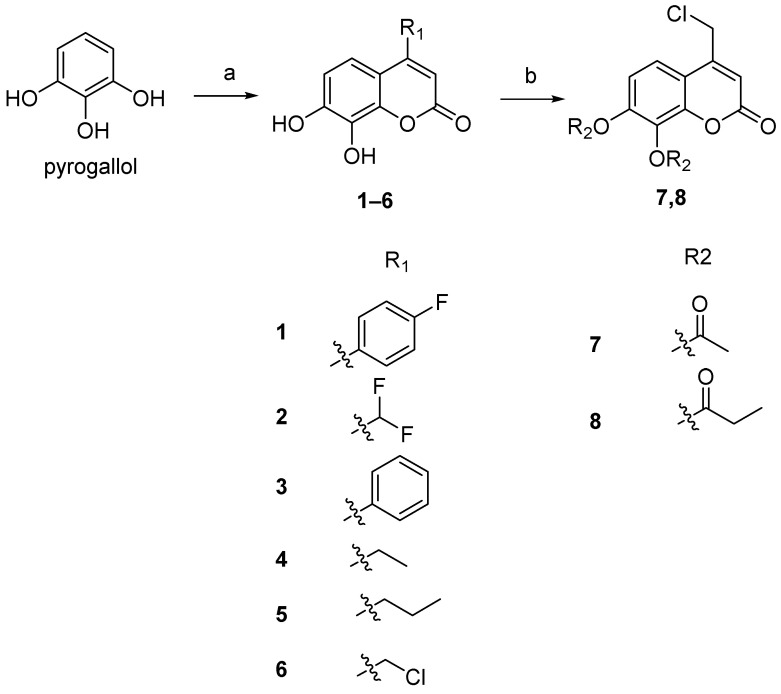
Reagents and conditions: (**a**) Sc(OTf)_3_, β-ketoesters, 80 ℃, 0.5–2 h; (**b**) acetic anhydride or propionic anhydride, pyridine, DMAP, r.t., 2–3 h.

7,8-dihydroxycoumarin derivatives (1–8) and 7-hydroxycoumarin derivatives (9–25) with various substitution patterns/groups at C3-/4- positions were synthesized via Pechmann condensation. First, 7,8-dihydroxycoumarin derivatives 1–6 with different substituents at the C-4 position were synthesized, by a react pyrogallol with different β-ketoesters in the presence of Scandium(III) triflate (Sc(OTf)_3_) as the catalyst, through a one-pot synthesis (Scheme 1) [65]. Derivative 6 reacted with acetic anhydride and propionic anhydride in the presence of 4-dimethylaminopyridine (DMAP) as the catalyst in pyridine to produce the derivatives 7 and 8, respectively. 

**Scheme 2:**
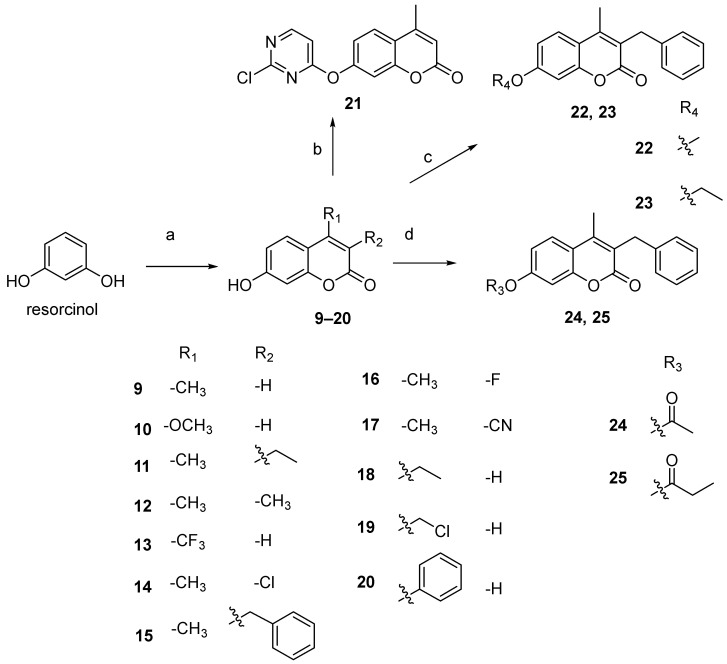
Reagents and conditions: (**a**) SC(OTf)_3_, β-ketoesters, 80 ℃, 0.5–2 h; (**b**) 2,4–dichloropyrimidine, acetone, NaOH, reflux 5 h; (**c**) CH_3_I or CH_2_CH_3_Br, acetone, K_2_CO_3_, reflux 2–3 h; (**d**) acetic anhydride or propionic anhydride, pyridine, DMAP, r.t., 2–3 h.

Similarly, resorcinol as the starting material, which reacts with different β-ketoesters in the presence of Scandium(III) triflate (Sc(OTf)_3_), was used to produce 7-hydroxycoumarin derivatives 9–25 with different substitution groups in C3-/C4- positions through a one-pot synthesis (Scheme 2). Then, derivative 9 reacted with 2,4-dichloropyrimidine in the presence of K_2_CO_3_ in acetone to afford derivatives 22 and 23. Derivative 15 reacted with acetic anhydride and propionic anhydride in the presence of DMAP to produce derivatives 24 and 25, respectively. Derivative 15 was treated with various CH_3_I or C_2_H_5_Br in the presence of K_2_CO_3_ in acetone or *N,N*-dimethylacetamide to achieve derivatives 22 and 23. All of the synthesized compounds were fully characterized by NMR and ESI-MS spectroscopy. All of the chemical shifts were expressed in parts per million (ppm) relative to internal tetramethylsilane (TMS), and coupling constants (J) were expressed in hertz (Hz). MS dates have been listed in the Supplementary Materials. 

### 3.2. Discussion

#### Biological Evaluation of The Synthesized Coumarin Derivatives at GPCRs in Vitro

The as-synthesized twenty-five coumarin derivatives (1–25) were evaluated to determine their activities in GPCRs using the DAS-ELISA method [50,62]. The results showed that coumarin derivatives possessed different bioactivities in GPCRs ranging from inhibition to activation (Figure S73–S75). The GPCRs’ inhibition/activation activity of each compound was expressed as the concentration of the compound that achieved a 50% inhibition (IC_50_)/50% activation (EC_50_) effect of the GPCRs’ standard at 10 ng/L. The results are shown in Table 1. Derivatives 1–25 had different activities on GPCRs, from inhibition to activation. It can be seen that 7,8-dihydroxycoumarin derivatives 1 and 6 showed an activation potency on GPCRs with EC_50_ values of 0.56 and 0.49 nM, respectively, while derivatives 4, 5, 7, and 8 showed an inhibitory potency on GPCRs; among them, 4 and 7 exhibited a significant inhibitory potency, with IC_50_ values of 0.15 nM and 0.02, respectively. For 7-hydroxycoumarin derivatives 9, 11, 12, 14, 16–19, and 21 with different substitution patterns/groups at C-3 or C-4 positions possessed an activation potency on GPCRs with EC_50_ values in the range of 0.02–7.75 nM, while 13 and 15 had an inhibitory potency on GPCRs with IC_50_ values of 7.75 and 3.60 nM.

Daphnetin had a weak inhibitory activity in GPCRs with an inhibition rate of 49.43% at 10 ng/L. Previous work has demonstrated that the replacement of the methyl group at the C-4 position of daphnetin (4a) was beneficial for the activation potency on GPCRs [50]. Thus, it was expected that the introduction of 4-fluorophenyl, difluoromethyl, phenyl, ethyl, *n*-propyl, and chloromethyl groups to C-4 positions in daphnetin to derivatives 1–6 may lead to different activation activities in GPCRs. The result showed that 4-fluorophenyl derivatives 1 possessed a higher GPCR activation potency (EC_50_ = 0.56 nM) than that of derivative 4a (EC_50_ = 2.65 nM). However, the difluoromethyl derivative 2 and the phenyl derivative 3 had very low GPCRs-activation potencies, with EC_50_ > 39.37 nM and EC_50_ > 43.83 nM, respectively. Compared to derivative 4a, derivatives 4 and 5 with an alkylation of the hydroxyl group at C-4 positions completely lost their GPCRs-activation potency but retained their GPCRs-inhibition potency. Moreover, a decreasing inhibitory potency from 4 (IC_50_ = 0.15 nM) to 5 (IC_50_ = 3.77 nM) was observed with a lengthened side-chain. The C-4-chloromethyl derivative 6 showed a better activation potency on GPCRs with an EC_50_ of 0.49 nM than derivative 4a, indicating that the chloride substitution group at the C-4 methyl position possessed a remarkable GPCR activation potency. To further enhance the activation potency, derivatives 7 and 8 were designed and synthesized by introducing acetyl and propinyl groups at the C-7 and C-8 positions of derivative 6, and their activities in GPCRs were tested. Interestingly, derivatives 7 and 8 had no GPCR activation potency. Acetylated derivative 7 showed a remarkable inhibitory activity, with an IC_50_ of 0.02 nM. Meanwhile, the propionylated derivative 8 showed a moderate inhibitory activity, with an IC_50_ of 6.86 nM. It can be disclosed that the acylation of hydroxyl groups at the C-7 and C-8 positions can successfully change GPCR activators into inhibitors.

To further investigate the structure–activity relationships (SARs), 7-hydroxycoumarin derivatives 9–25 with different substitution patterns/groups in C-4 were obtained by the reaction of resorcinol with different β-ketoesters. Derivatives 9, 18, and 19 with a methyl, ethyl, and chloromethyl group at the C-4 position had no GPCRs-inhibitory activity and showed a GPCRs- activation potency with EC_50_ values of 1.25, 0.03, and 4.22 nM, respectively. Derivatives 10 and 13 possessed relatively weak inhibitory activities on GPCRs with IC_50_ values of >48.84 and 7.75 nM, respectively. Furthermore, derivatives with methyl at the C-4 position were substituted by the different groups, C_2_H_5_, CH_3_, Cl, benzyl, F, and CN at the C-3 position to produce derivatives 11, 12, and 14–17. Derivatives 11, 12, 14, 16–17 showed a high GPCRs-activation potency with EC_50_ values in the range of 0.03–1.28 nM. However, derivative 15 with benzyl at the C-3 position possessed inhibitory activities against GPCRs from activation to inhibition with an EC_50_ value of 3.60 nM, in comparison to derivative 9 (EC_50_ = 1.25 nM) with CH_3_ at C-3, suggesting that the benzyl group at the C-3 position was not suitable for activation potency among the investigated derivatives. Derivative 11 with C_2_H_5_ at C-3 (EC_50_ = 1.28 nM) and derivative 12 with CH_3_ at C-3 (EC_50_ = 1.07 nM) retained their activation potencies on GPCRs compared to 9. The introduction of electron-withdrawing (Cl-, F- and CN-) groups to the C-3 position of derivative 9 produced derivative 14 (EC_50_ = 0.03 nM), 16 (EC_50_ = 0.24 nM), and 17 (EC_50_ = 0.03 nM), which seemed to increase the activation activity compared to derivative 9. These results indicated that electron-withdrawing groups at the C-3 position including Cl-, F- and CN- groups were more suitable for enhancing GPCRs-activation activities among the investigated derivatives.

A 2,4-dichloroprymidine reaction with derivative 9 (IC_50_ = 1.25 nM) produced derivative 21, which enhanced the inhibitory potency on GPCRs with IC_50_ of 0.02 nM. Derivatives 22 and 23, obtained through the etherification of the hydroxyl group at the C-7 position in 15 (GPCR activator, EC_50_ = 3.60 nM), presented an inhibitory potency on GPCRs with IC_50_ of 9.67 and 0.76 nM, respectively. This result suggests that the etherification of the hydroxyl group can successfully change GPCRs activators into inhibitors, which is identical with our previous reports [50]. The ethylated derivative 23 displayed better inhibitory activity on GPCRs with an IC_50_ of 0.76 nM, than the methylated derivative 22 (IC_50_ = 9.76 nM), meaning that lengthening the side-chain at the C-7 position of derivative 15 was not beneficial for inhibitory activity. Derivatives 24 (EC_50_ > 35.67 nM) and 25 (EC_50_ = 11.98 nM) with the acylation of the hydroxyl group at the C-7 positions of derivative 15 (EC_50_ = 3.60 nM) showed a decreasing activation potency, suggesting that the acylation of the hydroxyl group at C-7 was not beneficial for activation activity.

Inhibition rate = [OD(positive) − OD(test compound)]/[OD(positive) − OD(blank)] ∗ 100%.

IC_50_/EC_50_ were obtained from Modified Karber formula [66] as the following:

$$lg\rho =\mathrm{Xm}-I\left[P-\frac{\left(3-Pm-Pn\right)}{4}\right]$$

*ρ*: Concentration of compounds; Xm: Maximum dose; *I* = (Minimum inhibition rate)/Adjacent dose.

*P*: Sum of inhibition rates; *Pm*: Maximum inhibition rate; *Pn*: Minimum inhibition rate.

All values are the mean of two independent experiments ± SD.

## 4. Conclusions

**Figure 3:**
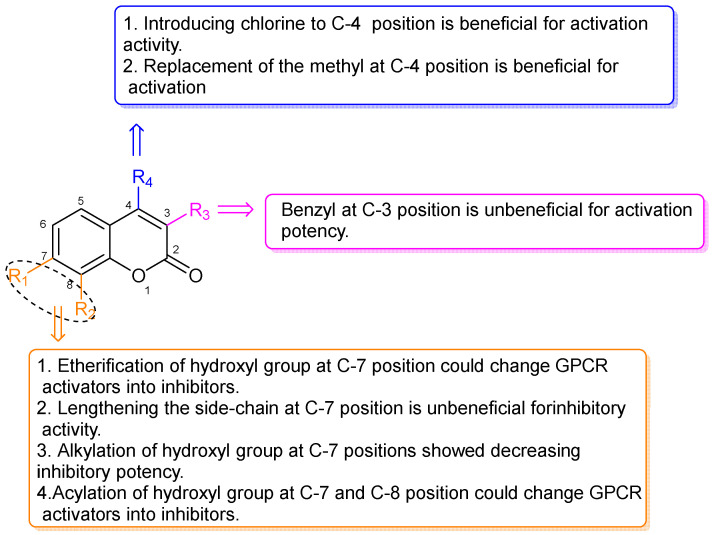
The SARs of coumarin derivatives in GPCR bioactivity.

In this work, twenty-five coumarin derivatives were synthesized via pyrogallol or resorcinol with different β-ketoesters. All synthesized derivatives were characterized by NMR and MS spectroscopies, and their bioactivities in GPCRs were evaluated in vitro using the DAS-ELISA method. Most derivatives possessed a moderate activation or inhibitory potency in GPCRs. Among them, derivatives 1, 6, 9, 11, 12, 14, 16–18, and 21 showed a remarkable GPCR activation potency, with EC_50_ values in the range of 0.03–1.28 nM. Derivatives 4, 7, and 23 had significant GPCRs inhibitory potencies with IC_50_ values of 0.15, 0.02, and 0.76 nM, respectively. SARs of coumarin derivatives from inhibitors to activators on GPCRs are shown in Figure 3 on the basis of the data (Table 1). Notably, the acylation of hydroxyl groups at the C-7 and C-8 positions of the 7,8-dihydroxycoumarin skeleton, or the etherification of the hydroxyl group at the C-7 position of the 7-hydroxycoumarin skeleton, can successfully change GPCR activators into inhibitors. This work demonstrates a simple and efficient approach to develop coumarin derivatives into remarkable GPCRs activators and inhibitors via molecular diversity-based synthesis.

## Figures and Tables

**Table 1:** In vitro GPCR inhibition IC_50_ or activation EC_50_ (nM) activities of daphnetin and derivatives 1–25.

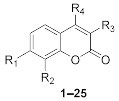						
No.	R_1_	R_2_	R_3_	R_4_	EC_50_(nM) (% Activation)	IC_50_(nM) (% INHIBITION)
Daphnetin	–OH	–OH	–H	–H		>10 (49.43%)
1	–OH	–OH	–H	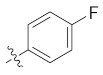	0.56 ± 0.02	–
2	–OH	–OH	–H	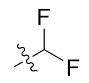	>43.83 (39.82%)	–
3	–OH	–OH	–H	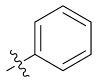	>39.37 (35.75%)	–
4a	–OH	–OH	–H	–CH_3_	2.65 ± 0.25	–
4	–OH	–OH	–H	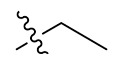	–	0.15 ± 0.01
5	–OH	–OH	–H	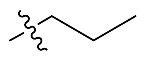	–	3.77 ± 0.58
6	–OH	–OH	–H	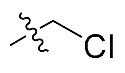	0.49 ± 0.32	–
7	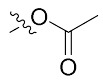	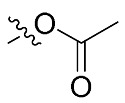	–H	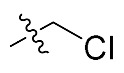	–	0.02 ± 0.01
8	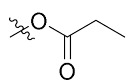	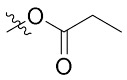	–H	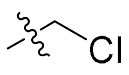	–	6.86 ± 0.04
9	–OH	–H	–H	–CH_3_	1.25 ± 0.89	–
10	–OH	–H	–H	–OCH_3_	–	>48.54 (33.58%)
11	–OH	–H	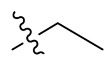	–CH_3_	1.28 ± 1.07	–
12	–OH	–H	–CH_3_	–CH_3_	1.07 ± 0.33	–
13	–OH	–H	–H	–CF_3_	–	7.75 ± 7.23
14	–OH	–H	–Cl	–CH_3_	0.03 ± 0.02	–
15	–OH	–H	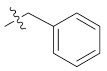	–CH_3_	3.60 ± 0.11	–
16	–OH	–H	–F	–CH_3_	0.24 ± 0.21	–
17	–OH	–H	–CN	–CH_3_	0.03 ± 0.02	–
18	–OH	–H	–H	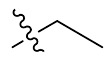	0.03 ± 0.01	–
19	–OH	–H	–H	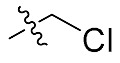	4.22 ± 3.49	–
20	–OH	–H	–H	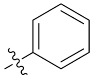	>41.97 (34.41%)	–
21	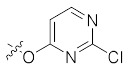	H	H	–CH_3_	0.02 ± 0.01	–
22	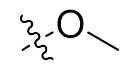	–H	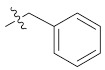	–CH_3_	–	9.67 ± 2.59
23	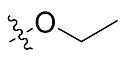	–H	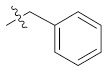	–CH_3_	–	0.76 ± 0.70
24	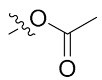	–H	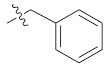	–CH_3_	>35.67 (32.82%)	–
25	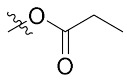	–H	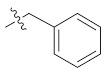	–CH_3_	11.98 ± 9.47	–

## Data Availability

The data presented in this study are available on request from the corresponding author.

## Supplementary Materials

The following supporting information can be downloaded at: www.mdpi.com/10.3390/polym14102021/s1, Figures S1–S72: The NMR spectra and ESI-MS spectroscopy dates of compounds 1–25; Figures S73–S75: The relationship between concentrate and inhibition rate of compounds 1–25. References [52,53,54,55,56,57,58,59,61] are cited in the supplementary materials.
