# Supplementary material for: Synthesis of Coumarin Derivatives: A New Class of Coumarin-Based G Protein-Coupled Receptor Activators and Inhibitors
Source: Polymers (Basel). 2022 May 15;14(10):2021. doi: 10.3390/polym14102021 (PMC9147790; doi:10.3390/polym14102021)
Supplement: Supplementary file 1 [file polymers-14-02021-s001.zip › polymers-1657567-supplementary materials.pdf]

polymers

Supplementary Material: Synthesis of Coumarin Derivatives: A New Class of Coumarin-Based G Protein-Coupled Receptor Activators and Inhibitors

Zhe Fu,[1] Linjie Zhang,[1] Sijin Hang,[1] Shiyi Wang,[2] Na Li,[1] Xiaojing Sun,[1] Zian Wang,[1] Ruilong Sheng,[3] Fang Wang,[4] Wenhui Wu,[1] and Ruihua Guo [1,5,6,*]

[1] College of Food Science and Technology, Shanghai Ocean University, Shanghai 201306, China; fzshou2019@163.com (Z.F); ljzhang20010428@163.com (L.Z.); sjhang2022@163.com (S.H.); ln16658@163.com (N.L.); sunxj1997@163.com (X.S.); wzamengnan1999@163.com (Z.W.); whwu@shou.edu.cn (W.W.)
[2] AIEN Institute, Shanghai Ocean University, Shanghai 201306, China; wjcxx3111@163.com (S.W.)
[3] CQM - Centro de Química da Madeira, Campus da Penteada, Universidade da Madeira, 9000-390 Funchal, Portugal; ruilong.sheng@staff.uma.pt (R.Z.)
[4] School Basic Medicine, Shanghai University of Medicine and health Science, Shanghai 201318, China; wangf_18@sumhs.edu.cn (F.W.)
[5] Laboratory of Quality and Safety Risk Assessment for Aquatic Product on Storage and Preservation (Shanghai), Ministry of Agriculture, Shanghai 201306, China;
[6] Engineering Research Center of Aquatic-Product Processing & Preservation, Shanghai 201306, China;
* Correspondence: rhguo@shou.edu.cn (R.G);

Table of Contents

1 *4-(4-Fluorophenyl)-7, 8-dihydroxy-2 H-chromen-2-one (compound **1**)*.1
2. *4-(Difluoromethyl)-7, 8-dihydroxy-2H-chromen-2-one (compound **2**)*.2
3. *7, 8-Dihydroxy-4-phenyl-2H-chromen-2-one (compound **3**)*.3
4. *4-Ethyl-7, 8-dihydroxy-2H-chromen-2-one (compound **4**).*
5. *4-(Chloromethyl)-7, 8-dihydroxy-2H-chromen-2-one (compound **5**)*.8
6. *7,8-Dihydroxy-4-propyl-2H-chromen-2-one (compound **6**)*.9
7. *4-(Chloromethyl)-2-oxo-2H-chromene-7, 8-diyl dipropionate (compound **7**)*.11
8. *4-(Chloromethyl)-2-oxo-2H-chromene-7, 8-diyl diacetate (compound **8**)*.12
9. *7-Hydroxy-4-methyl-2H-chromen-2-one (compound **9**)*.14
10. *7-Hydroxy-4-methoxy-2H-chromen-2-one (compound **10**)*.15
11. *3-Ethyl-7-hydroxy-4-methyl-2H-chromen-2-one (compound **11**)*.16
12. *7-Hydroxy-3, 4-dimethyl-2H-chromen-2-one (compound **12**)*..18
13. *7-Hydroxy-4-(trifluoromethyl)-2H-chromen-2-one (compound **13**)*.19
14. *3-Chloro-7-hydroxy-4-methyl-2H-chromen-2-one (compound **14**)*.21
15. *3-Benzyl-7-hydroxy-4-methyl-2H-chromen-2-one (compound **15**)*.21
16. *3-Fluoro-7-hydroxy-4-methyl-2H-chromen-2-one (compound **16**)*.23
17. *7-Hydroxy-4-methyl-2-oxo-2H-chromene-3-carbonitrile (compound **17**)*.25
18. *4-Ethyl-7-hydroxy-2H-chromen-2-one (compound **18**)*.26
19. *4-(Chloromethyl)-7-hydroxy-2H-chromen-2-one (compound **19**)*.28
20. *7-Hydroxy-4-phenyl-2H-chromen-2-one (compound **20**)*.29
21. *7–((2–chloropyrimidin–4–yl)oxy)–4–methyl–2H–chromen–2–one* (*compound* **21**).31
22. *3-Benzyl-7-methoxy-4-methyl-2H-chromen-2-one (compound **22**)*.32
23. *4-Benzyl-7-ethoxy-4-methyl-2H-chromen-2-one (compound **23**)*.34
24. *3-Benzyl-4-methyl-2-oxo-2H-chromen-7-yl acetate (compound **24**)*.35
25. *3-Benzyl-4-methyl-2-oxo-2H-chromen-7-yl propionate (compound **25**)*.37

26. *Biological Evaluation of The Synthesized Coumarin Derivatives at GPCRs in vitro*38

NMR spectra and ESI-MS spectroscopy dates were shown as following:

1. 4-(4-Fluorophenyl)-7, 8-dihydroxy-2 H-chromen-2-one (compound 1).

White amorphous powder, yield: 32%. ^{1}H NMR (400 MHz, CD_3OD) δ: 7.48–7.38 (2H, m), 7.18 (2H, t, *J* = 8.7 Hz), 6.83–6.71 (2H, m), 6.08 (1H, s). ^{13}C NMR (101 MHz, CD_3OD), δ: 168.7, 166.3 (2C), 160.8, 153.5, 147.4, 136.3, 135.7, 134.3 (2C), 121.9, 119.9, 119.7, 116.2, 114.2. ESI-MS: *m*/*z* 273 $[M+H]^{+}$; calcd for $C_{15}H_9FO_4$, 272.23.

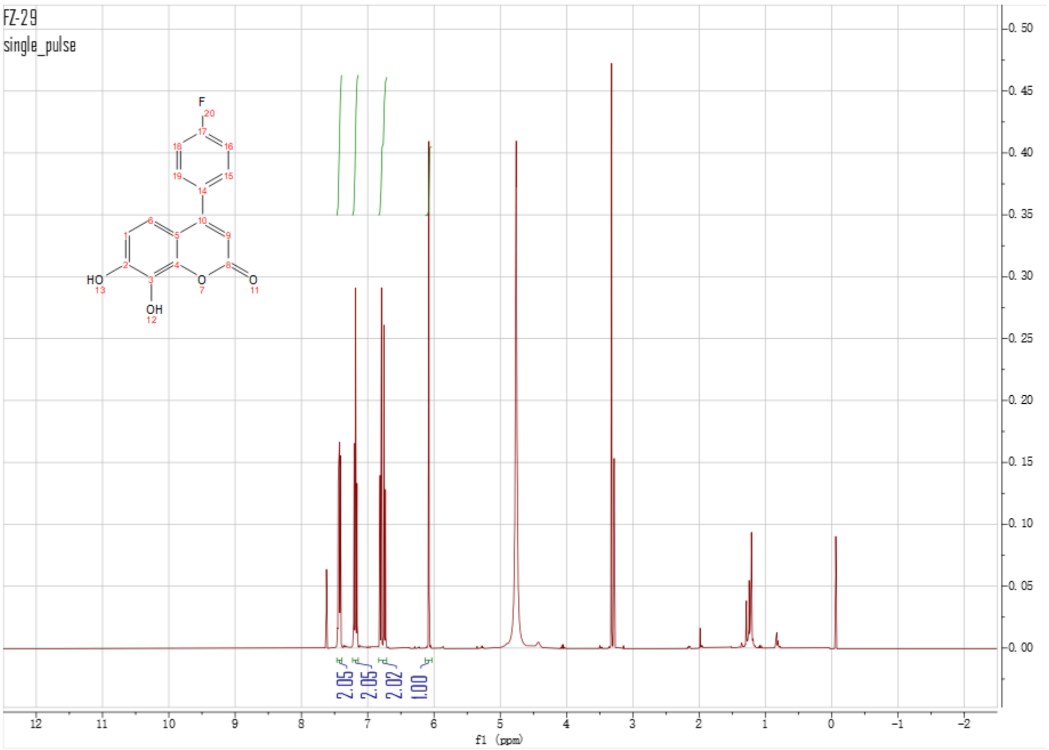

Figure S1. ^{1}H NMR of compound **1.**

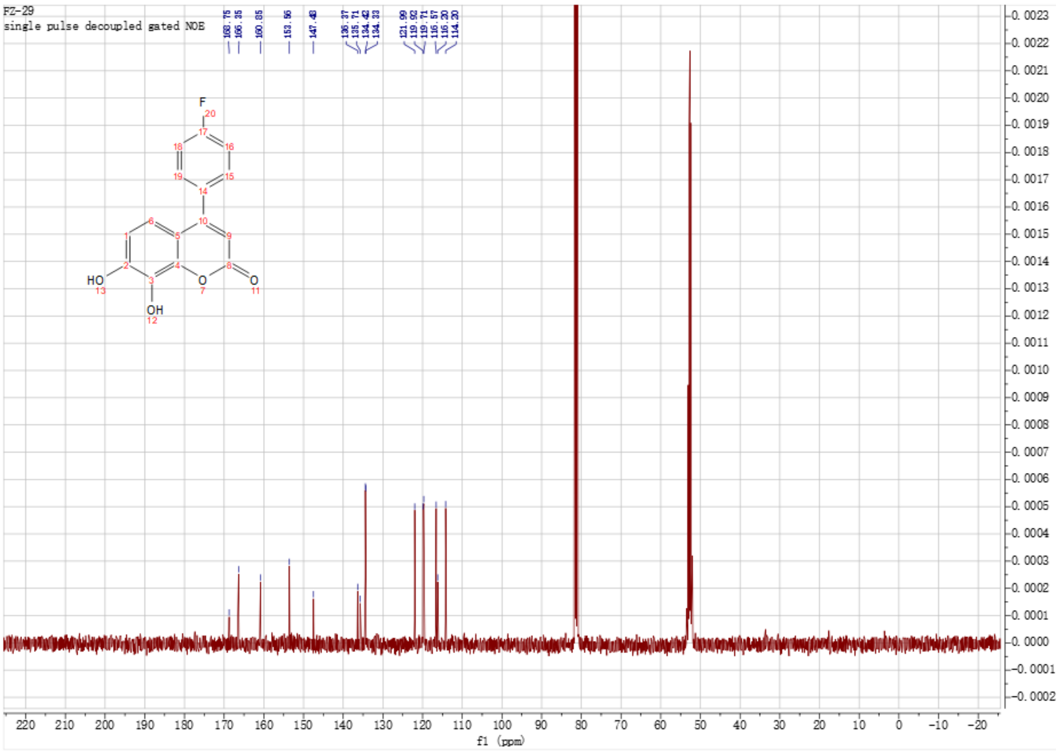

Figure S2. ^{13}C NMR of compound **1**.

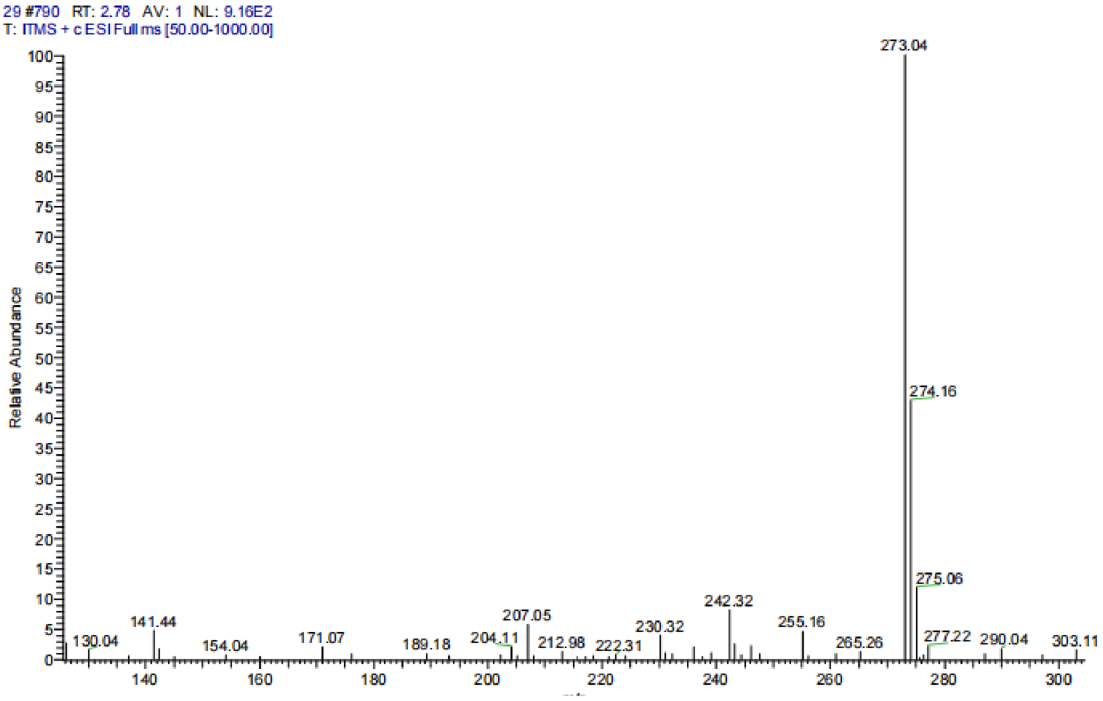

Figure S3. ESI-MS of compound **1**.

2. 4-(Difluoromethyl)-7, 8-dihydroxy-2H-chromen-2-one (compound 2).

White amorphous powder, yield: 21%. ^{1}H NMR (400 MHz, CD_3OD) δ: 7.05 (1H, d, J = 8.7 Hz), 6.98 (1H, t, J_F = 53.6 Hz), 6.80 (1H, d, J = 8.7 Hz), 6.39 (1H, s). ^{13}C NMR (101 MHz, CD_3OD) δ: 160.8, 150.1, 146.9 (t, $J_{C\text{-}F}$ = 22.0 Hz), 143.9, 132.6, 114.8, 112.4, 109.4, 108.2. ESI-MS: m/z 229 $[M+H]^+$; calcd for $C_{10}H_6F_2O_4$, 228.15.

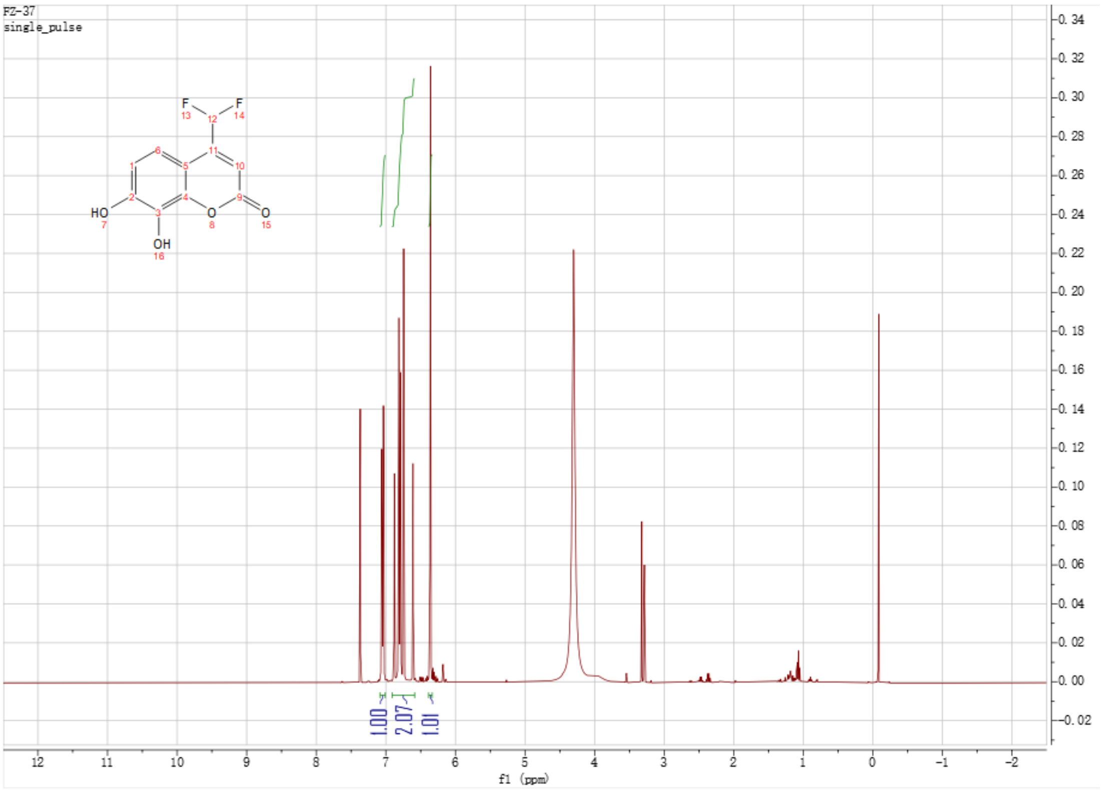

Figure S4. ^{1}H NMR of compound **2**.

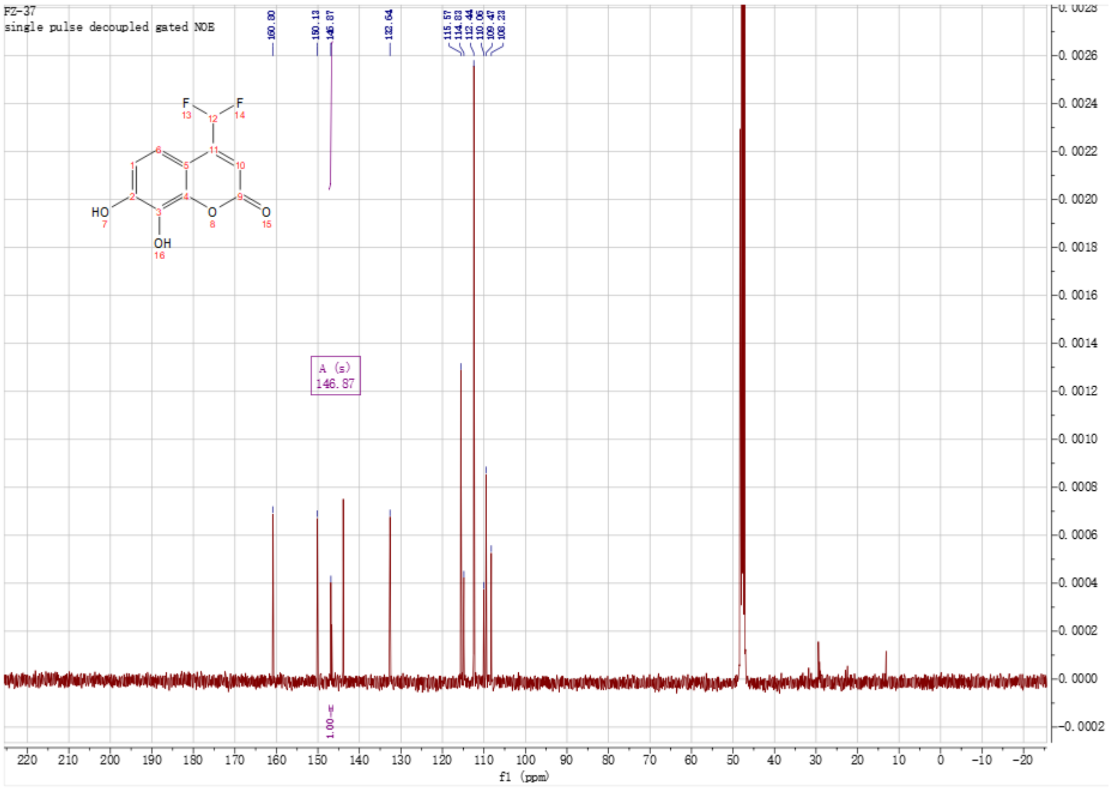

Figure S5. ^{13}C NMR of compound **2**.

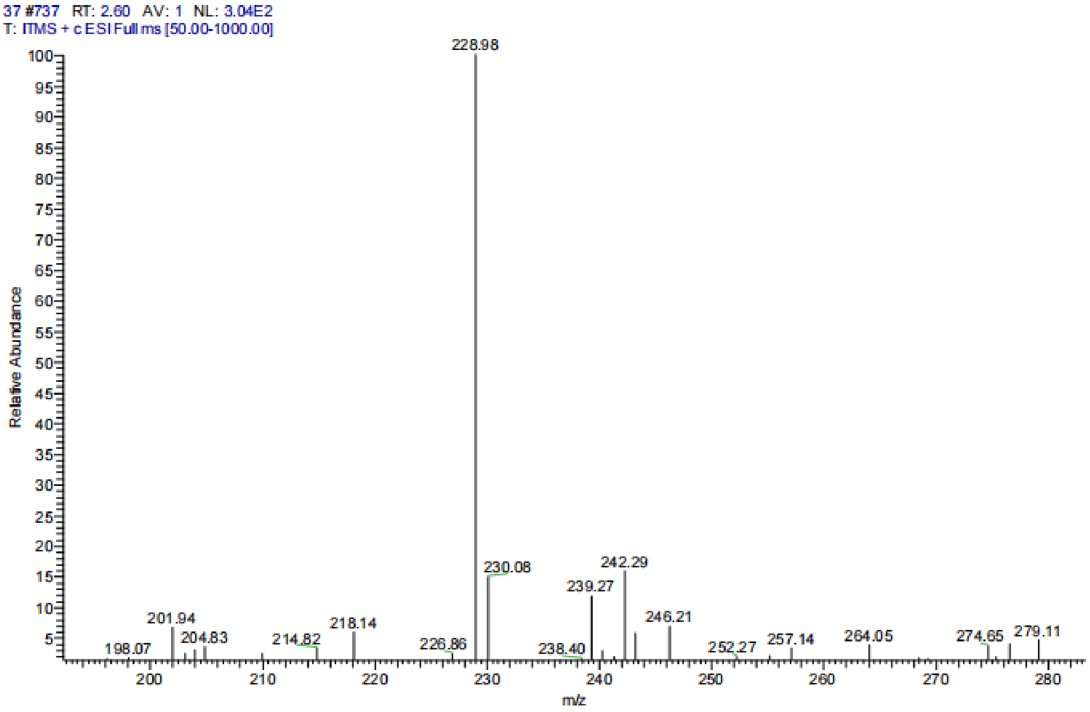

Figure S6. ESI-MS of compound **2**.

3. 7,8-. Dihydroxy-4-phenyl-2H-chromen-2-one (compound 3).

White amorphous powder, yield: 22%. ^{1}H NMR (400 MHz, CD_3OD) δ: 7.46-7.42 (3H, m), 7.39-7.34 (2H, m), 6.83-6.67 (2H, m), 6.05 (s, 1H). ^{13}C NMR (101 MHz, CD_3OD) δ: 162.3, 157.8, 149.6, 143.7, 135.8, 132.5, 129.4, 128.6 (2C), 128.3 (2C), 118.0, 112.3, 112.2, 109.9. ESI-MS: *m/z* 255 [M+H]$^+$; calcd for $C_{15}H_{10}O_4$, 254.24. **Error! Reference source not found.**

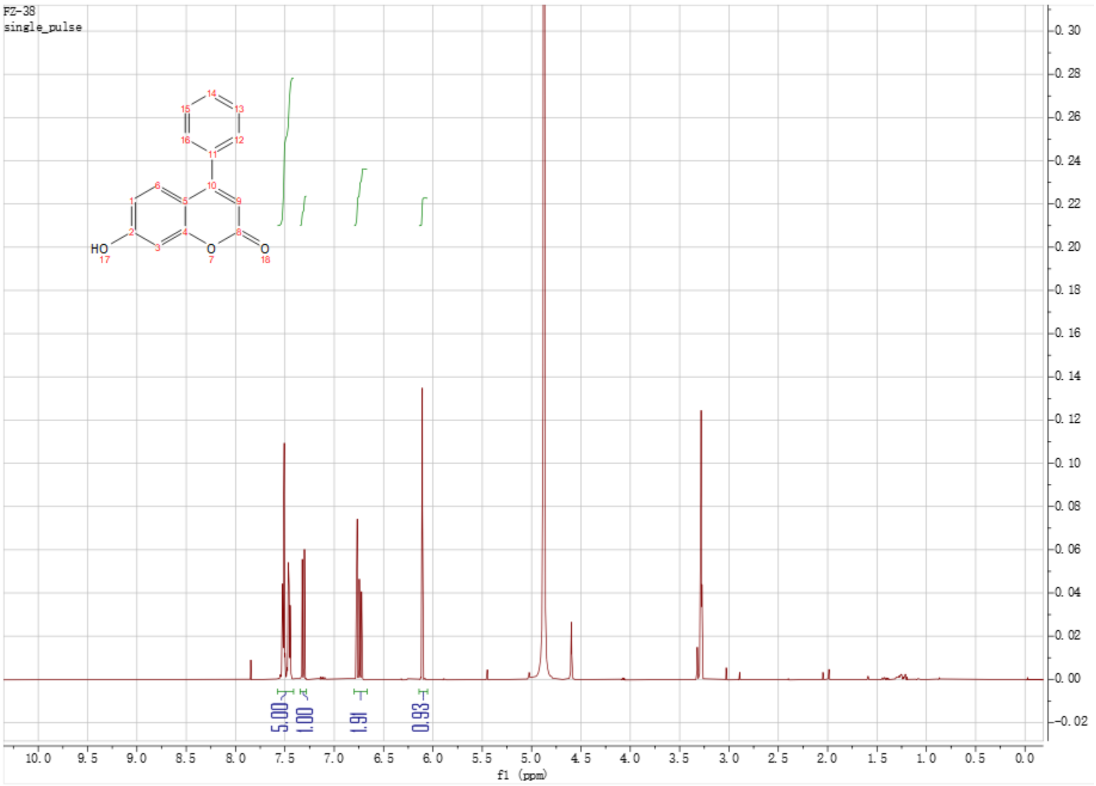

Figure S7. ^{1}H NMR of compound **3**.

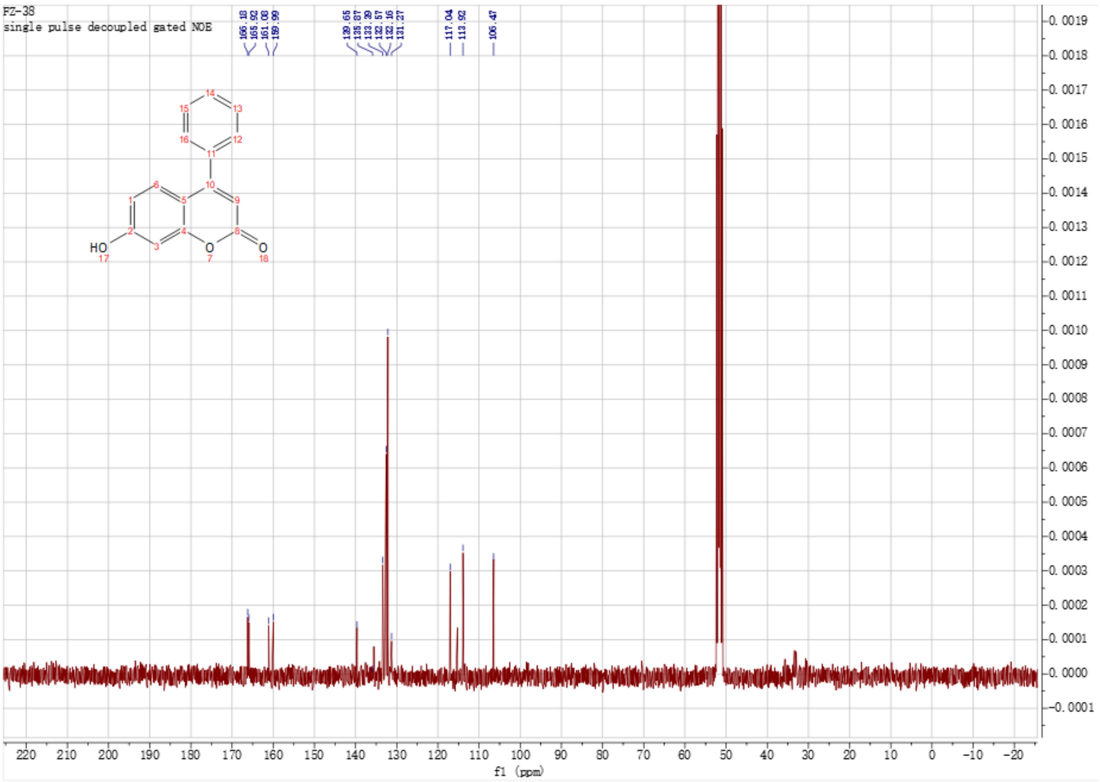

Figure S8. ^{13}C NMR of compound **3**.

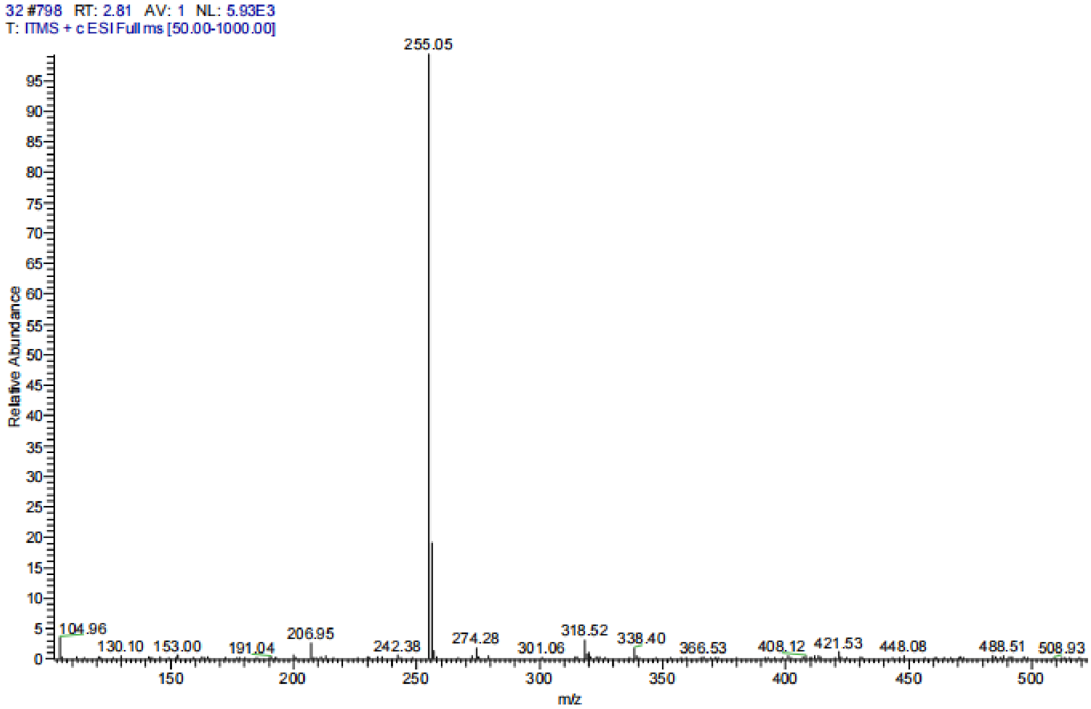

Figure S9. ESI-MS of compound **3**.

4. 4-Ethyl-7, 8-dihydroxy-2H-chromen-2-one (compound 4).

White amorphous powder, yield: 28%. ^{1}H NMR (400 MHz, $CDCl_3$) δ: 7.11 (1H, d, *J* = 8.6 Hz), 6.88 (1H, d, *J* = 8.6 Hz), 6.12 (1H, m), 2.83-2.77 (2H, m), 1.34-1.30 (3H, m). ^{13}C NMR (101 MHz, $CDCl_3$) δ: 167.0, 164.3, 152.9, 146.9, 136.0, 119.2, 116.6, 116.5, 112.3, 28.9, 16.3. ESI-MS: *m*/*z* 207 [M+H]$^{+}$; calcd for $C_{11}H_{10}O_4$, 206.20.

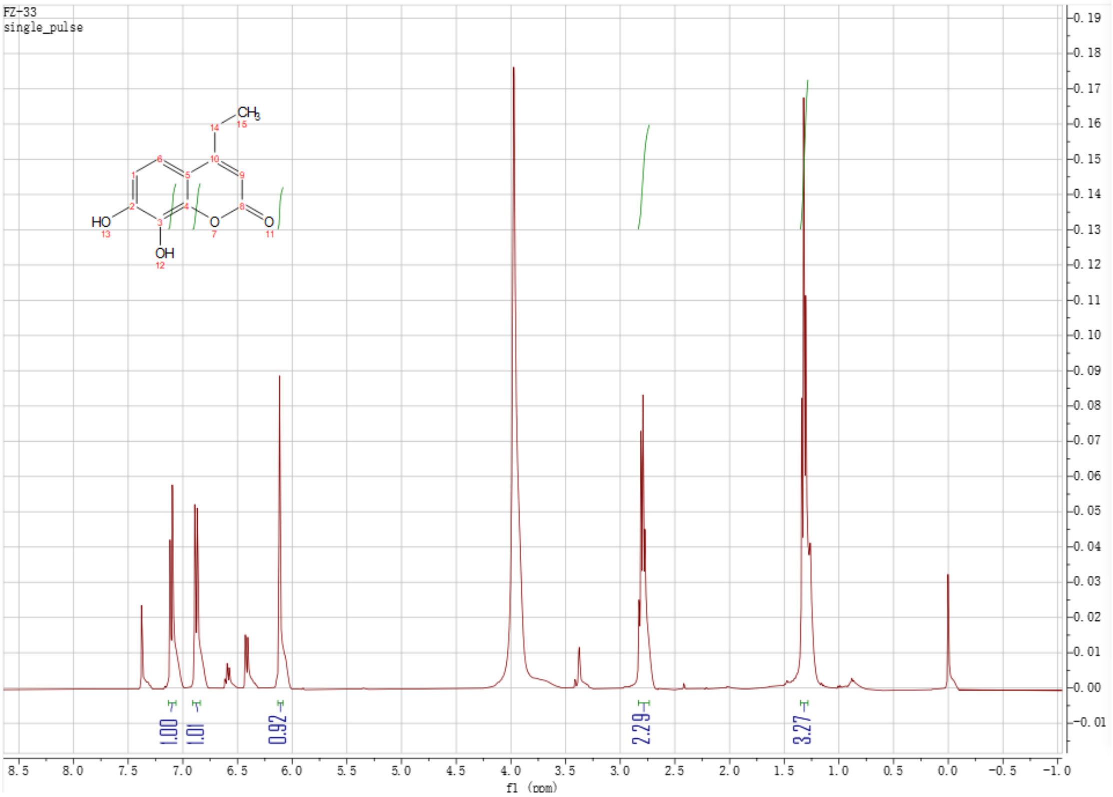

Figure S10. ^{1}H NMR of compound **4**.

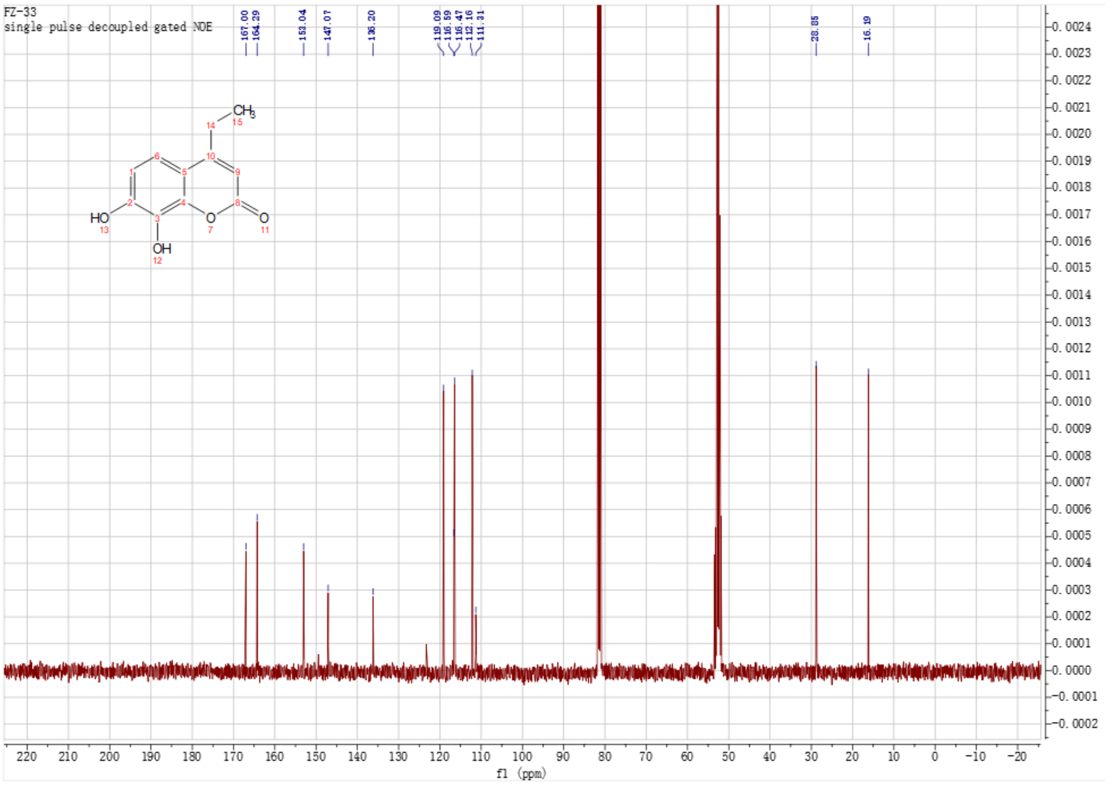

Figure S11. ^{13}C NMR of compound **4**.

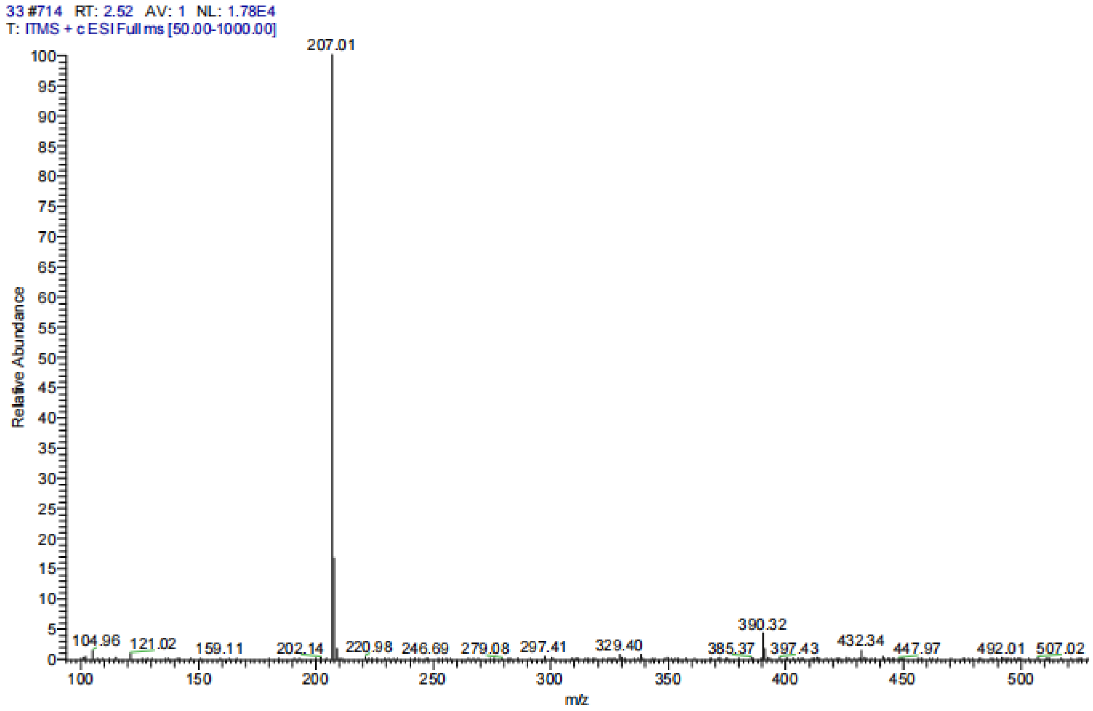

Figure S12. ESI-MS of compound **4**.

5. 4-(Chloromethyl)-7, 8-dihydroxy-2H-chromen-2-one (compound 5).

White amorphous powder, yield: 48%. ^{1}H NMR (400 MHz, CD_3OD) δ: 7.19 (1H, d, *J* = 8.7 Hz), 6.84 (1H, d, *J* = 8.7 Hz), 6.37 (1H, s), 4.80 (2H, s). ^{13}C NMR (100 MHz, CD_3OD), δ: 161.5, 152.1, 149.6, 143.5, 132.4, 115.1, 112.1, 110.5, 110.1, 40.8. ESI-MS: *m*/*z* 227 [M+H]$^{+}$; calcd for $C_{10}H_7ClO_4$, 226.98.**Error! Reference source not found.**

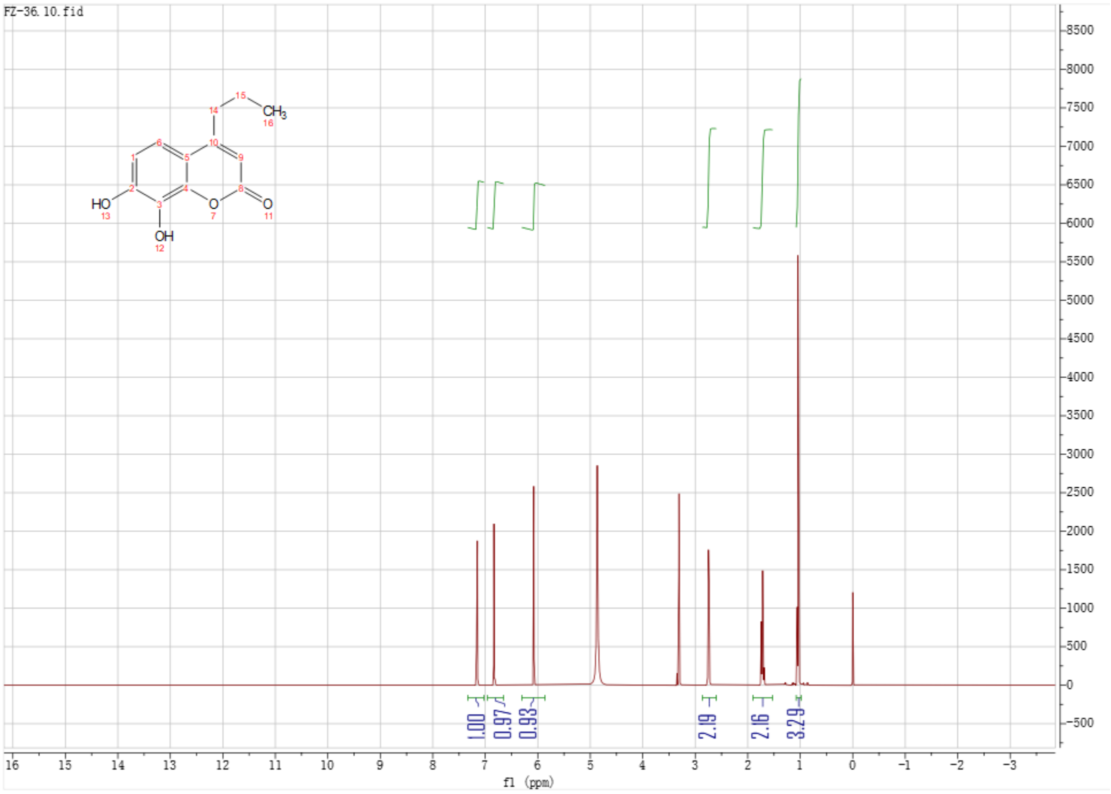

Figure S13. ^{1}H NMR of compound **5**.

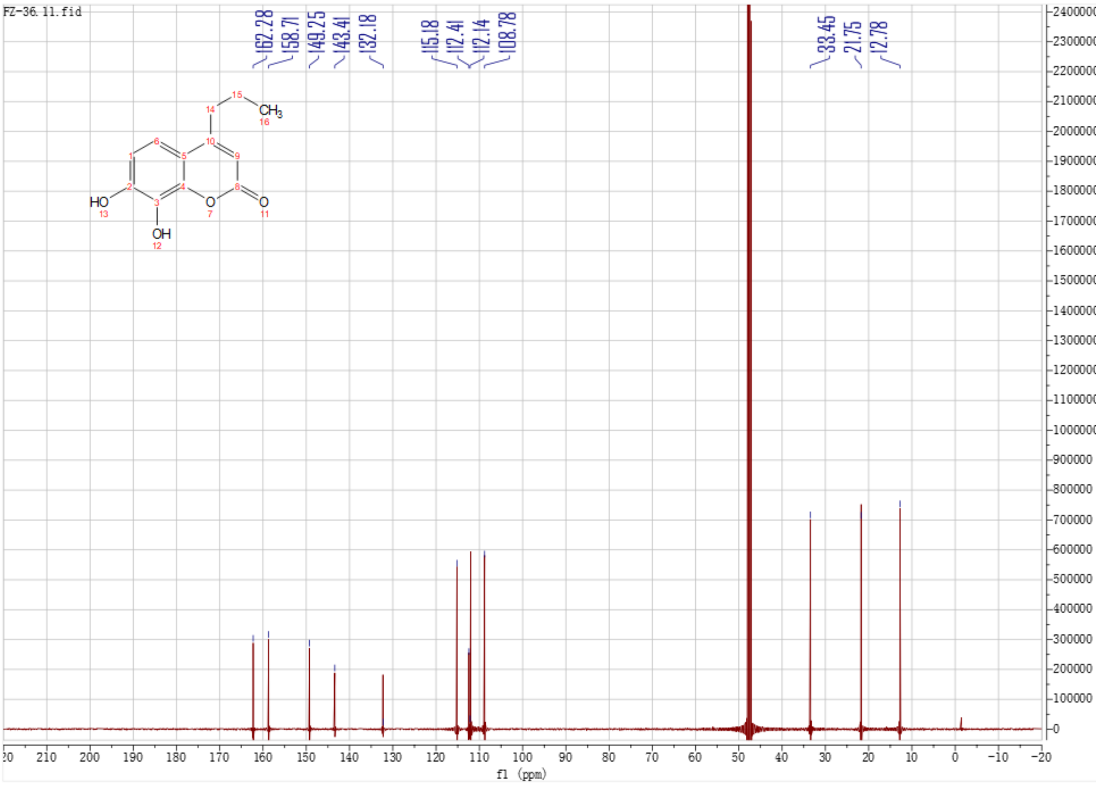

Figure S14. ^{13}C NMR of compound **5**.

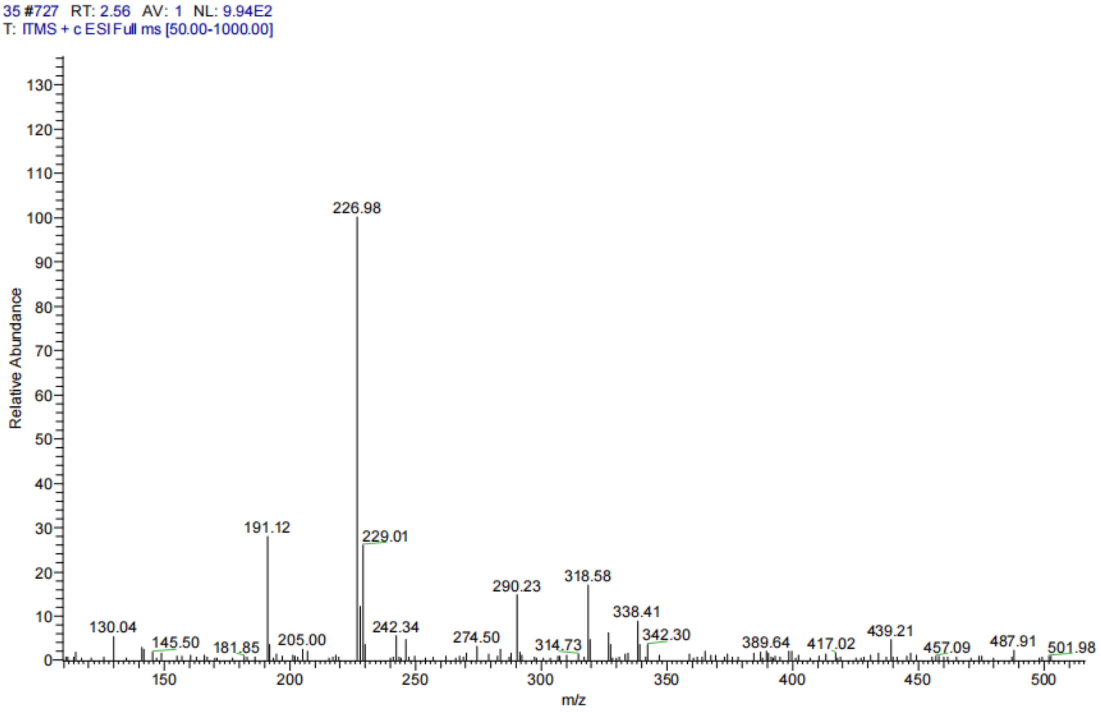

Figure S15. ESI-MS of compound **5**.

6.7,8-. Dihydroxy-4-propyl-2H-chromen-2-one (compound 6).

White amorphous powder, yield: 36%. ^{1}H NMR (400 MHz, CD_3OD), δ: 7.16 (1H, d, *J* = 8.7 Hz), 6.83 (1H, d, *J* = 8.7 Hz), 6.08 (1H, s), 2.77-2.73 (2H, t, *J* =8.0 Hz), 1.76-1.69 (2H, m), 1.06-1.02 (3H, t, *J* = 7.4 Hz). ^{13}C NMR (100 MHz, CD_3OD), δ: 162.3, 158.7, 149.2, 143.4, 132.3, 115.1, 112.4, 112.0, 108.7, 33.4, 21.7, 12.7. ESI-MS: *m/z* 221 [M+H] $^+$; calcd for $C_{12}H_{12}O_4$, 221.04.**Error! Reference source not found.**

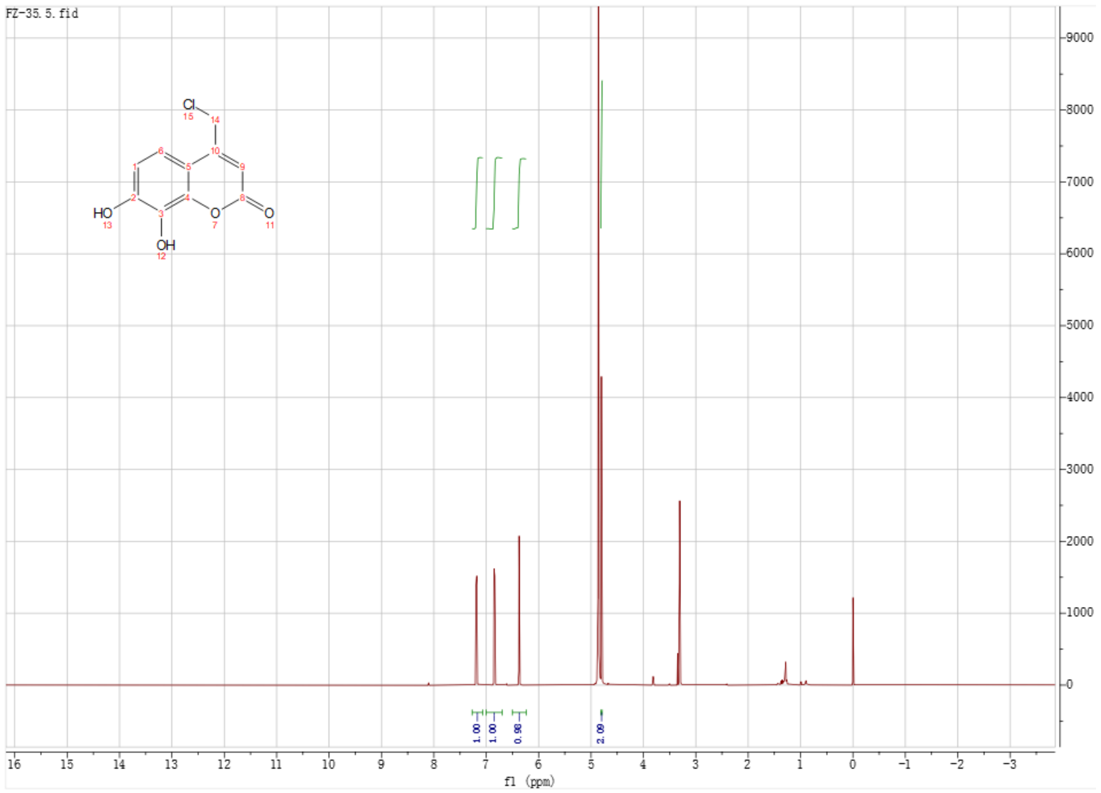

Figure S16. ^{1}H NMR of compound **6**.

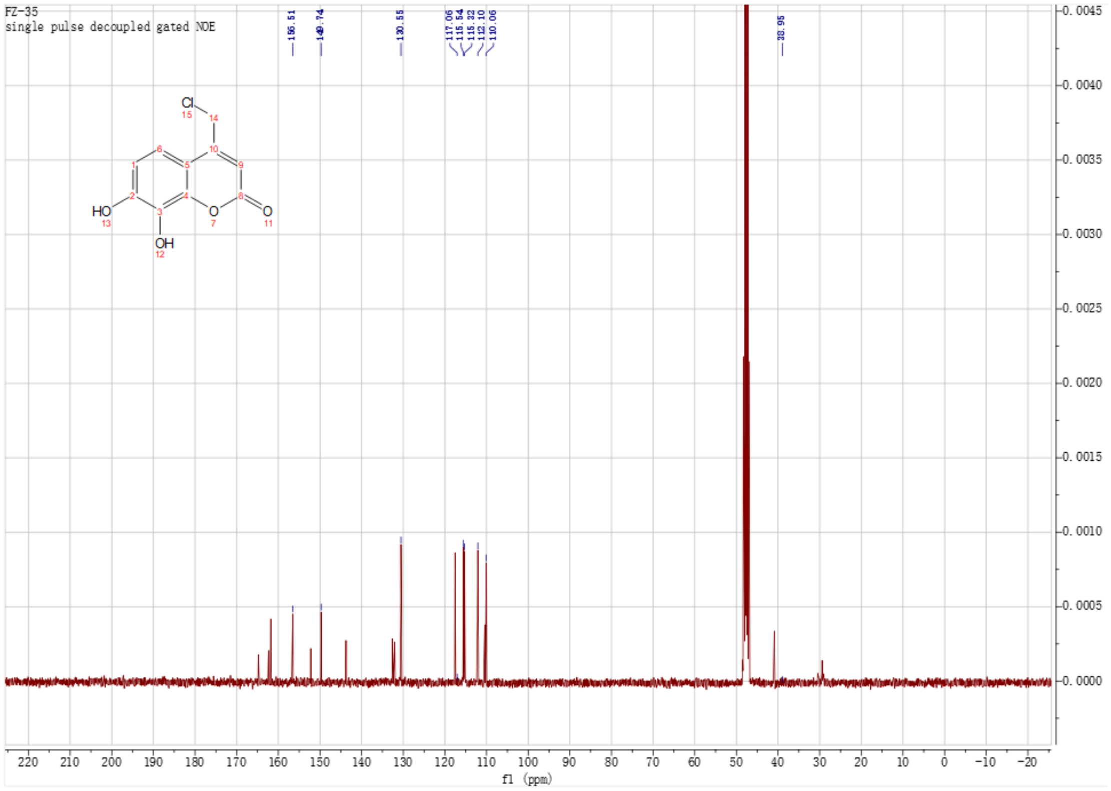

Figure S17. ^{13}C NMR of compound **6**.

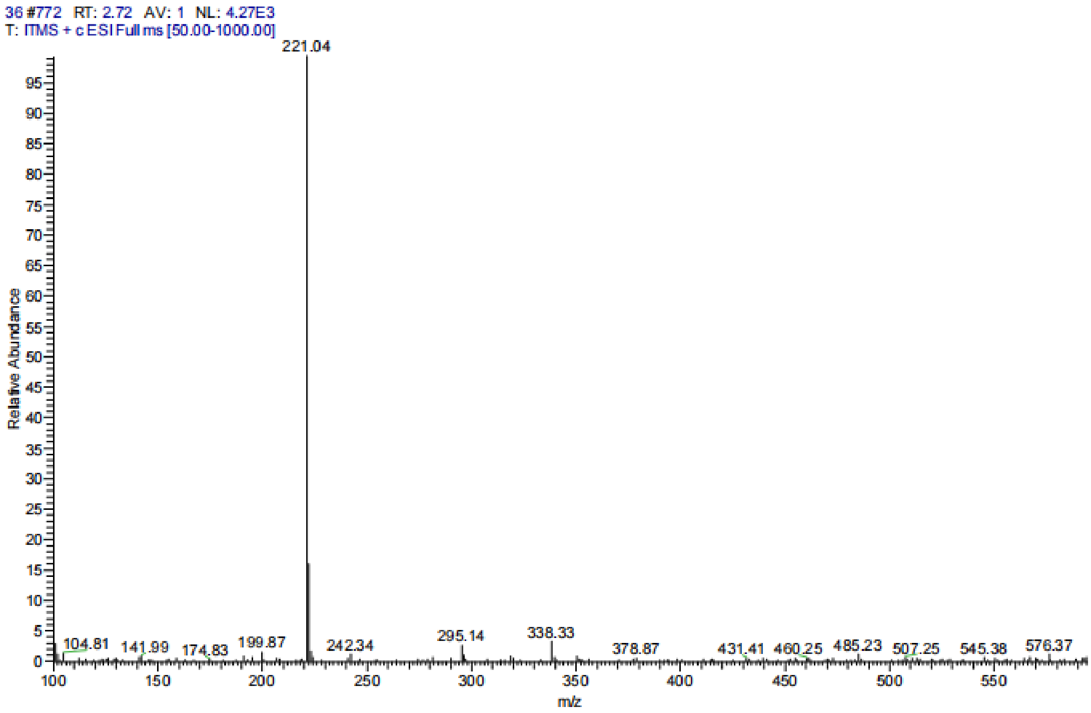

Figure S18. ESI-MS of compound **6**.

7. 4-(Chloromethyl)-2-oxo-2H-chromene-7, 8-diyl dipropionate (compound 7).

White amorphous powder, yield: 62%. ^{1}H NMR (400 MHz, $CDCl_3$) δ: 7.53 (d, *J* = 8.8 Hz, 1H), 7.16 (d, *J* = 8.8 Hz, 1H), 6.51 (s, 1H), 4.60 (s, 2H), 2.38 (s, 3H), 2.31 (s, 3H). ^{13}C NMR (100 MHz, $CDCl_3$) δ: 167.9, 167.5, 158.9, 149.3, 147.2, 145.7, 130.8, 121.6, 119.1, 116.3, 115.7, 41.2, 20.8, 20.4. ESI-MS: *m*/*z* 311 [M+H] $^+$; calcd for $C_{19}H_{18}O_3$, 310.69.**Error! Reference source not found.**

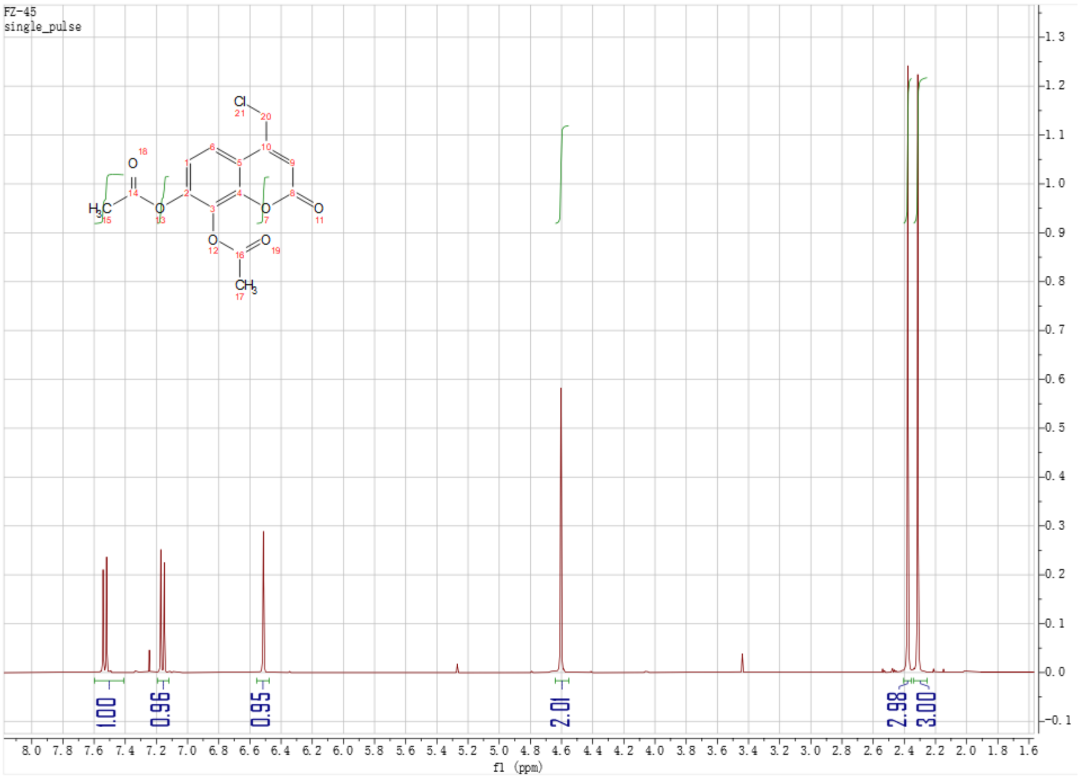

Figure S19. 1H NMR of compound **7**.

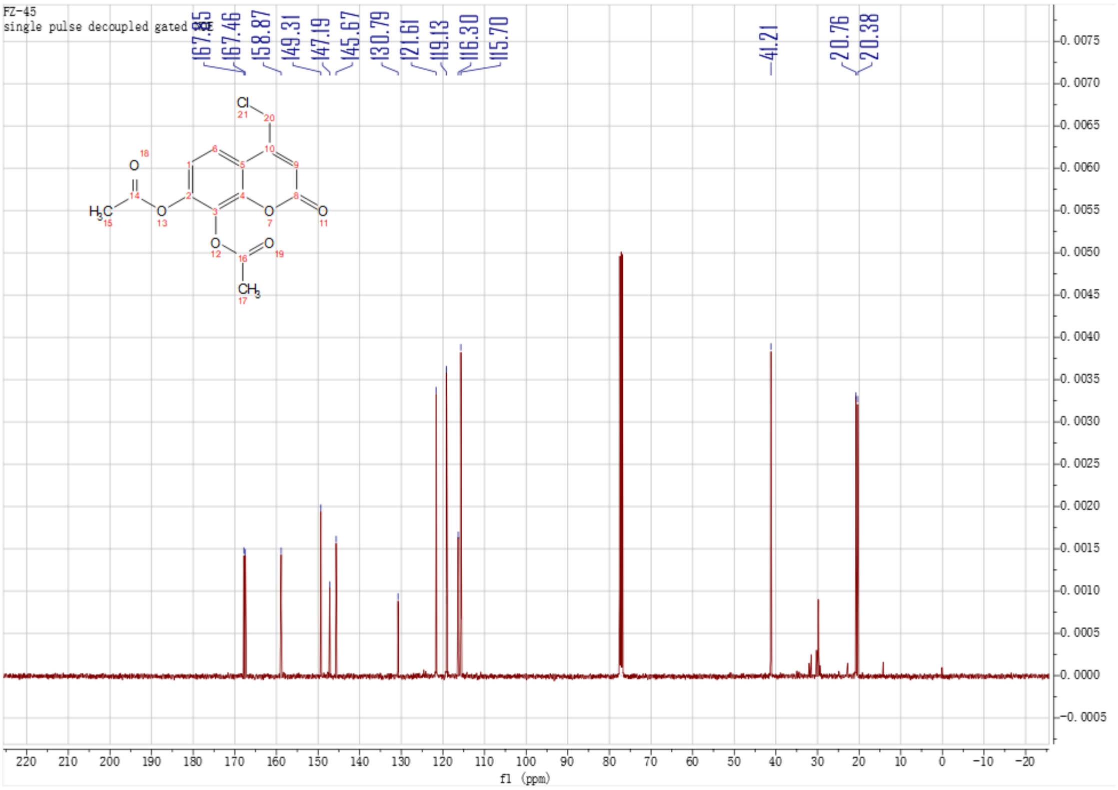

Figure S20. ^{13}C NMR of compound **7**.

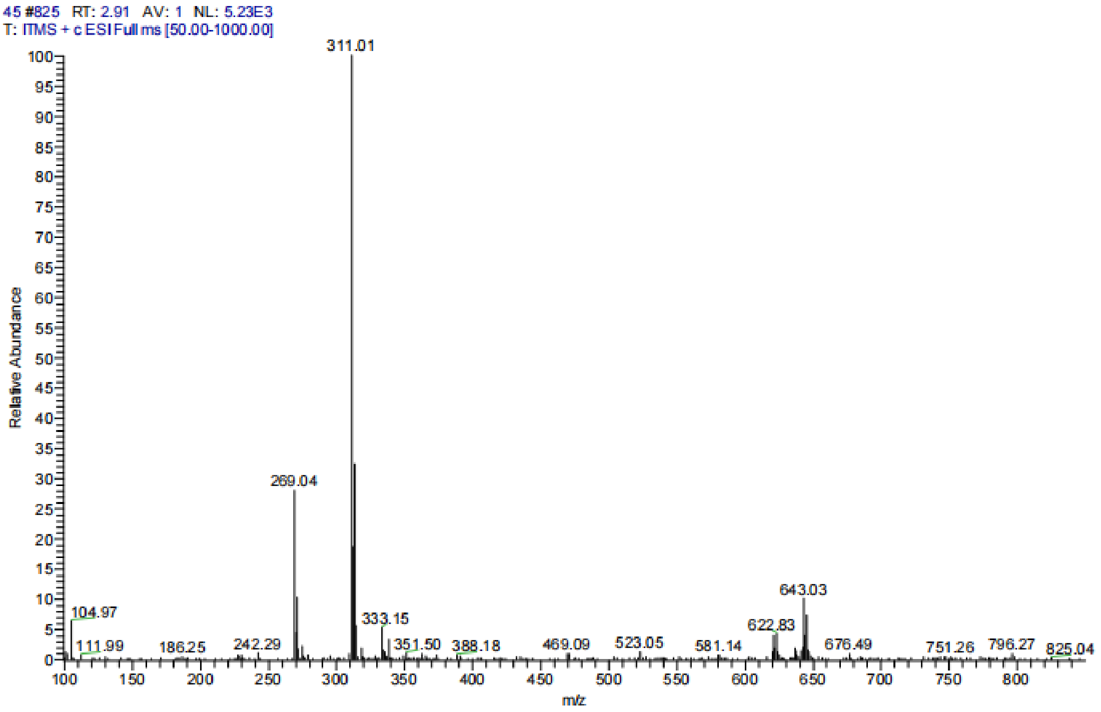

Figure S21. ESI-MS of compound 7.

8. 4-(Chloromethyl)-2-oxo-2H-chromene-7, 8-diyl diacetate (compound 8).

White amorphous powder, yield: 68%. ^{1}H NMR (400 MHz, $CDCl_3$) δ: 7.50 (1H, d, *J* = 8.8 Hz), 7.11 (1H, d, *J* = 8.8 Hz), 6.47 (1H, d, *J* = 1.1 Hz), 4.58 (2H, d, *J* = 1.0 Hz), 2.61 (2H, q, *J* = 7.6 Hz), 2.53 (2H, q, *J* = 7.6 Hz), 1.22-1.16 (6H, m). ^{13}C NMR (400 MHz, $CDCl_3$) δ: 171.42,

171.09, 158.99, 148.88, 145.77, 144.56, 131.79, 125.85, 122.35, 119.13, 115.77, 41.16, 29.79, 27.21, 9.22 (2 C). ESI-MS: *m*/*z* 338 [M+H] $^{+}$; calcd for $C_{14}H_{11}ClO_6$, 338.5.

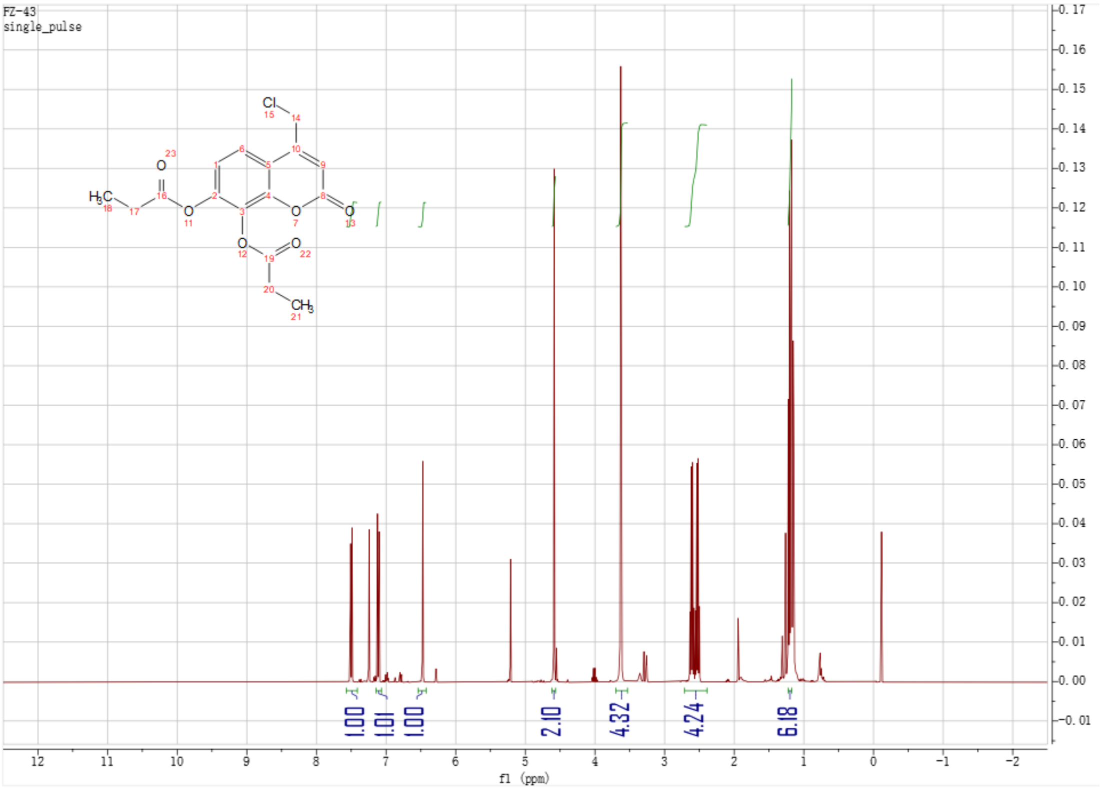

Figure S22. ^{1}H NMR of compound **8**.

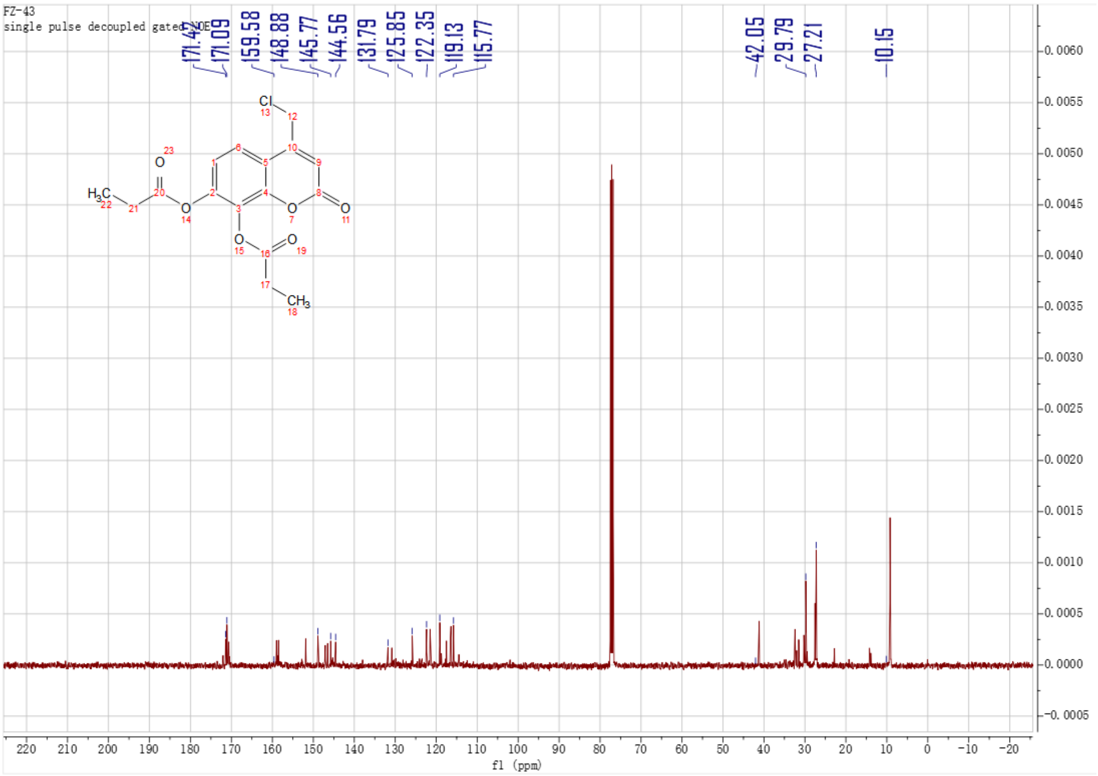

Figure S23. ^{13}C NMR of compound **8**.

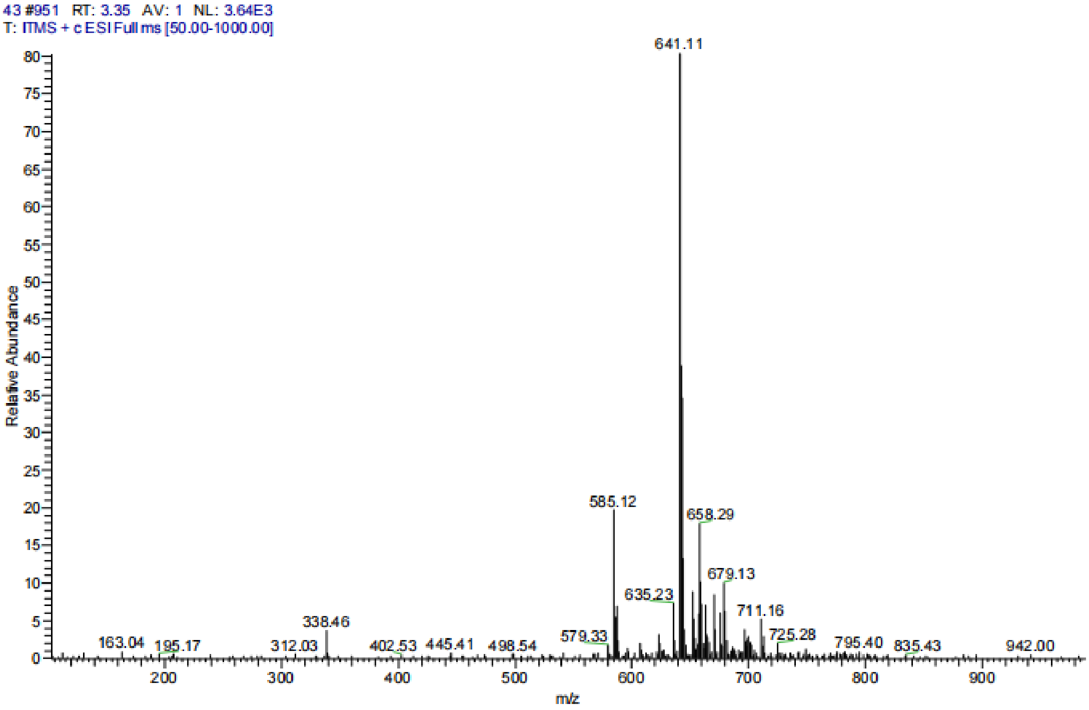

Figure S24. ESI-MS of compound **8**.

9. 7-Hydroxy-4-methyl-2H-chromen-2-one (compound 9).

White amorphous powder, yield: 72%. ^{1}H NMR (500 MHz, $CDCl_3$) δ: 7.51 (1H, d, *J* = 8.7 Hz), 6.95 (1H, d, *J* = 2.4 Hz), 6.86 (1H, dd, *J* = 8.7, 2.4 Hz), 6.17 (1H, d, *J* = 1.2 Hz), 2.43 (3H, d, *J* = 1.2 Hz). ^{13}C NMR (125 MHz, $CDCl_3$) δ: 162.5, 161.7, 155.2, 154.7, 126.1, 113.0, 112.5, 109.9, 102.1, 17.3. ESI-MS: *m*/*z* 177 [M+H] $^{+}$; calcd for $C_{10}H_8O_3$, 176.17.**Error! Reference source not found.**

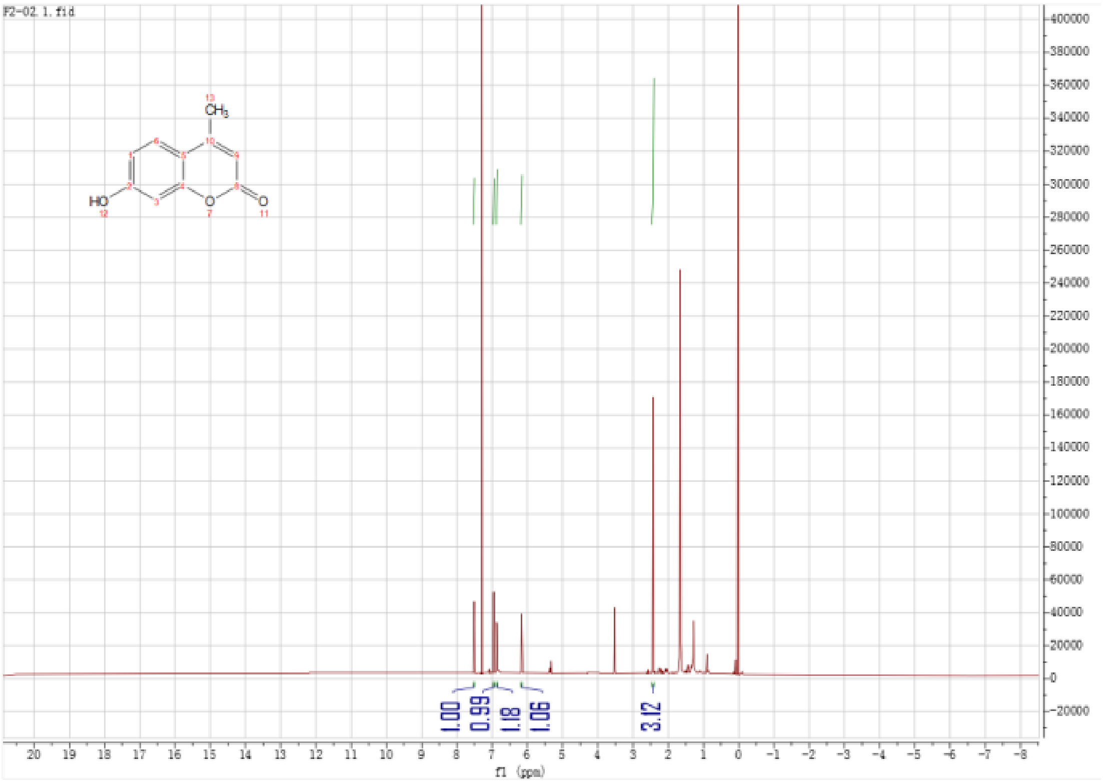

Figure S25. ^{1}H NMR of compound **9**.

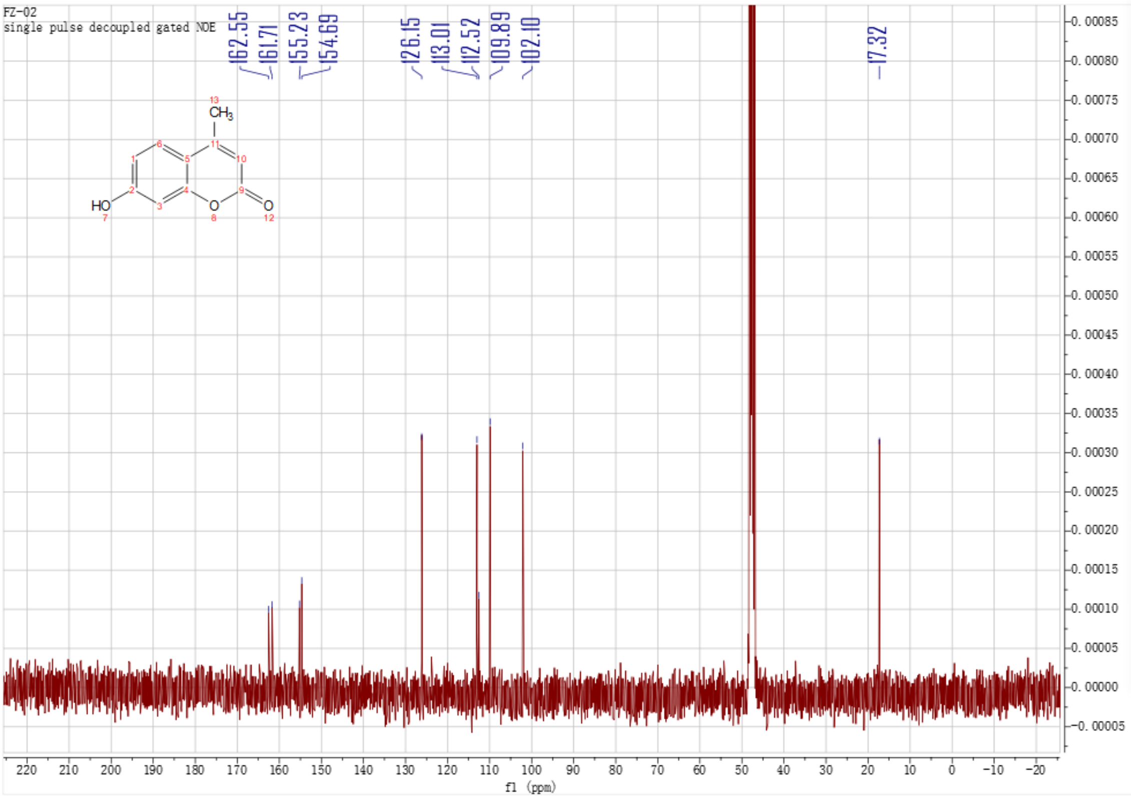

Figure S26. ^{13}C NMR of compound **9**.

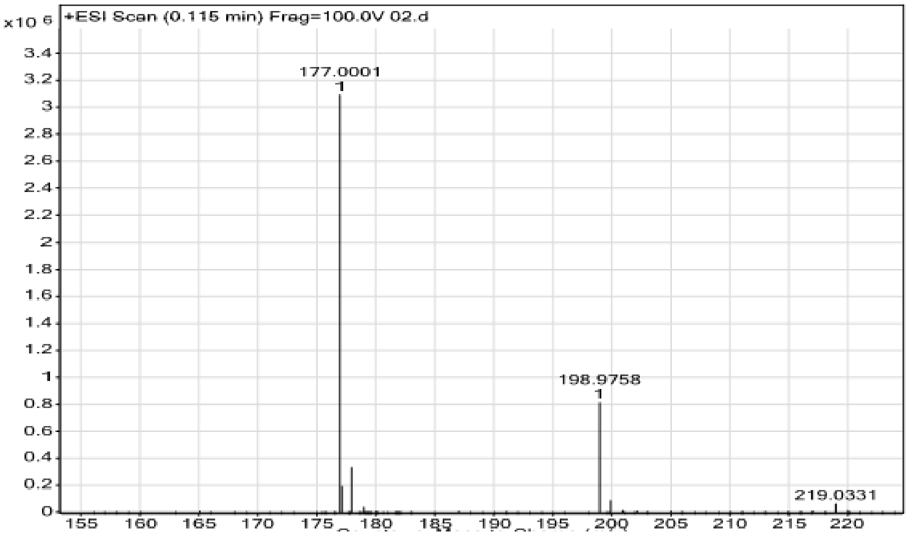

Figure S27. ESI-MS of compound **9**.

10. 7-Hydroxy-4-methoxy-2H-chromen-2-one (compound 10).

White amorphous powder, yield: 34%. ^{1}H NMR (600 MHz, CD_3OD) δ: 7.56 (1H, d, *J* = 8.7 Hz), 6.82 (1H, dd, *J* = 8.7, 1.9 Hz), 6.75 (1H, d, *J* = 1.9 Hz), 6.31 (1H, s), 3.53 (3H, s). ^{13}C NMR (125 MHz, CD_3OD) δ: 162.5, 161.6, 155.4, 153.6, 125.3, 113.1, 110.0, 107.6, 102.3, 58.0. ESI-MS: *m*/*z* 207 [M+H] $^{+}$; calcd for $C_{10}H_8O_4$, 206.20.**Error! Reference source not found.**

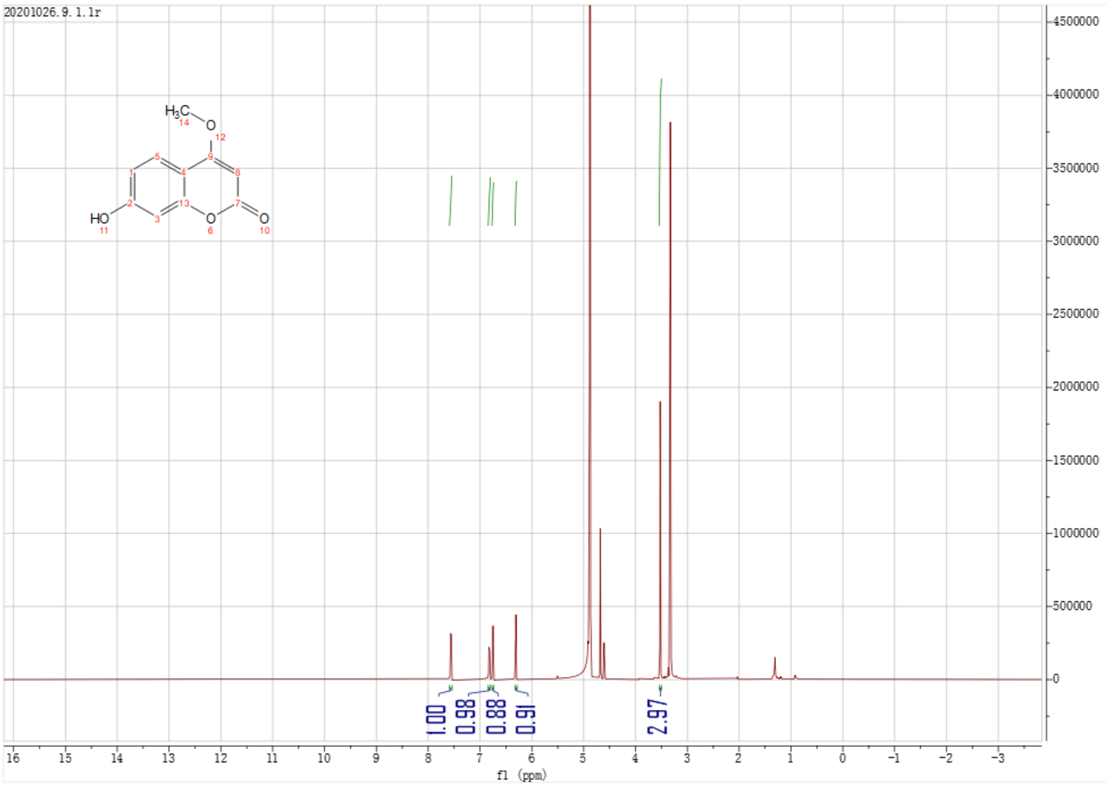

Figure S28. ^{1}H NMR of compound **10**.

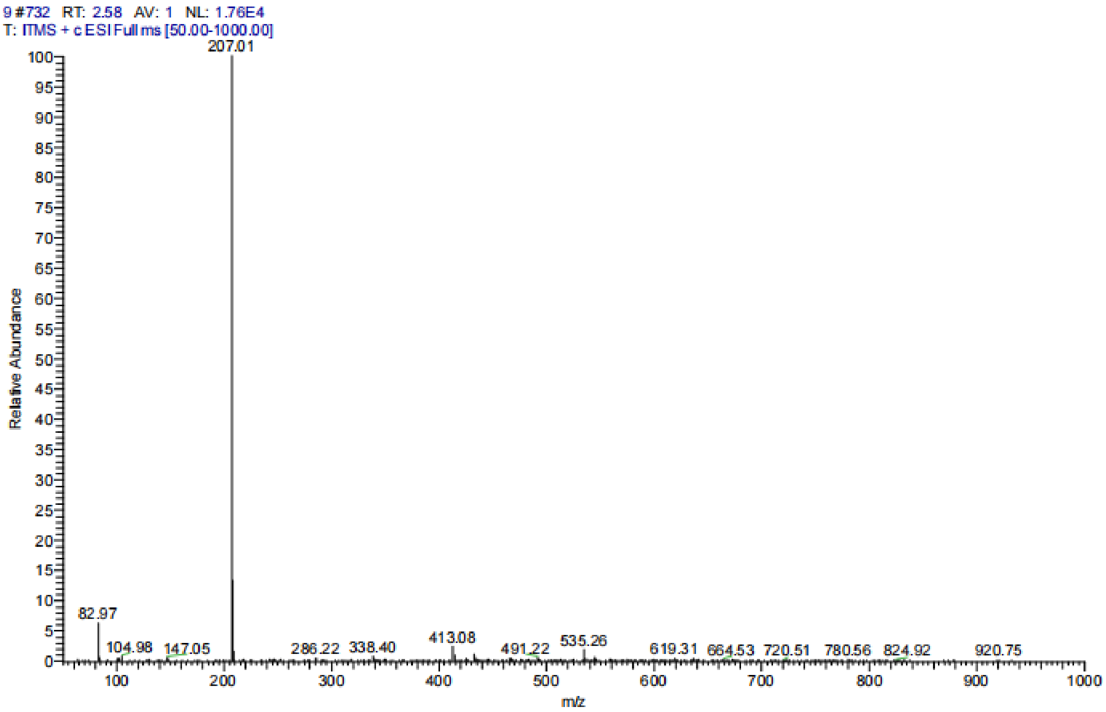

Figure S29. ESI-MS of compound **10**.

11. 3-Ethyl-7-hydroxy-4-methyl-2H-chromen-2-one (compound 11).

White amorphous powder, yield: 62%. ^{1}H NMR (600 MHz, CD_3OD) δ: 7.49 (1H, d, *J* = 8.8 Hz), 6.70 (1H, dd, *J* = 8.8, 2.4 Hz), 6.57 (1H, d, *J* = 2.4 Hz), 2.54 (2H, q, *J* = 7.5 Hz), 2.31 (3H, s), 1.01 (3H, t, *J* = 7.5 Hz). ^{13}C NMR (125 MHz, $CDCl_3$) δ: 167.2, 164.0, 157.5, 151.5,

129.7, 127.5, 117.4, 117.1, 106.4, 24.5, 18.3, 16.8. ESI-MS: *m/z* 205 [M+H] +; calcd for $C_{12}H_{12}O_3$, 204.23.**Error! Reference source not found.**

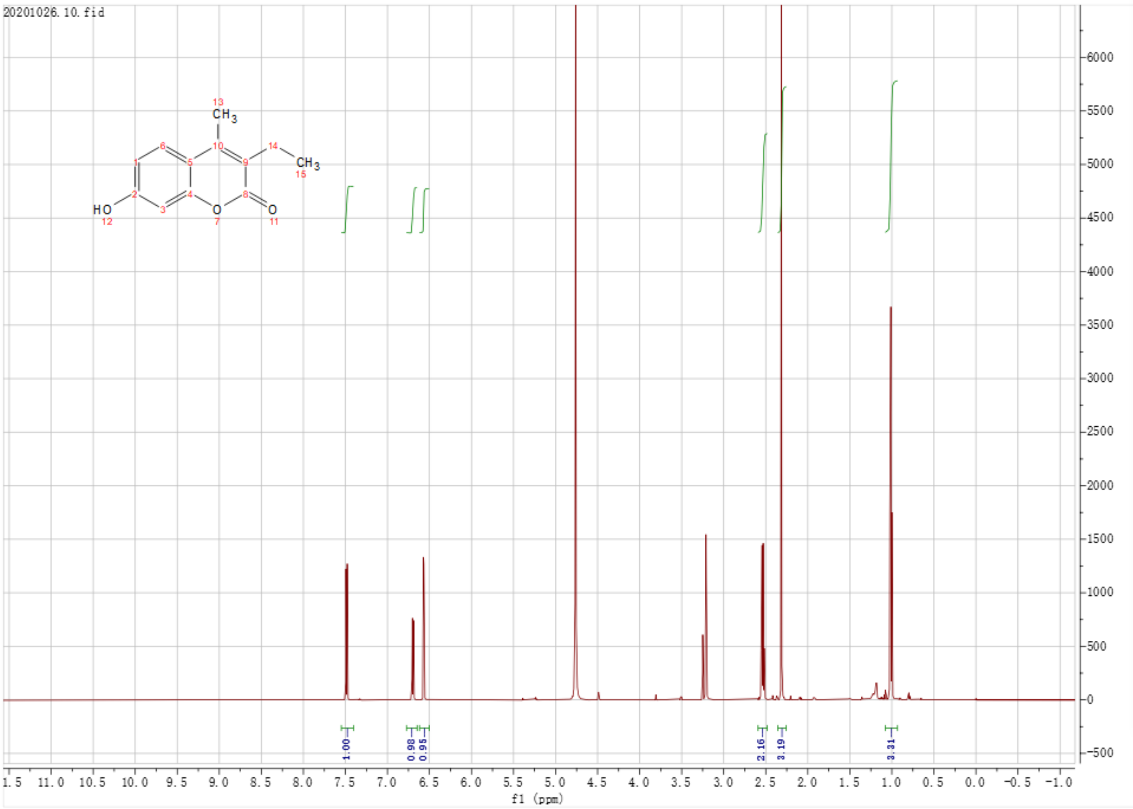

Figure S30. ^{1}H NMR of compound **11**.

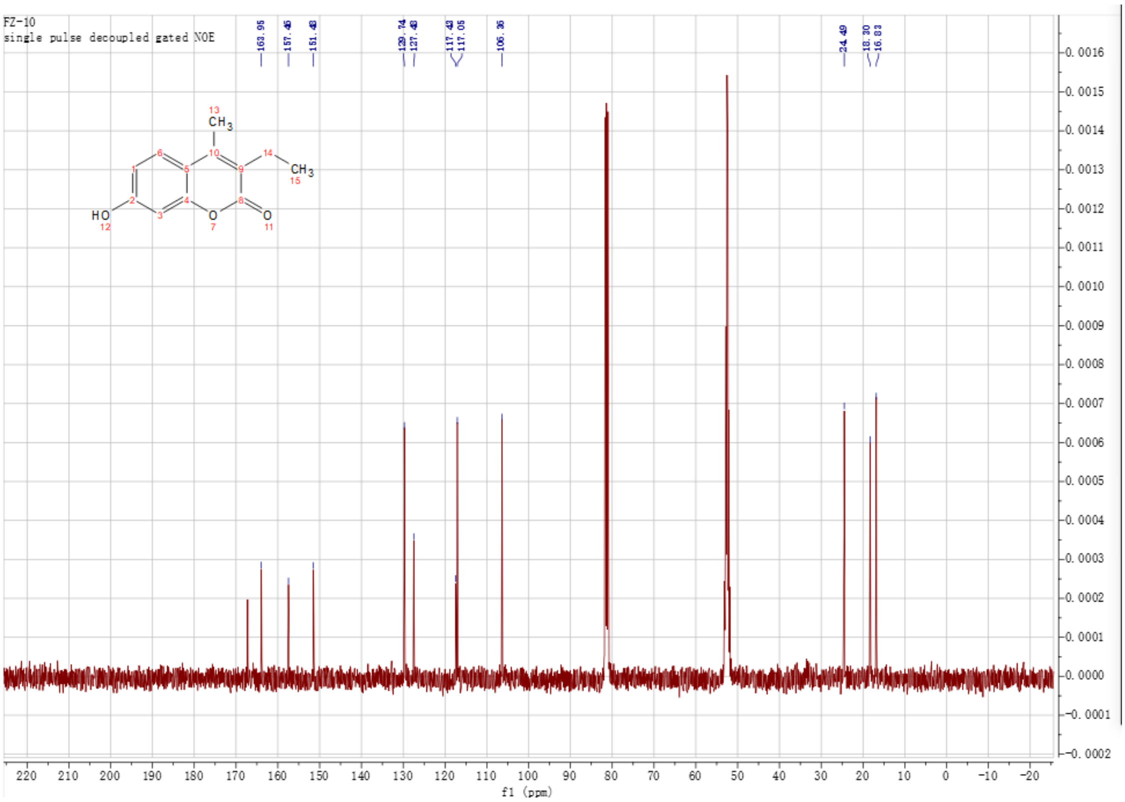

Figure S31. ^{13}C NMR of compound **11**.

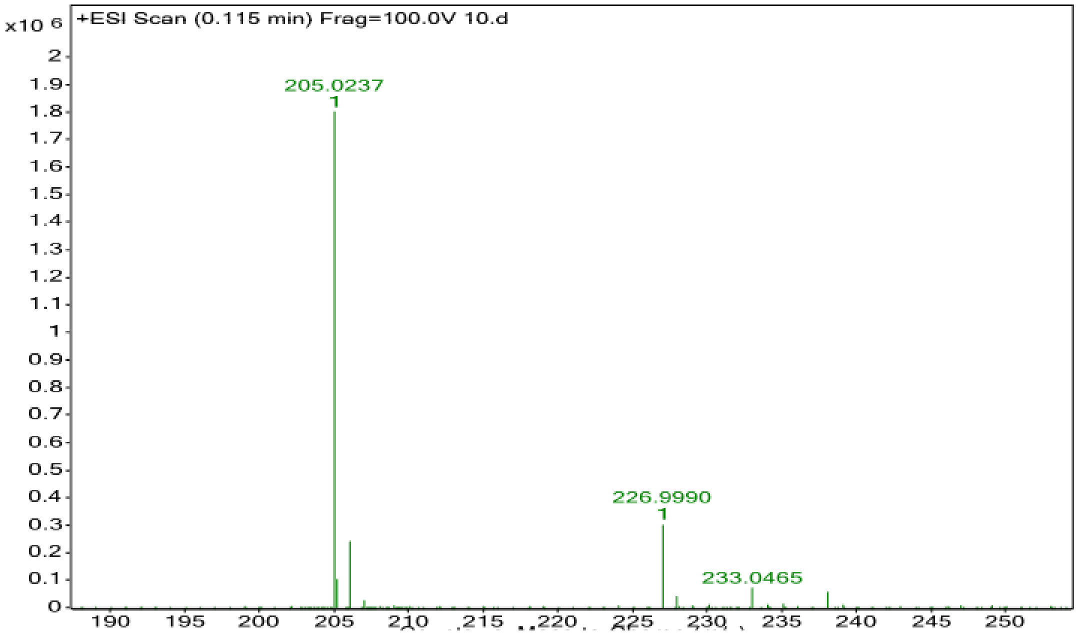

Figure S32. ESI-MS of compound **11**.

12. 7-Hydroxy-3, 4-dimethyl-2H-chromen-2-one (compound 12).

White amorphous powder, yield: 60%. ^{1}H NMR (600 MHz, CD_3OD) δ: 7.63 (1H, d, *J* = 8.8 Hz), 6.83 (1H, dd, *J* = 8.8, 2.4 Hz), 6.70 (1H, d, *J* = 2.4 Hz), 2.43 (3H, s), 2.16(3H, s). ^{13}C NMR (125 MHz, CD_3OD) δ: 163.7, 160.1, 153.5, 148.1, 125.7, 117.4, 113.3, 113.1, 102.3, 14.7, 12.5. ESI-MS: *m*/*z* 191 [M+H] [+]; calcd for $C_{11}H_{10}O_3$, 190.20.**Error! Reference source not found.**

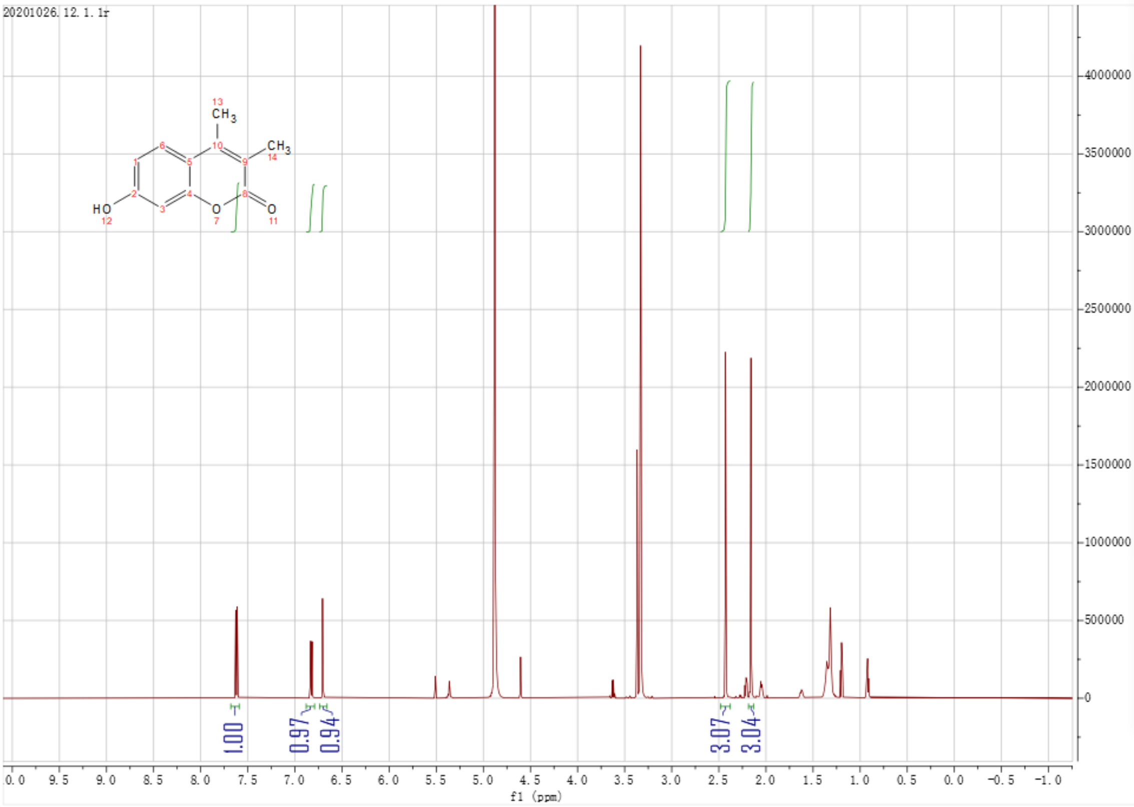

Figure S33. ^{1}H NMR of compound **12**.

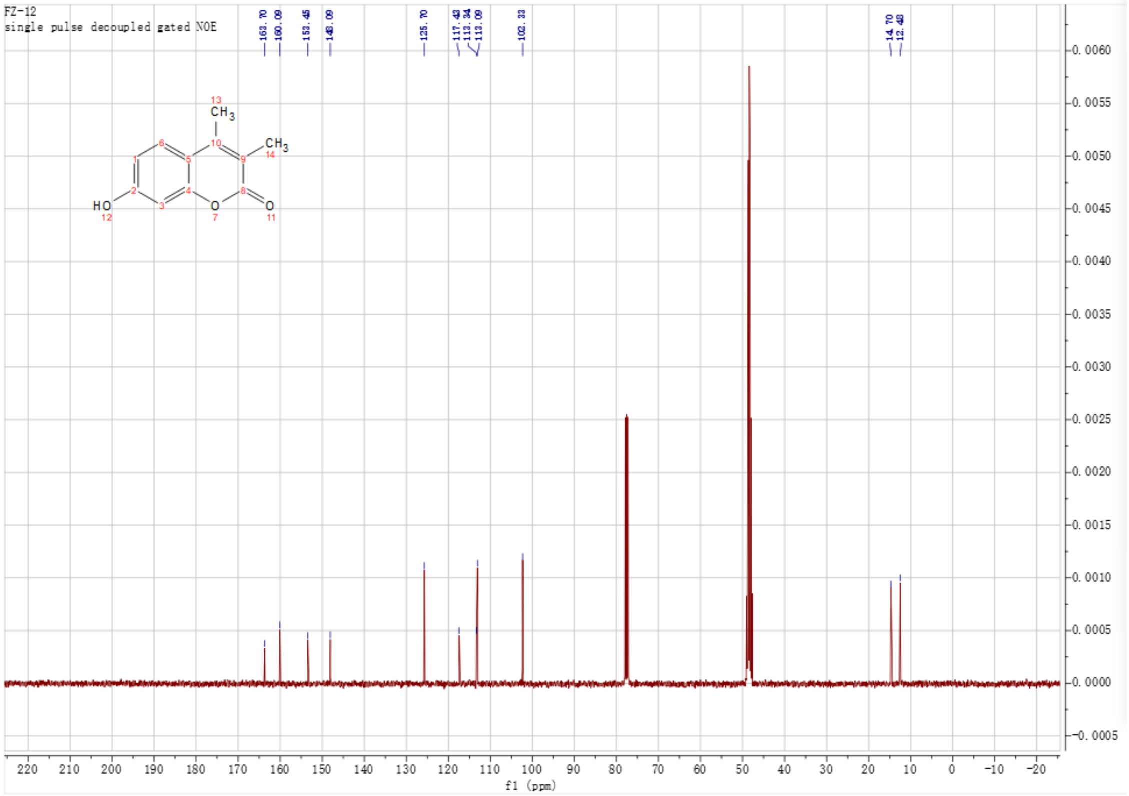

Figure S34. ^{13}C NMR of compound **12**.

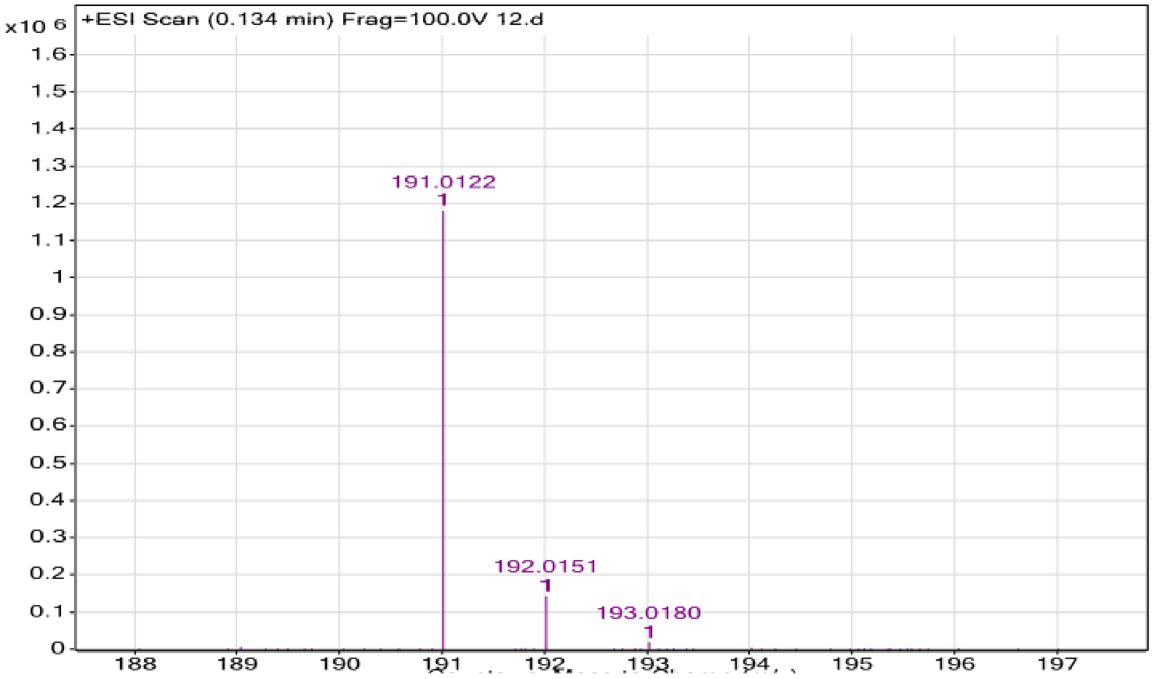

Figure S35. ESI-MS of compound **12**.

13. 7-Hydroxy-4-(trifluoromethyl)-2H-chromen-2-one (compound 13).

White amorphous powder, yield: 86%. ^{1}H NMR (400 MHz, $CDCl_3$) δ: 7.45 (1H, d, *J* = 7.6 Hz), 6.79–6.73 (1H, m), 6.73–6.69 (1H, m), 6.47–6.42 (1H, m). ^{13}C NMR (100 MHz, $CDCl_3$), δ: 162.7, 159.8, 156.5, 141.4, 126.2, 113.7, 111.1, 105.7, 104.3, 102.9. ESI-MS: *m*/*z* 231 $[M+H]^+$; calcd for $C_{10}H_5F_3O_3$, 230.14.**Error! Reference source not found.**

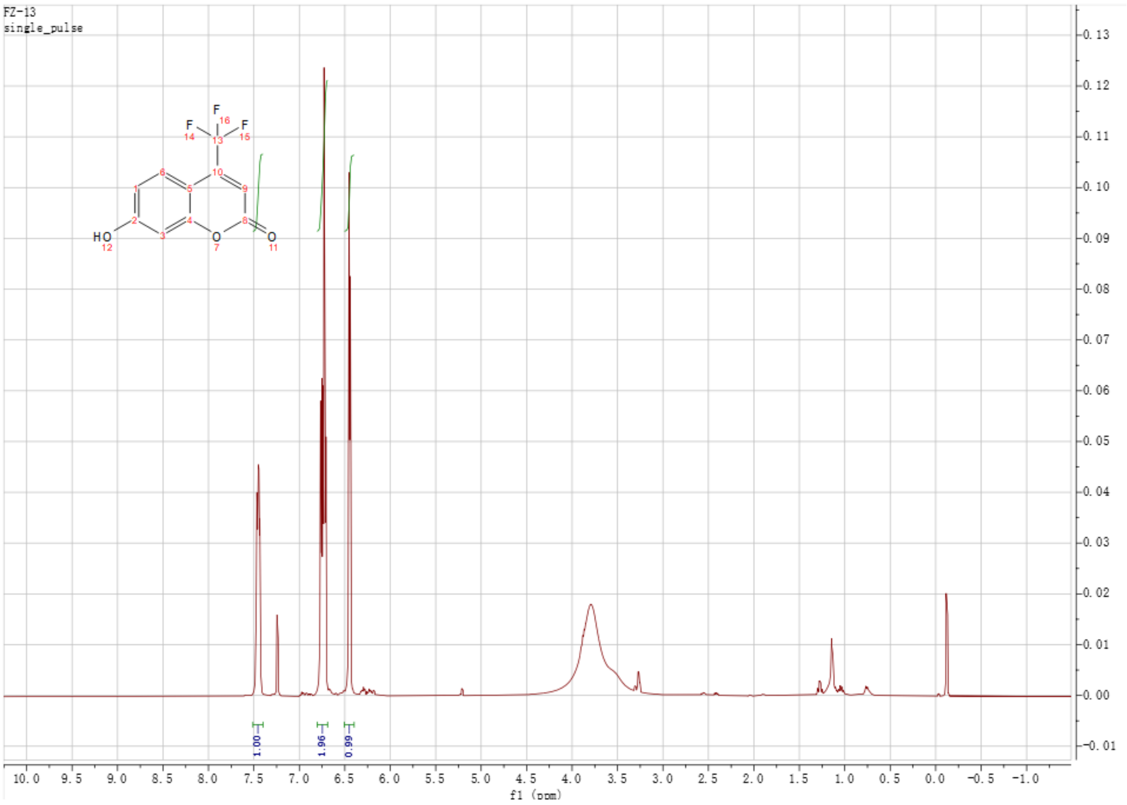

Figure S36. ^{1}H NMR of compound **13**.

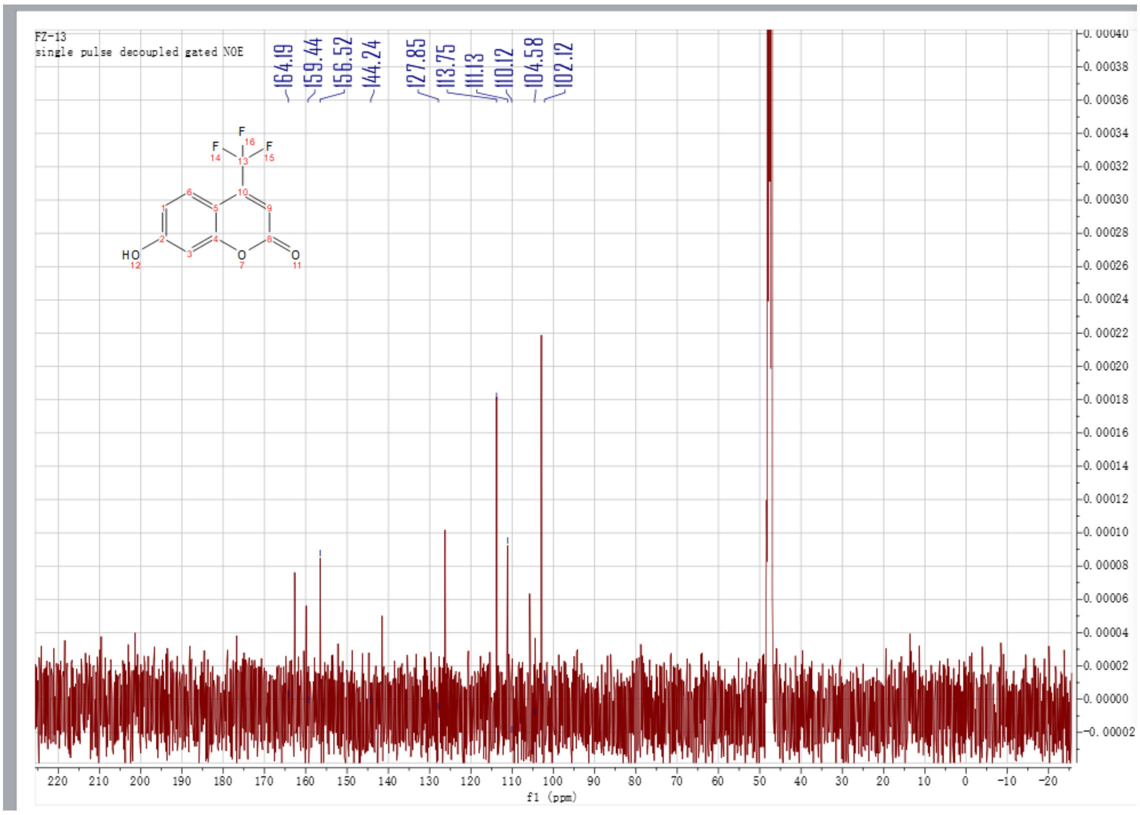

Figure S37. ^{13}C NMR of compound **13**.

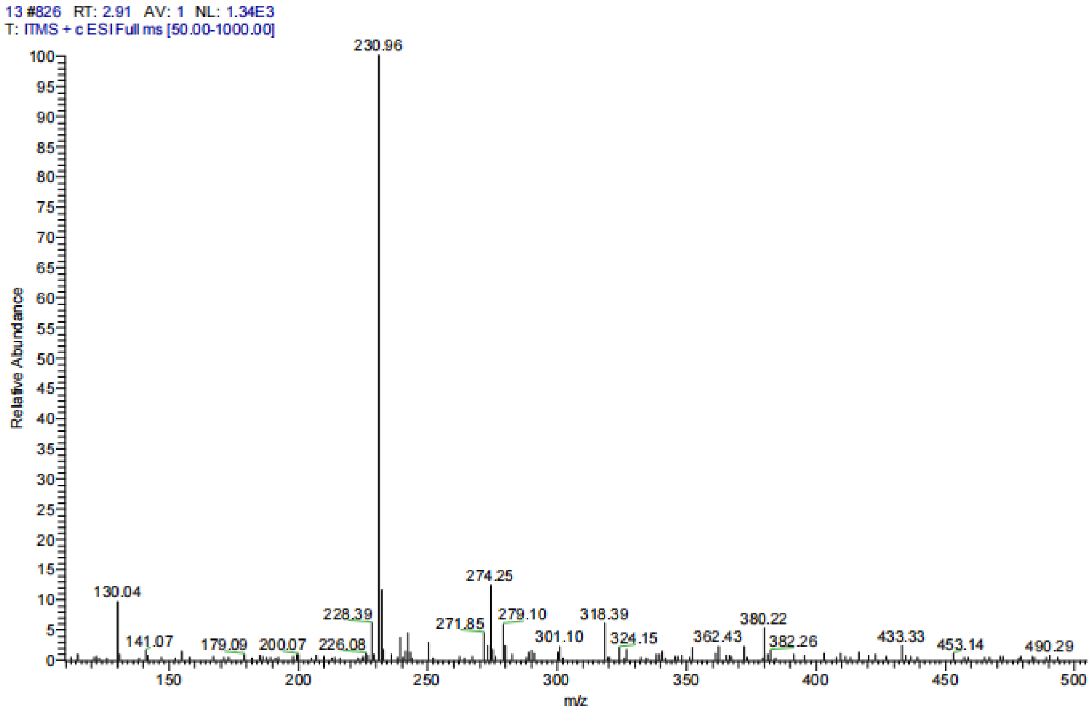

Figure S38. ESI-MS of compound **13**.

14. 3-Chloro-7-hydroxy-4-methyl-2H-chromen-2-one (compound 14).

White amorphous powder, yield: 20%. ^{1}H NMR (600 MHz, CD_3OD) δ: 7.67 (1H, d, *J* = 8.8 Hz), 6.89 (1H, dd, *J* = 8.8, 2.4 Hz), 6.76 (1H, d, *J* = 2.4 Hz), 2.59 (3H, s). ^{13}C NMR (100 MHz, CD_3OD) δ: 161.6, 158.3, 153.2, 149.5, 126.5, 123.9, 113.7, 112.3, 102.2, 15.2. ESI-MS: *m*/*z* 211 $[M+H]^+$; calcd for $C_{10}H_7ClO_3$, 210.61.

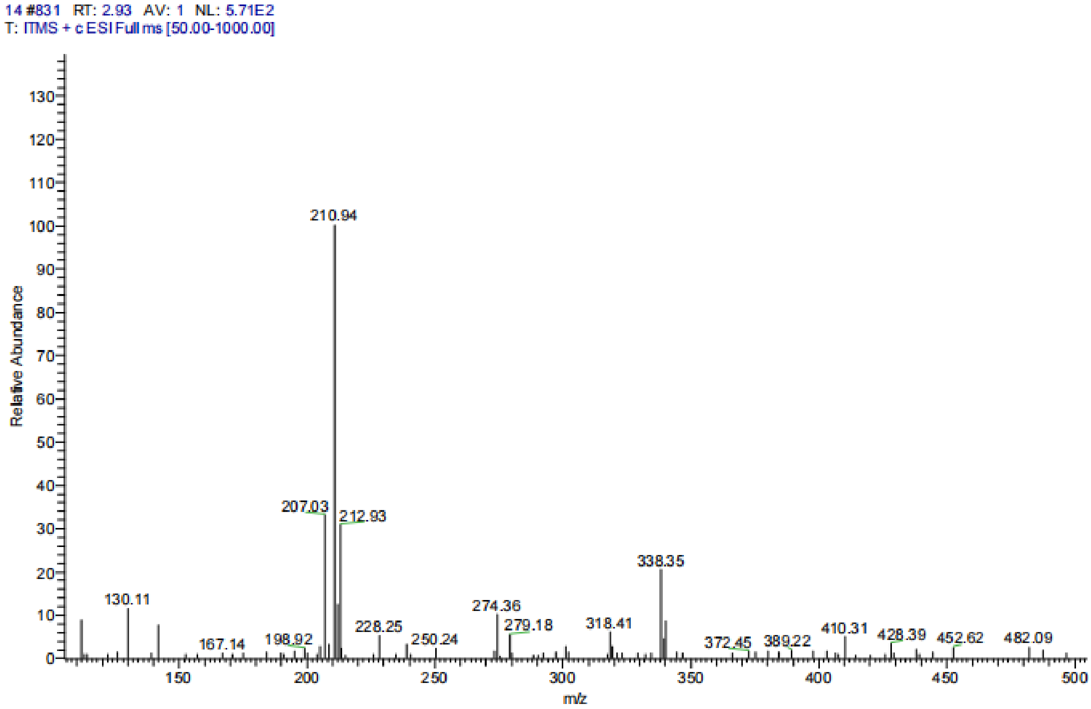

Figure S39. ESI-MS of compound **14**.

15. 3-Benzyl-7-hydroxy-4-methyl-2H-chromen-2-one (compound 15).

White amorphous powder, yield: 83%. ^{1}H NMR (400 MHz, CD_3OD) δ 7.57 (1H, d, *J* = 8.8 Hz), 7.24-7.07 (5H, m), 6.78 (1H, dd, *J* = 8.8, 2.4 Hz), 6.68 (1H, d, *J* = 2.4 Hz), 3.97 (2H, s), 2.39 (3H, s). ^{13}C NMR (100 MHz, CD_3OD) δ: 163.3, 160.8, 153.9, 149.6, 139.4, 128.2 (2C), 127.9 (2C), 126.2, 125.9 120.5, 113.1 (2C), 102.0, 32.1, 14.4. ESI-MS: *m*/*z* 267 [M+H] $^+$; calcd for $C_{17}H_{14}O_3$, 266.30.

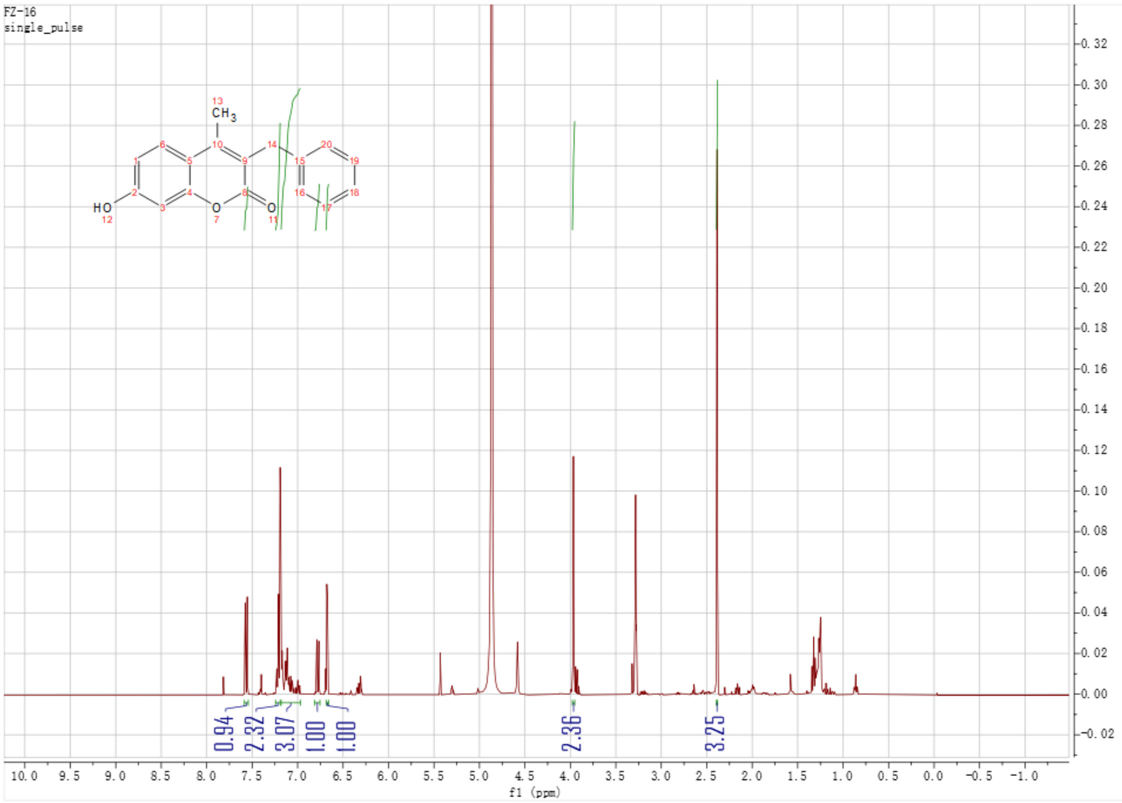

Figure S40. ^{1}H NMR of compound **15**.

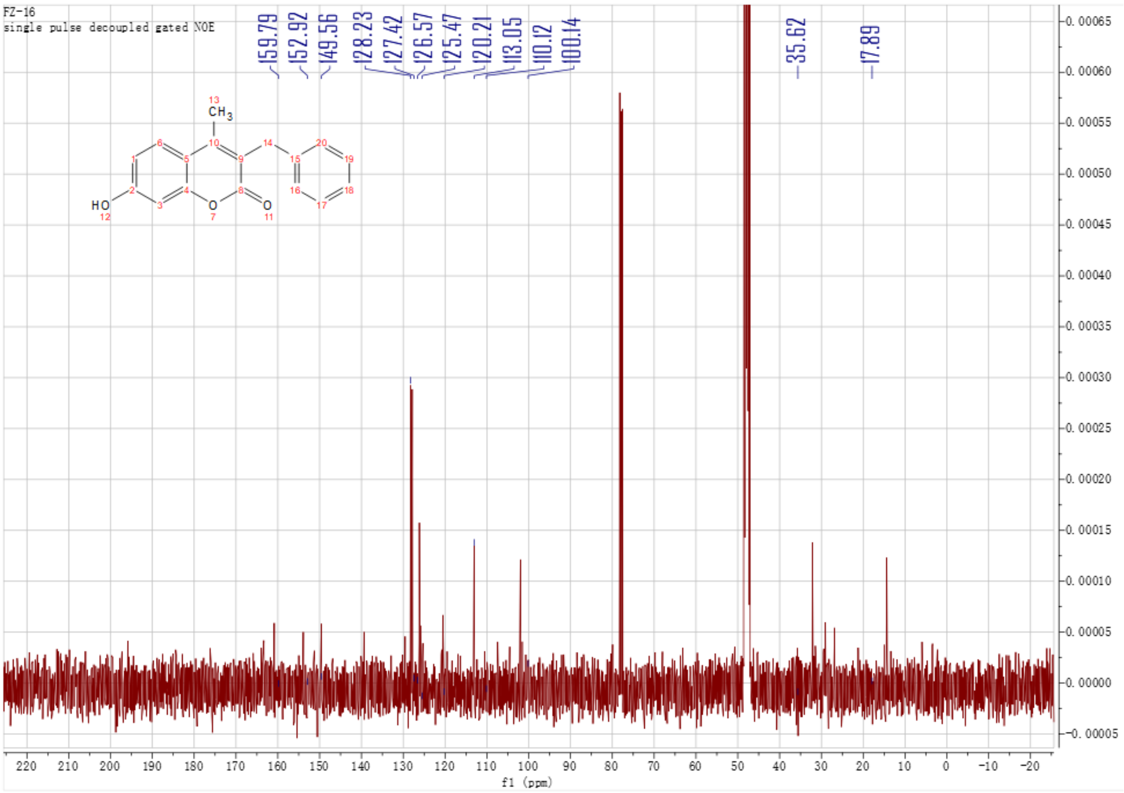

Figure S41. ^{13}C NMR of compound **15**.

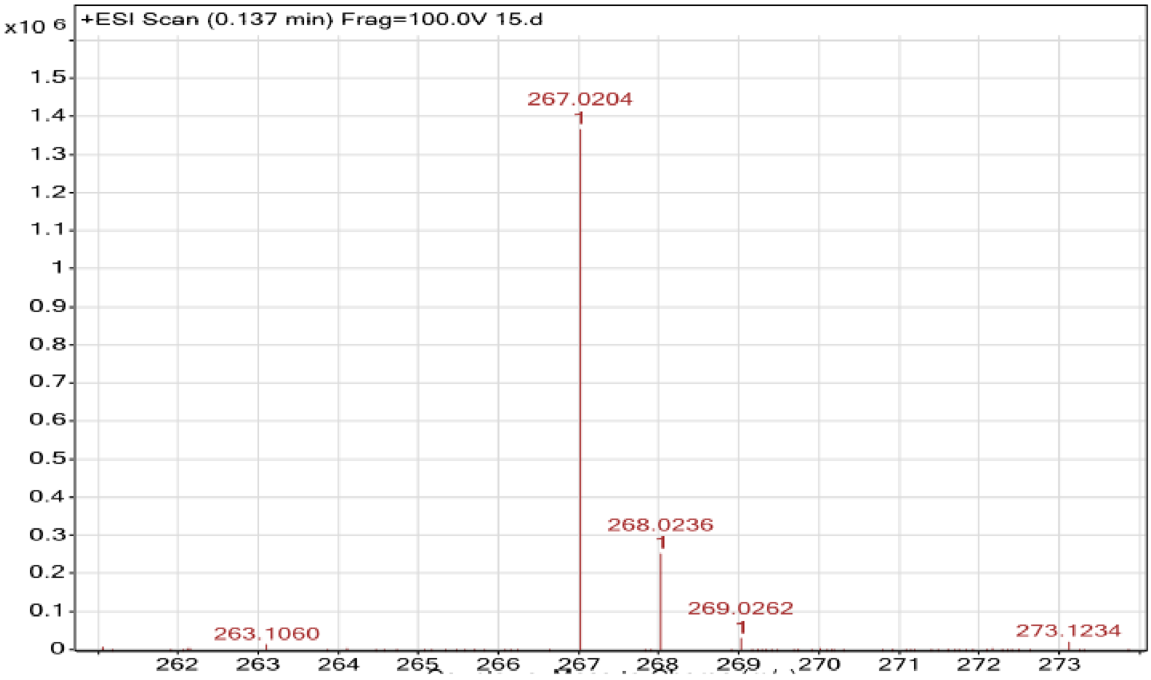

Figure S42. ESI-MS of compound **15**.

16. 3-Fluoro-7-hydroxy-4-methyl-2H-chromen-2-one (compound 16).

White amorphous powder, yield: 38%. ^{1}H NMR (400 MHz, CD_3OD) δ: 7.53 (1H, d, *J* = 8.8 Hz), 6.84 (1H, dd, *J* = 8.8, 2.4 Hz), 6.70 (1H, d, *J* = 2.4 Hz), 2.35 (3H, d, *J* = 2.8 Hz). ^{13}C NMR (100 MHz, CD_3OD) δ: 160.6, 156.0, 151.8, 140.5, 132.4, 126.0, 113.6, 111.5, 102.2, 8.9. ESI-MS: *m*/*z* 195 $[M+H]^+$; calcd for $C_{10}H_7FO_3$, 194.16.**Error! Reference source not found.**

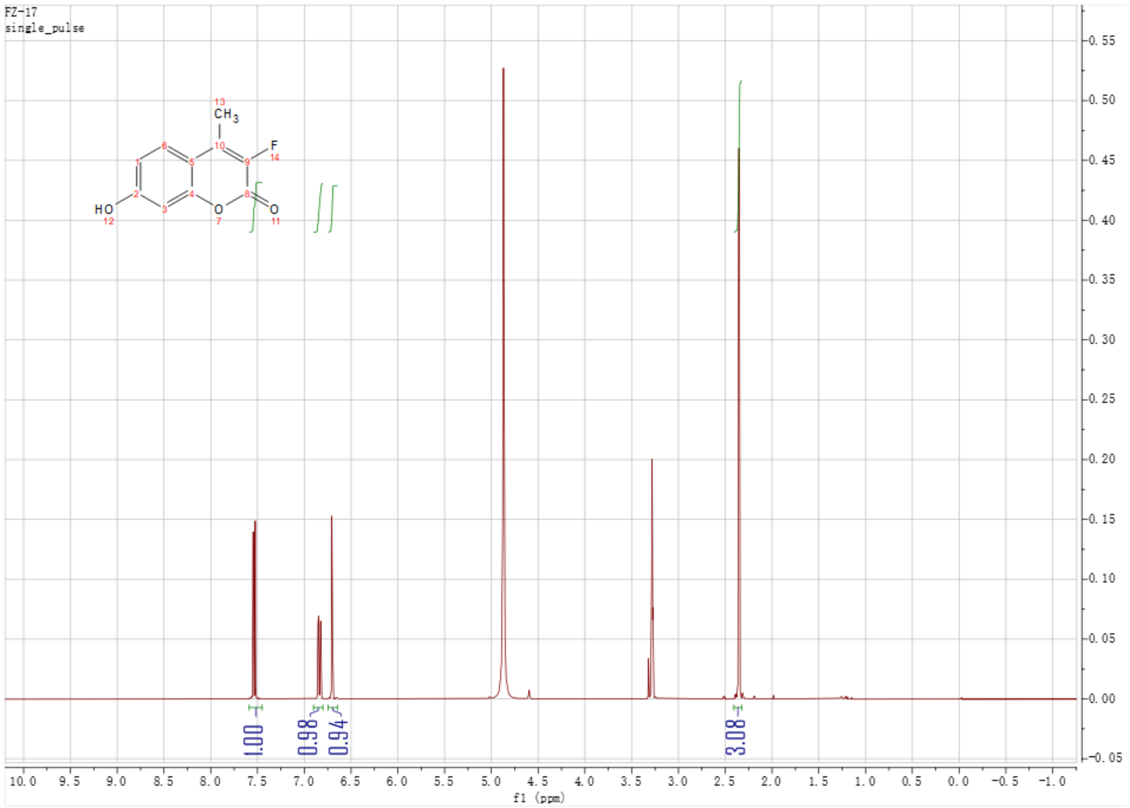

Figure S43. ^{1}H NMR of compound **16**.

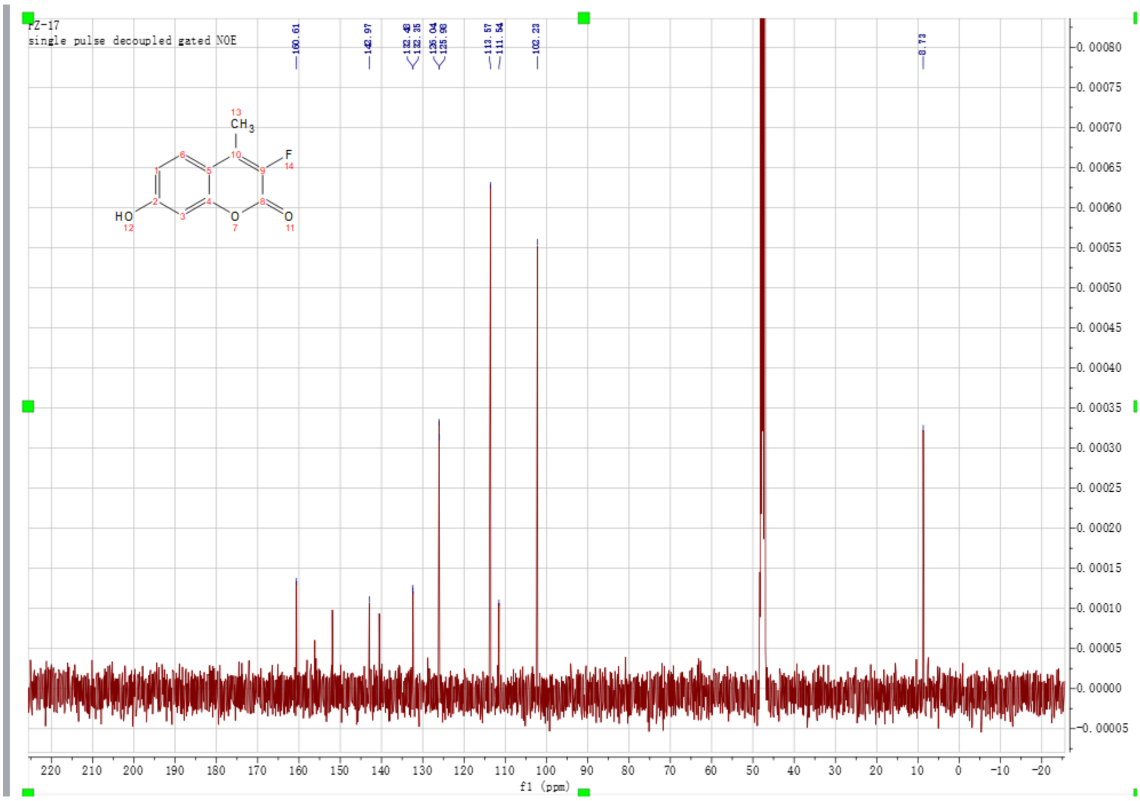

Figure S44. ^{13}C NMR of compound **16**.

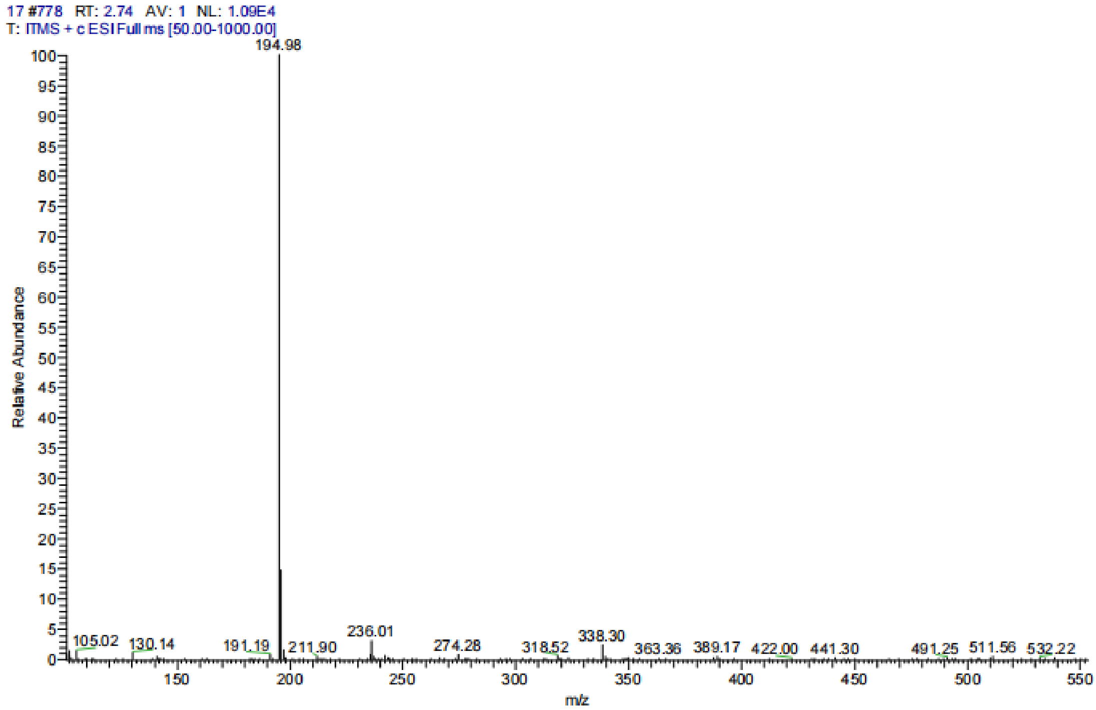

Figure S45. ESI-MS of compound **16**.

17. 7-Hydroxy-4-methyl-2-oxo-2H-chromene-3-carbonitrile (compound 17).

White amorphous powder, yield: 16%. ^1H NMR (400 MHz, $CDCl_3$) δ*:* 7.86 (1H, d, *J* = 8.9 Hz), 6.96 (1H, dd, *J* = 8.9, 2.7 Hz), 6.84 (1H, d, *J* = 2.7 Hz) 2.73 (3H s). ^{13}C NMR (101 MHz, CD_3OD) δ*:* 161.56, 157.82, 145.3, 129.9, 129.8, 129.5, 113.7, 111.1, 106.9, 102.5, 13.4. ESI-MS: *m*/*z* 201 $[M+H]^+$; calcd for $C_{11}H_7NO_3$, 203.77.

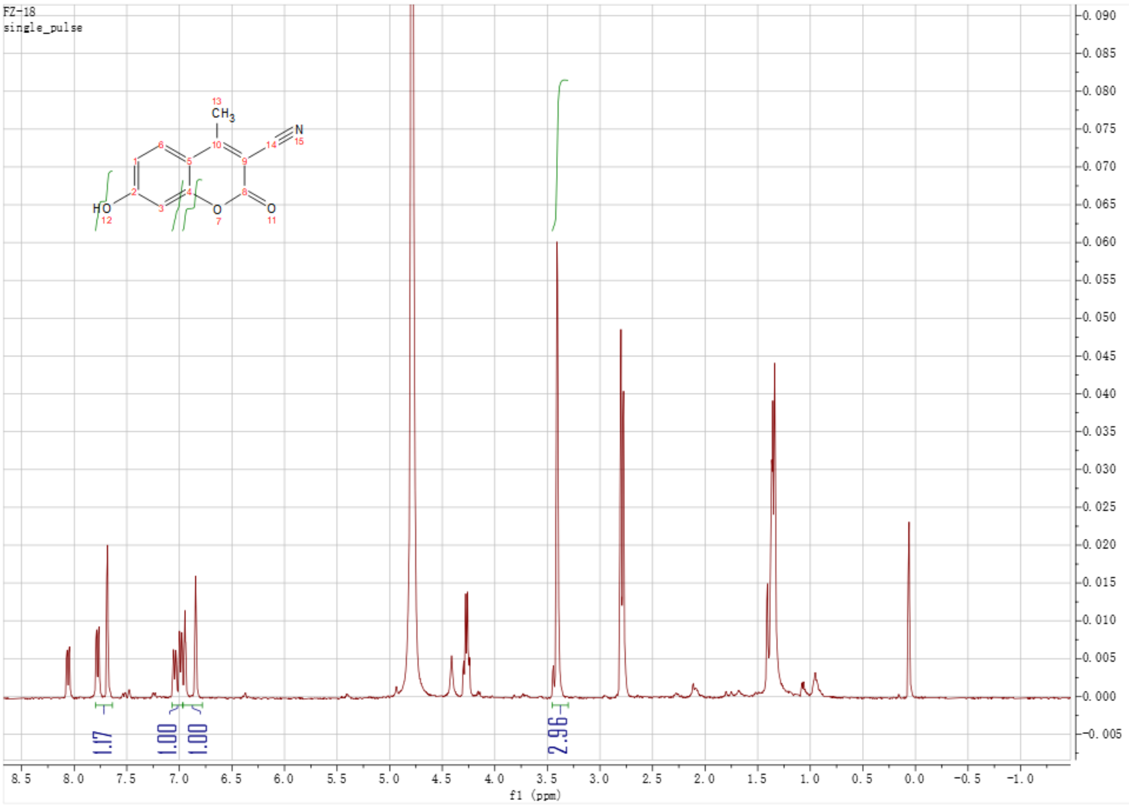

Figure S46. ^1H NMR of compound **17**.

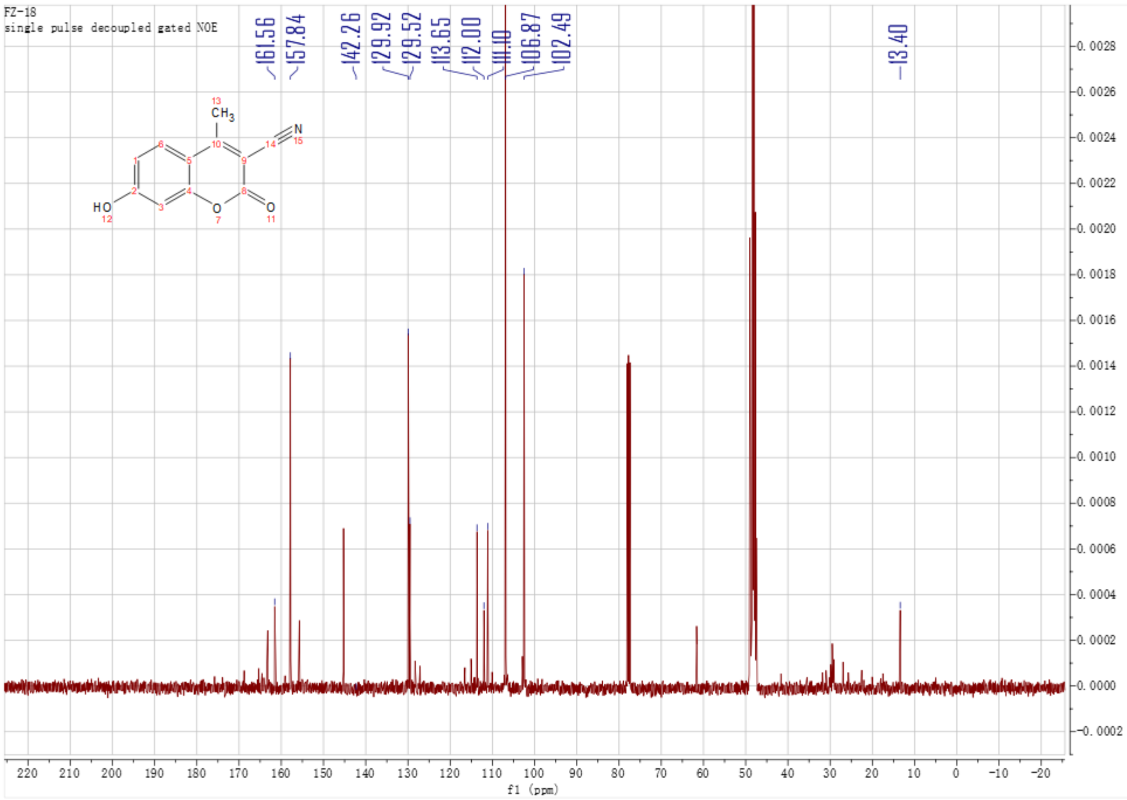

Figure S47. ^{13}C NMR of compound **17**.

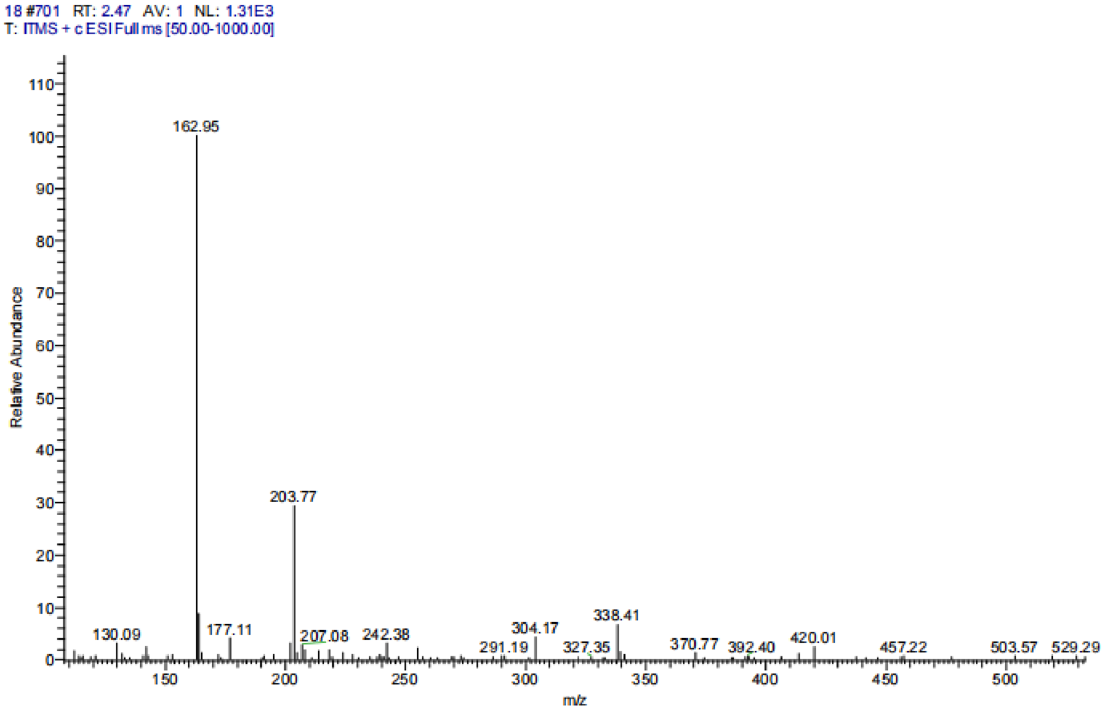

Figure S48. ESI-MS of compound **17**.

18. 4-Ethyl-7-hydroxy-2H-chromen-2-one (compound 18).

White amorphous powder, yield: 48%. ^{1}H NMR (400 MHz, CD_3OD) δ: 7.50 (1H, d, *J* = 8.8 Hz), 6.82 (2H, dd, *J* = 8.8, 2.4 Hz), 6.11 (1H, d, *J* = 8.8 Hz), 2.82 (2H, q, *J* =8.8Hz), 1.33 (3H, dd, *J* = 2.4 Hz). ^{13}C NMR (100 MHz, CD_3OD), δ: 167.2, 165.0, 163.3, 159.2, 129.5, 117.3, 115.9, 112.4, 106.9, 28.7, 16.1. ESI-MS: *m*/*z* 191 [M+H] $^{+}$; calcd for $C_{11}H_{10}O_3$, 190.20.

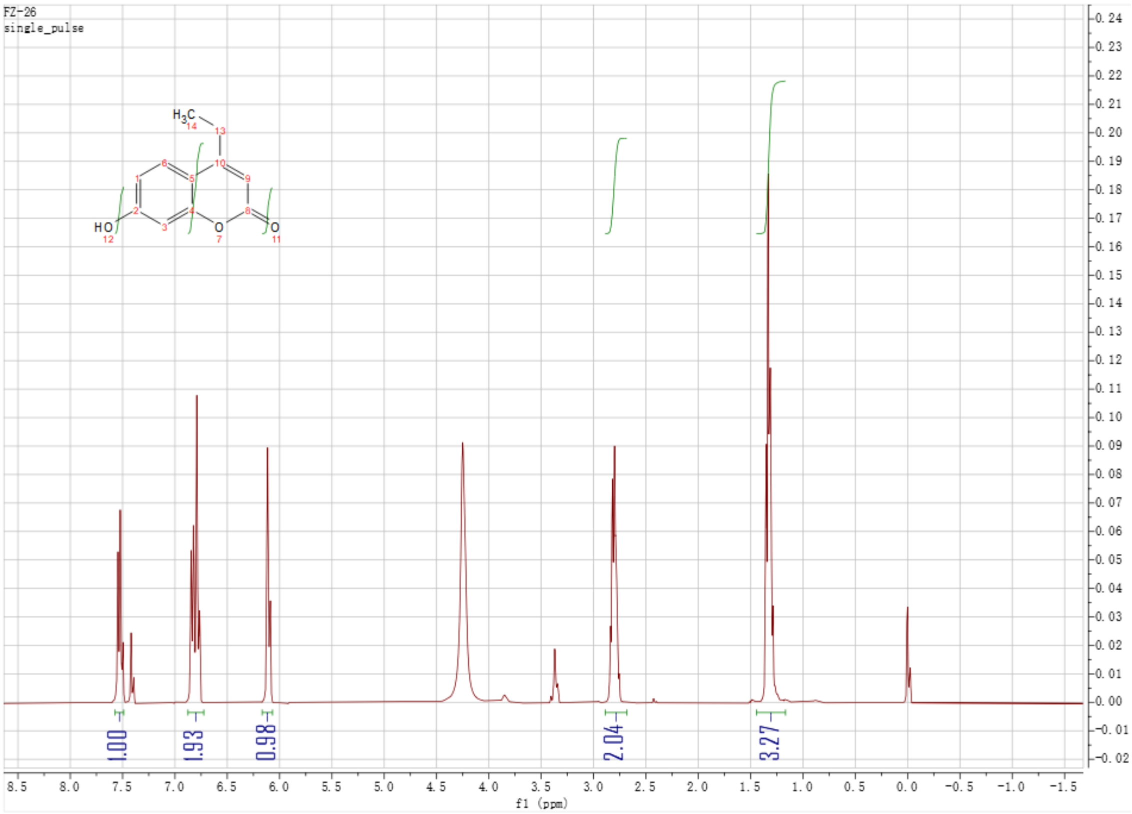

Figure S49. ¹H NMR of compound **18**.

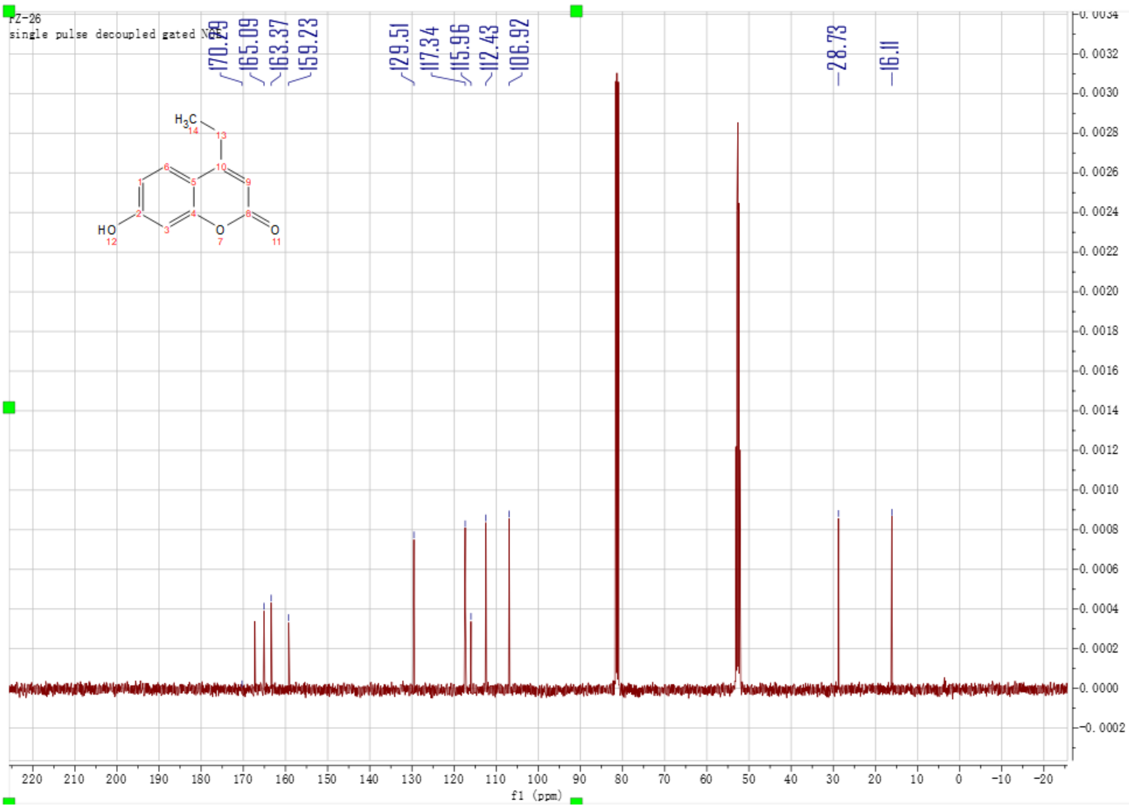

Figure S50. ¹³C NMR of compound **18**.

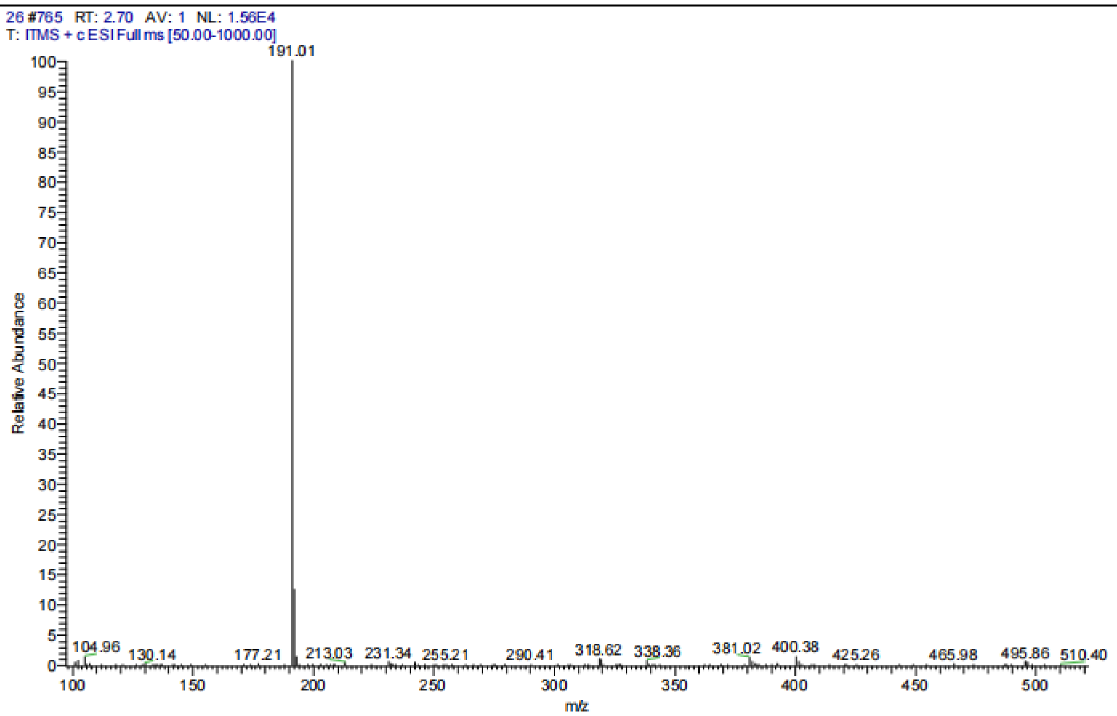

Figure S51. ESI-MS of compound **18**.

19. 4-(Chloromethyl)-7-hydroxy-2H-chromen-2-one (compound 19).

White amorphous powder, yield: 51%. ^{1}H NMR (400 MHz, CD_3OD) δ: 7.56 (1H, d, *J* = 8.8 Hz), 6.89 (1H, dd, *J* = 8.8, 2.4 Hz), 6.80 (1H, d, *J* = 8.8 Hz), 6.38 (1H, d, *J* = 2.4 Hz), 4.68 (2H, s). ^{13}C NMR (100 MHz, CD_3OD), δ: 166.2, 165.6, 159.5, 155.0, 129.5, 117.6, 115.0, 113.9, 107.1, 45.1. ESI-MS: *m*/*z* 211 [M+H] $^+$; calcd for $C_{10}H_7ClO_3$, 210.61.**Error! Reference source not found.**

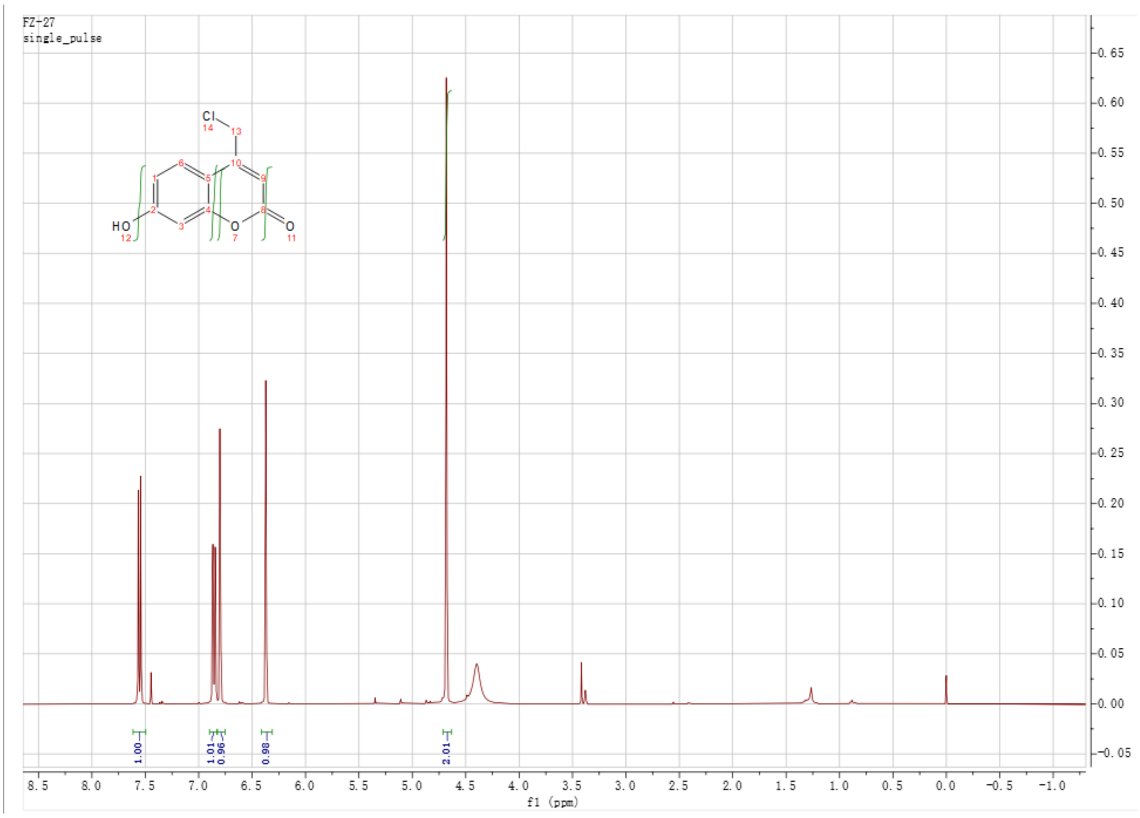

Figure S52. ^{1}H NMR of compound **19**.

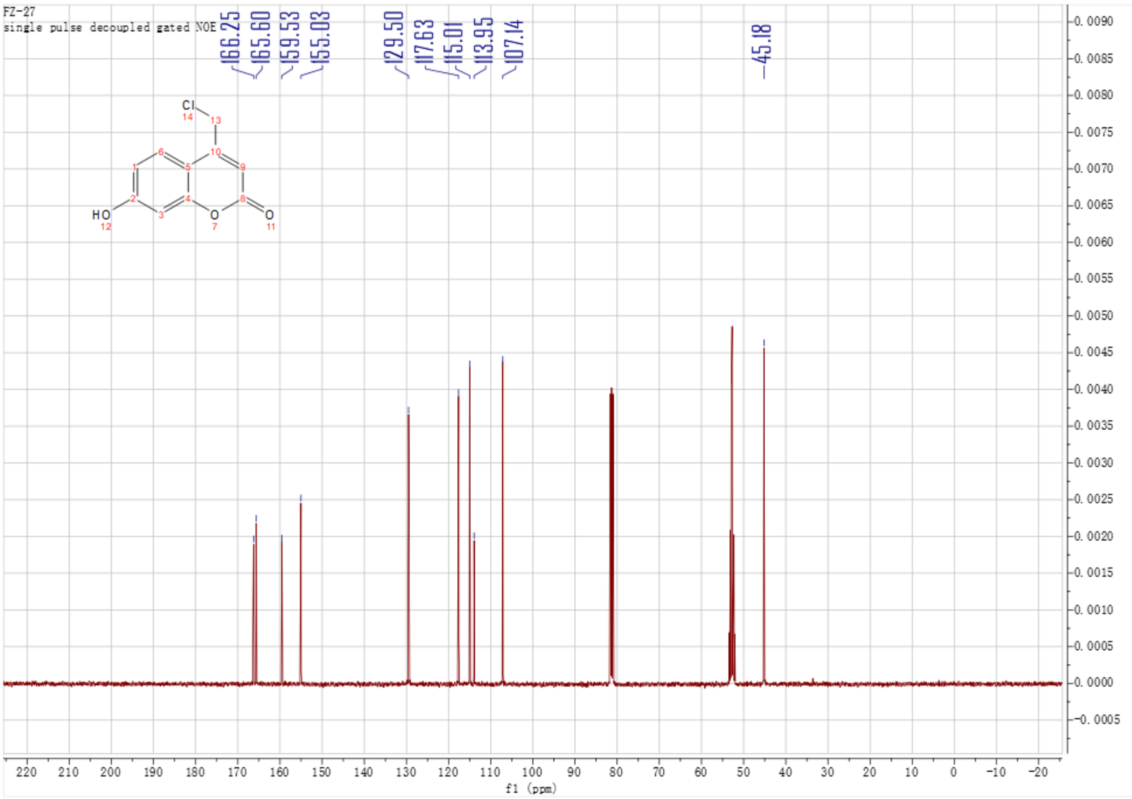

Figure S53. ^{13}C NMR of compound **19**.

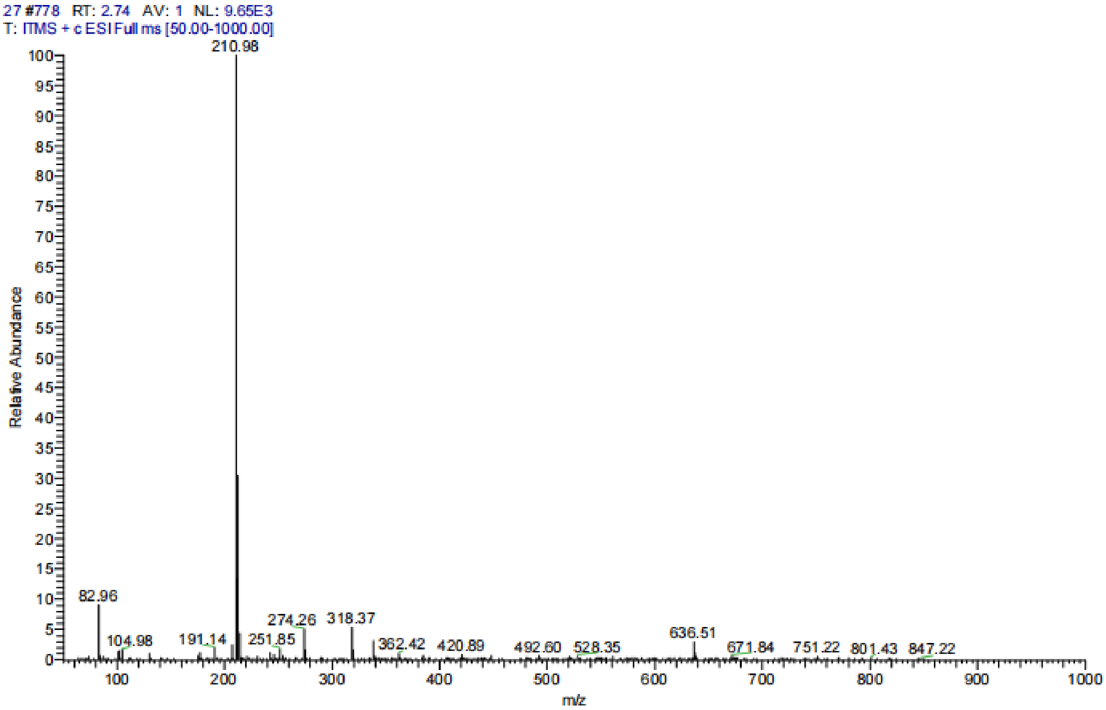

Figure S54. ESI-MS of compound **19**.

20. 7-Hydroxy-4-phenyl-2H-chromen-2-one (compound 20).

White amorphous powder, yield: 58%. ^{1}H NMR (400 MHz, CD_3OD) δ: 7.55–7.42 (5H, m), 7.31 (1H, d, *J* = 8.8 Hz), 6.77 (1H, d, *J* = 2.4 Hz), 6.73 (1H, dd, *J* = 8.7, 2.4 Hz), 6.11 (1H,

s). ^{13}C NMR (100 MHz, CD_3OD) δ: 166.18, 165.92, 161.08, 159.99, 139.65, 133.39, 132.57(2 C), 132.16(2 C), 131.27, 117.04, 115.28, 113.92, 106.47. ESI-MS: *m*/*z* 238 $[M+H]^+$; calcd for $C_{15}H_{10}O_3$, 239.02.**Error! Reference source not found.**

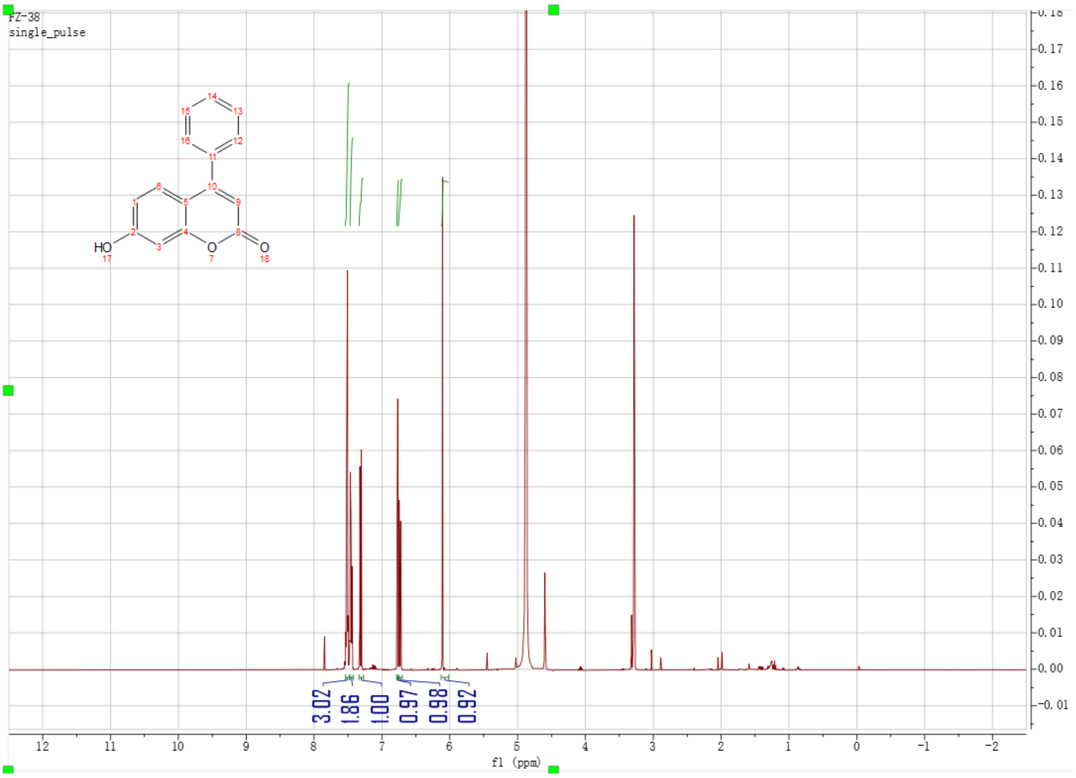

Figure S55. ^1H NMR of compound **20**.

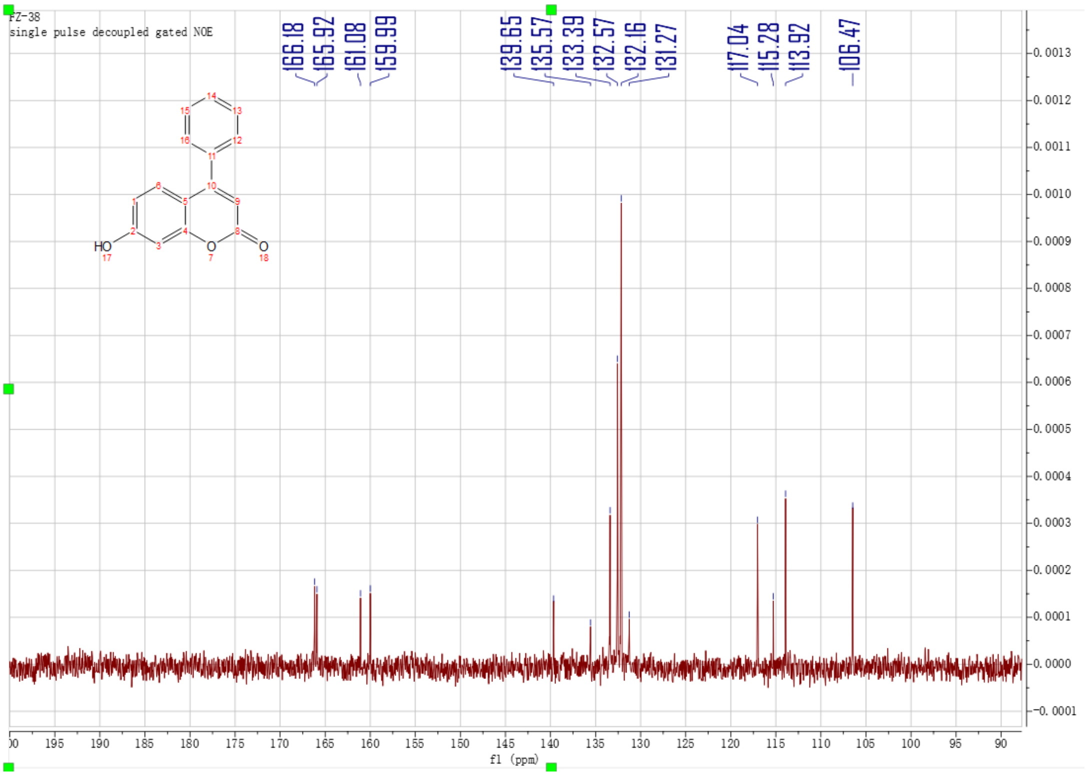

Figure S56. ^{13}C NMR of compound **20**.

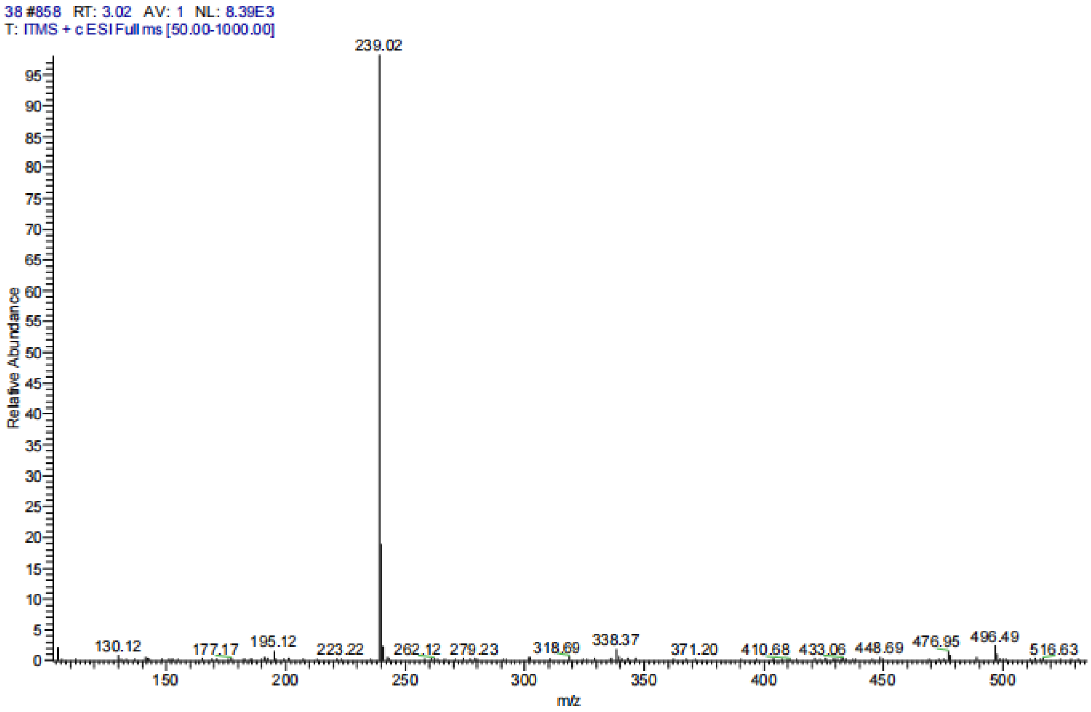

Figure S57. ESI-MS of compound **20**.

21.7–((2–. chloropyrimidin–4–yl)oxy)–4–methyl–2H–chromen–2–one (compound 21).

White amorphous powder, yield: 49%. ^{1}H NMR (600 MHz, $CDCl_3$) δ: 8.52 (1H, d, *J* = 5.6 Hz), 7.68(1H, d, *J* = 8.7 Hz), 7.20 (1H, d, *J* = 2.3 Hz), 7.15 (1H, dd, *J* = 8.7, 2.3 Hz), 6.93 (1H, d, *J* = 5.6 Hz), 6.31 (1H, d, *J* = 1.2 Hz), 2.47 (3H, d, *J* = 1.2 Hz). ^{13}C NMR (126 MHz, $CDCl_3$) δ: 169.45, 160.68, 160.49, 160.44, 154.42, 154.00, 151.96, 125.86, 118.15, 117.77, 114.69, 110.22, 107.33, 18.81. ESI-MS: *m*/*z* 289 [M+H] $^{+}$; calcd for $C_{14}H_9ClN_2O_3$, 288.5.

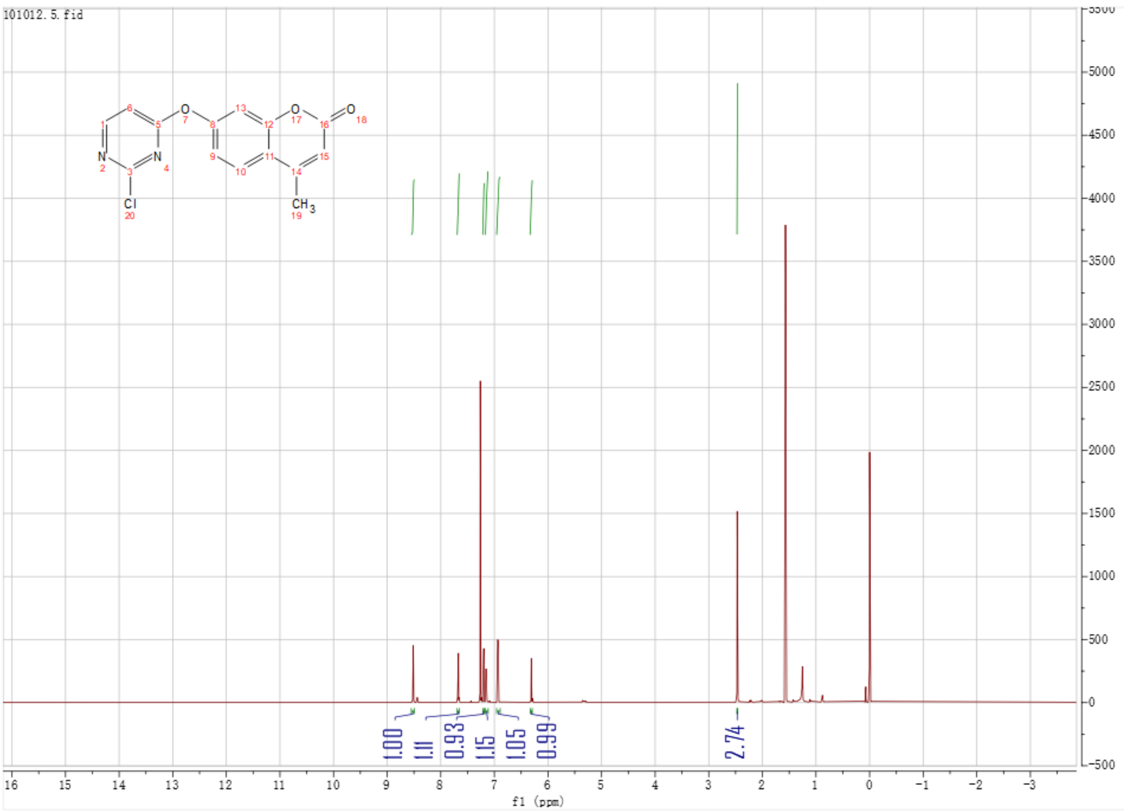

Figure S58. ^{1}H NMR of compound **21**.

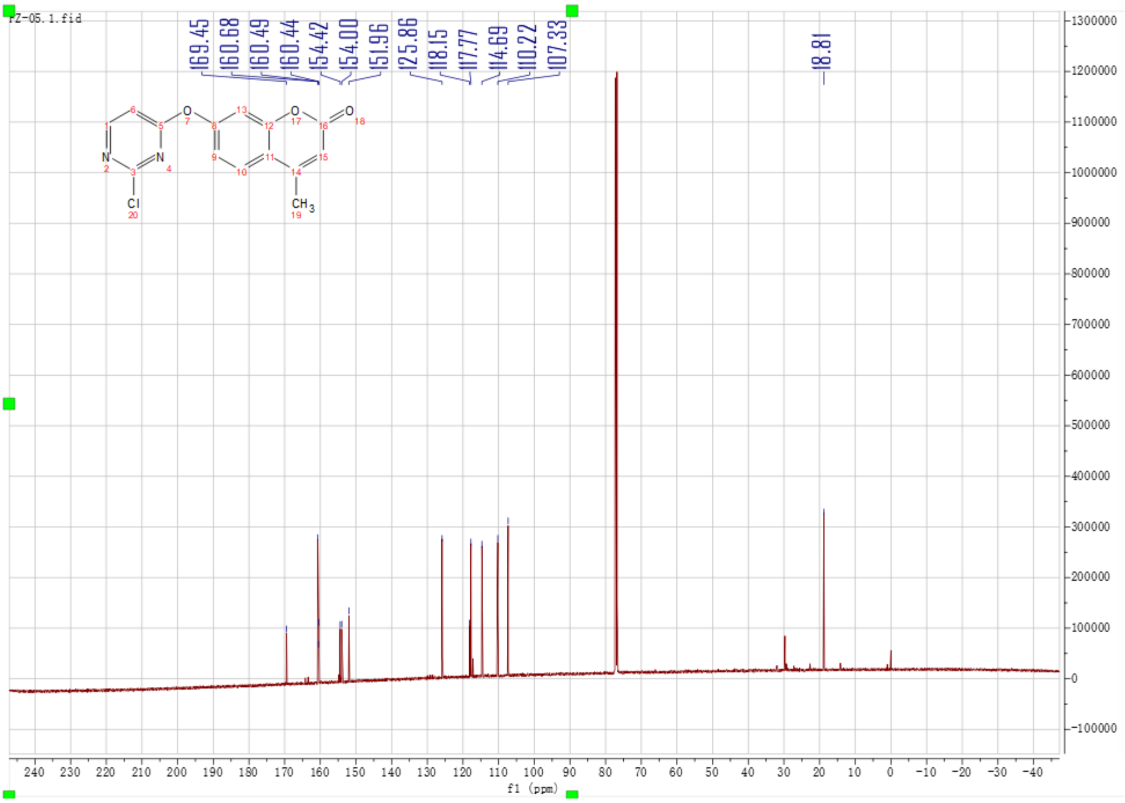

Figure S59. ^{13}C NMR of compound **21**.

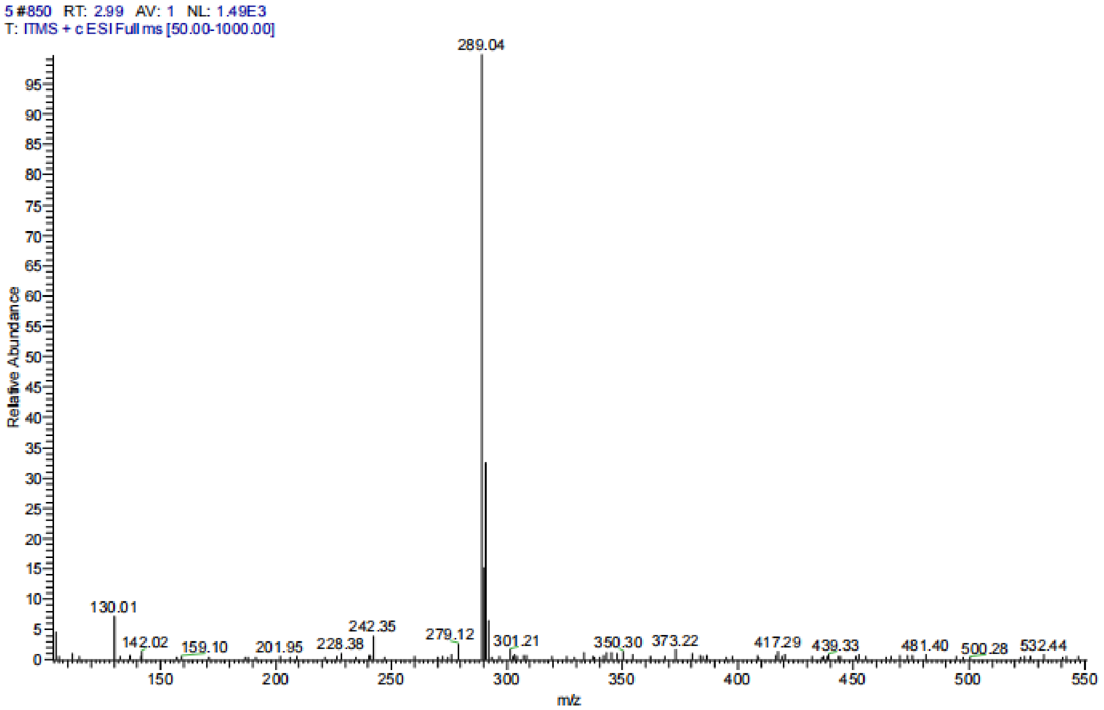

Figure S60. ESI-MS of compound **21**.

22. 3-Benzyl-7-methoxy-4-methyl-2H-chromen-2-one (compound 22).

White amorphous powder, yield: 7%. ^{1}H NMR (400 MHz, $CDCl_3$) δ: 7.48 (1H, d, *J* = 8.9 Hz), 7.28-7.21 (4H, m), 7.20-7.11 (1H, m), 6.83 (1H, dd, *J* = 8.8, 2.6 Hz), 6.78 (1H, d, *J* =

2.6 Hz), 4.02 (2H, s), 2.39 (3H, s). ^{13}C NMR (101 MHz, CD_3Cl) δ: 162.3 162.2, 153.9, 148.0, 139.4, 128.6 (2C), 128.4 (2C), 126.4, 125.8, 122.0, 114.1, 112.2, 100.6, 55.8, 32.9, 15.5. ESI-MS: *m/z* 281 $[M+H]^+$; calcd for $C_{18}H_{16}O_3$, 280.32.**Error! Reference source not found.**

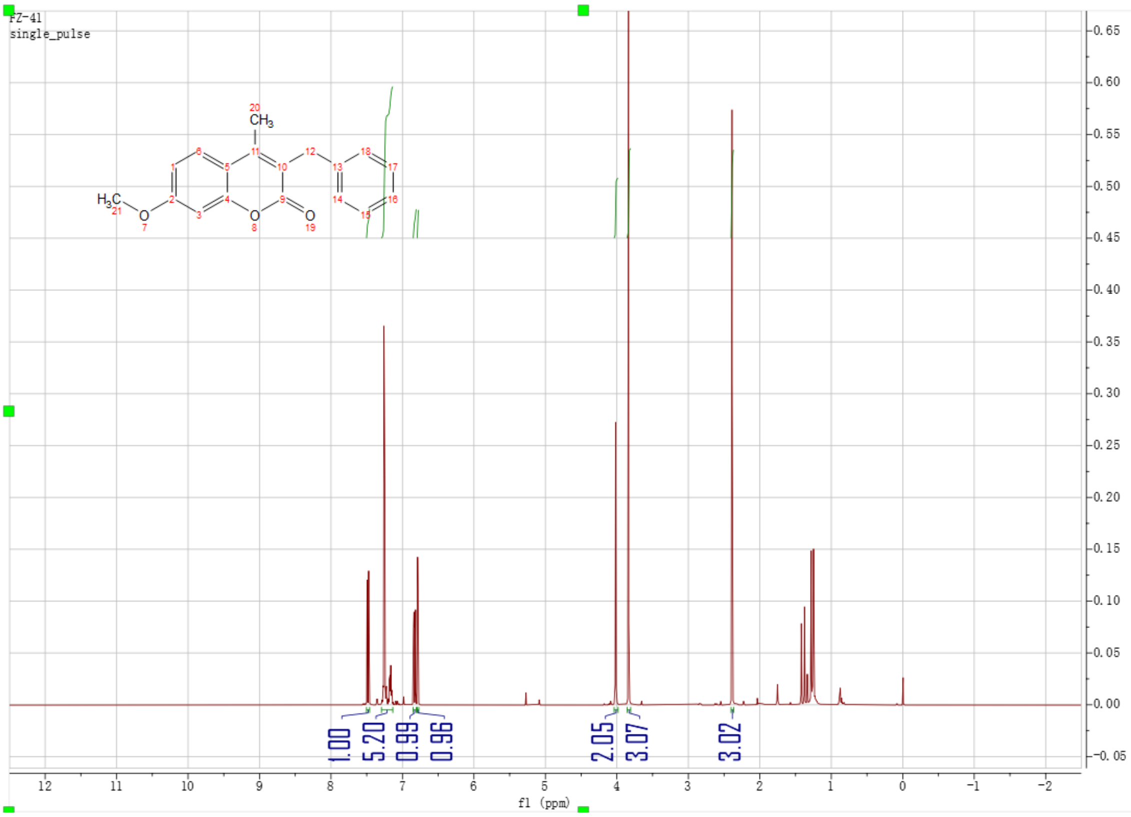

Figure S61. ^1H NMR of compound **22**.

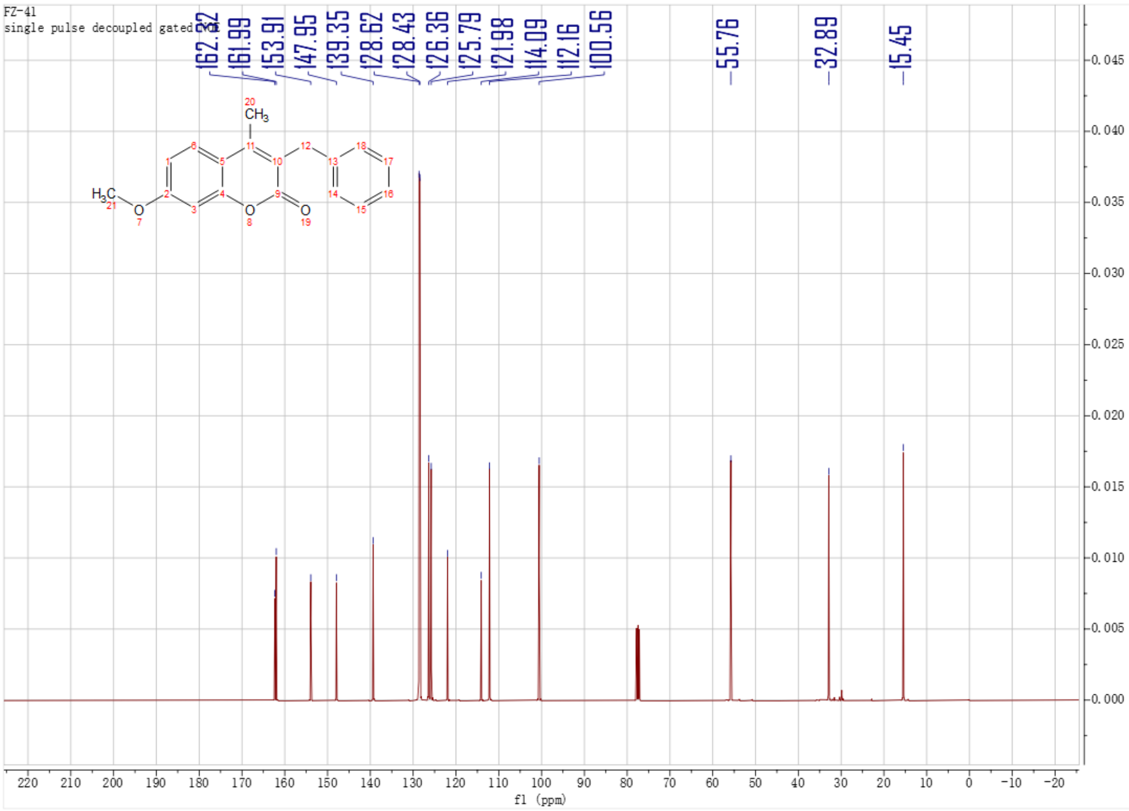

Figure S62. ^{13}C NMR of compound **22**.

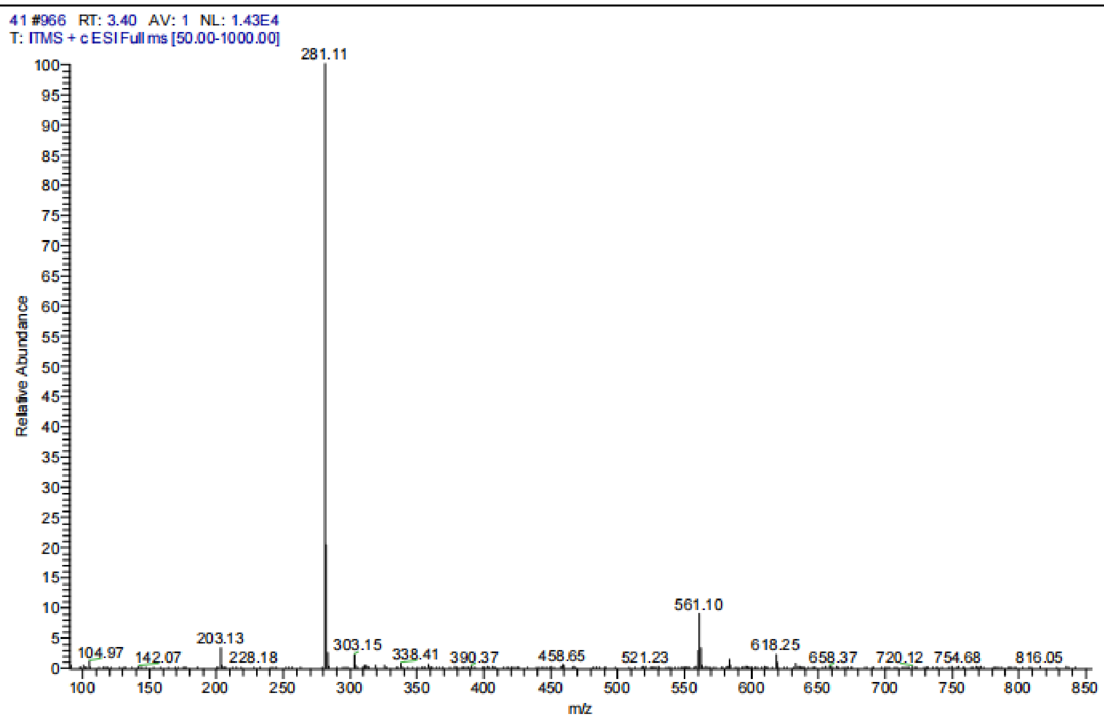

Figure S63. ESI-MS of compound **22**.

23. 4-Benzyl-7-ethoxy-4-methyl-2H-chromen-2-one (compound 23).

White amorphous powder, yield: 8%. ^{1}H NMR (400 MHz, $CDCl_3$) δ: 7.49 (1H, d, *J* = 8.8 Hz), 7.28-7.22 (5H, m), 6.83 (1H, dd, *J* = 8.8, 2.5 Hz), 6.79 (1H, d, *J* = 2.5 Hz), 4.07 (2H, q, *J* = 2.5 Hz), 4.02 (2H, s), 2.40 (3H, s), 1.44 (3H, t, *J* = 7.0 Hz). ^{13}C NMR (100 MHz, CD_3Cl) δ: 162.5, 161.4, 154.0, 148.0, 139.3, 128.6 (2C), 128.4 (2C), 126.3, 125.7, 122.0, 114.0, 112.7, 101.1, 64.2, 32.9, 15.5, 14.7. ESI-MS: *m*/*z* 295 [M+H] $^{+}$; calcd for $C_{19}H_{18}O_3$, 294.35.

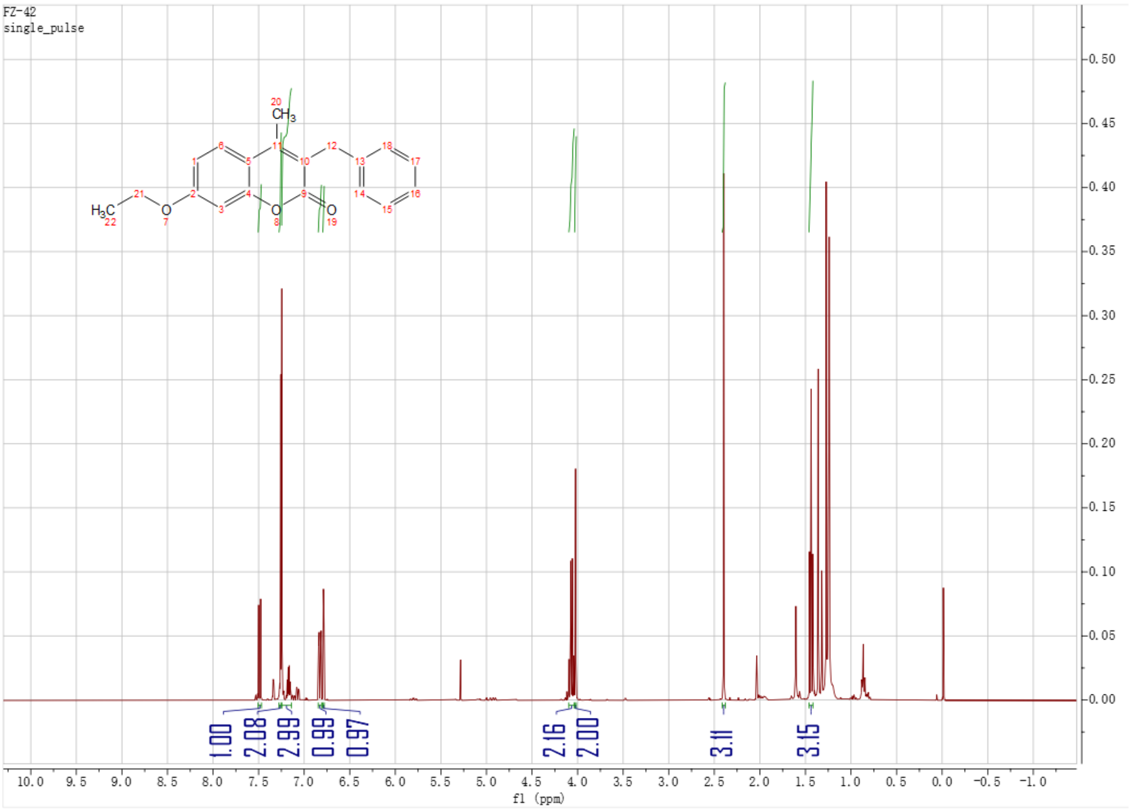

Figure S64. ^{1}H NMR of compound **23**.

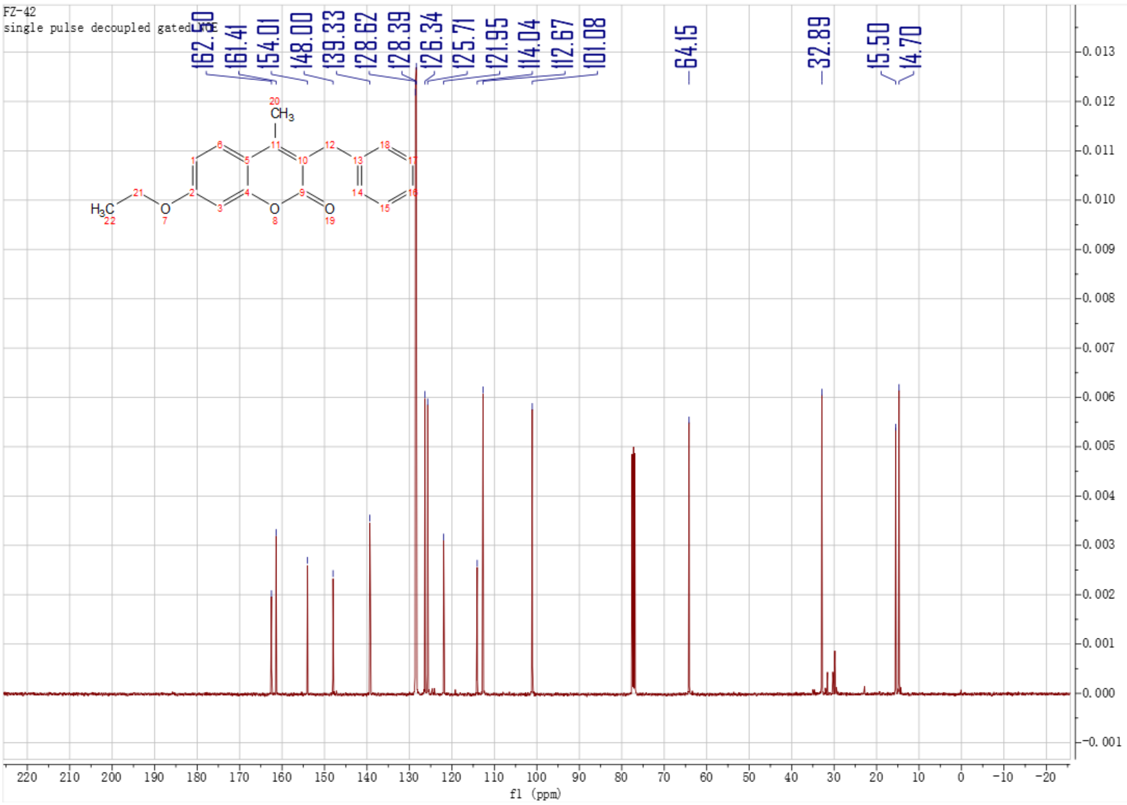

Figure S65. ^{13}C NMR of compound **23**.

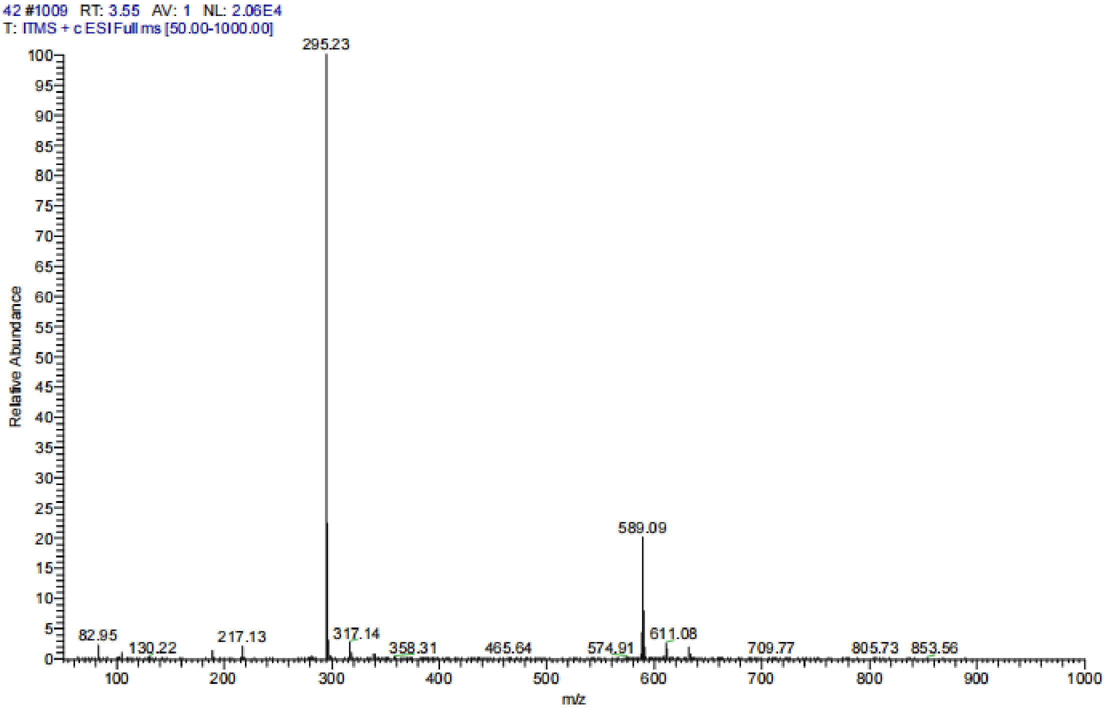

Figure S66. ESI-MS of compound **23**.

24. 3-Benzyl-4-methyl-2-oxo-2H-chromen-7-yl acetate (compound 24).

White amorphous powder, yield: 11%. ^{1}H NMR (400 MHz, $CDCl_3$) δ: 7.61 (1H, d, *J* = 8.7 Hz), 7.28-7.15 (m, 5H), 7.09 (1H, d, *J* = 2.3 Hz), 7.05 (1H, dd, *J* = 8.7, 2.3 Hz), 4.05 (s, 2H),

2.43 (s, 3H), 2.33 (s, 3H). ^{13}C NMR (100 MHz, $CDCl_3$) δ: 169.0, 161.8, 153.0, 152.4, 147.2, 138.8, 128.7 (2C), 128.3 (2C), 126.5, 125.6, 124.9, 118.6, 118.2, 110.3, 33.0, 21.2, 15.6. ESI-MS: *m*/*z* 309 [M+H] +; calcd for $C_{19}H_{16}O_4$, 308.33.

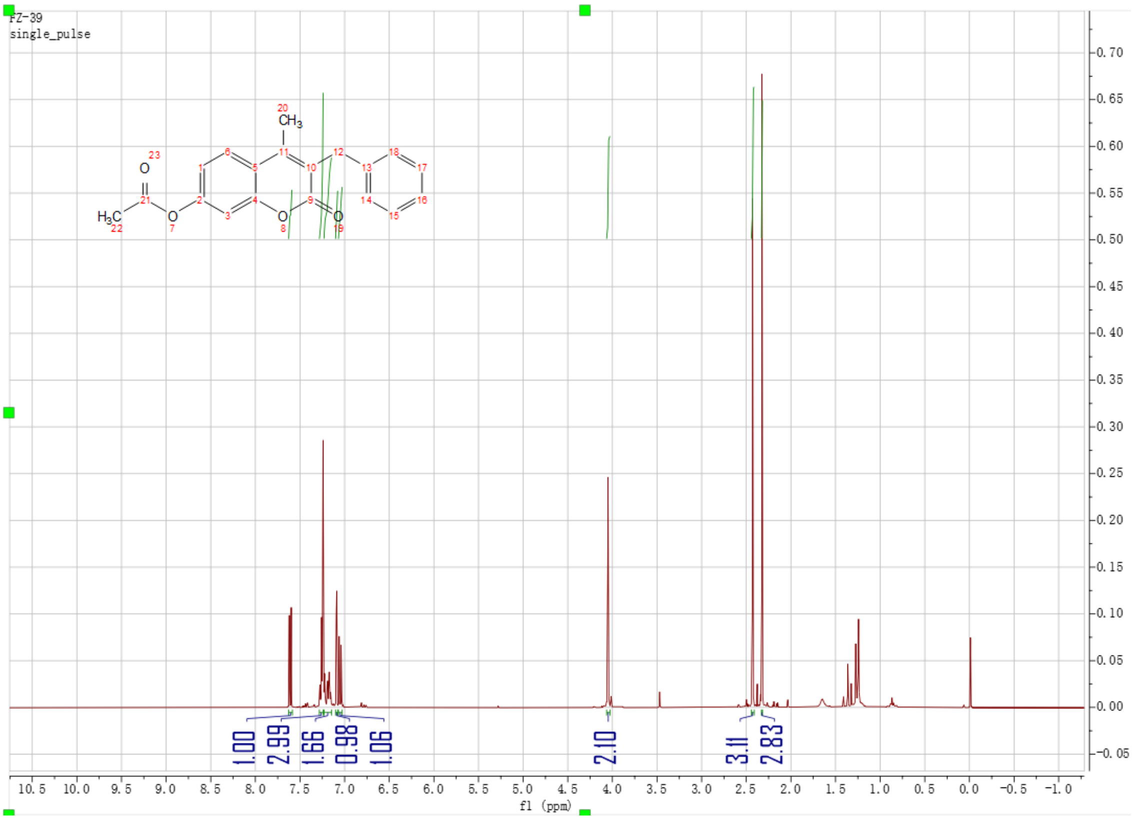

Figure S67. ^1H NMR of compound **24**.

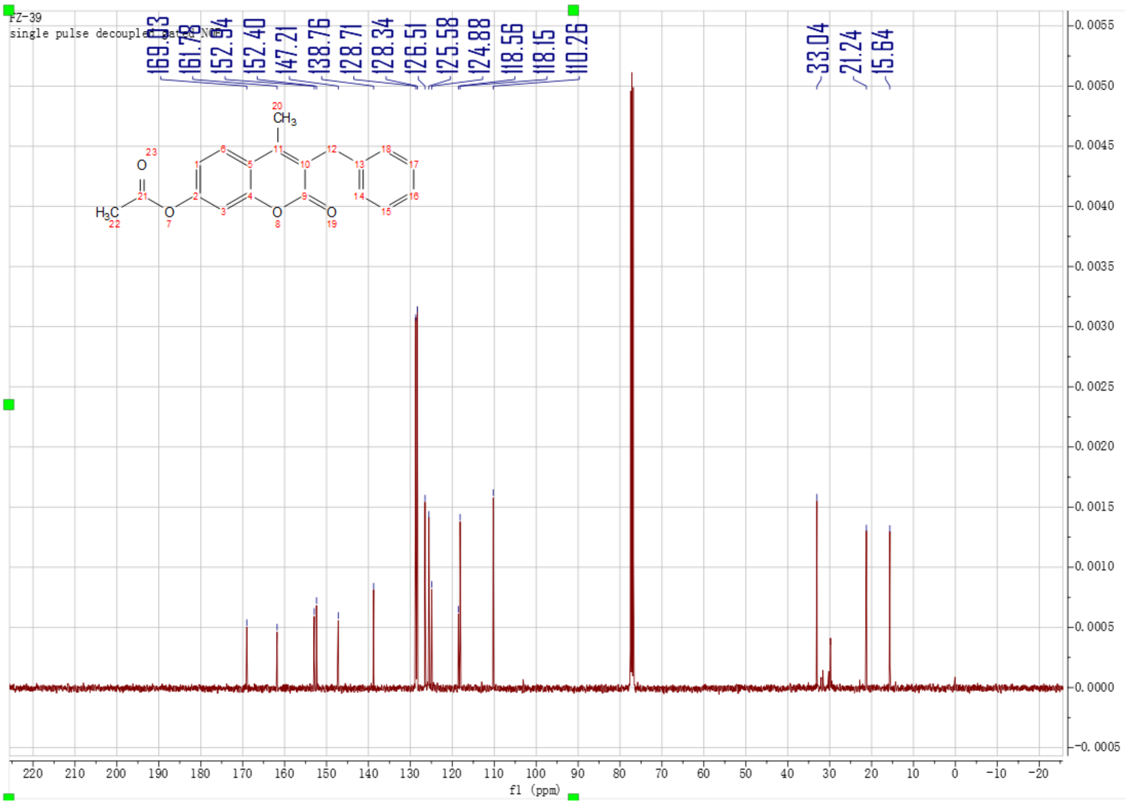

Figure S68. ^{13}C NMR of compound **24**.

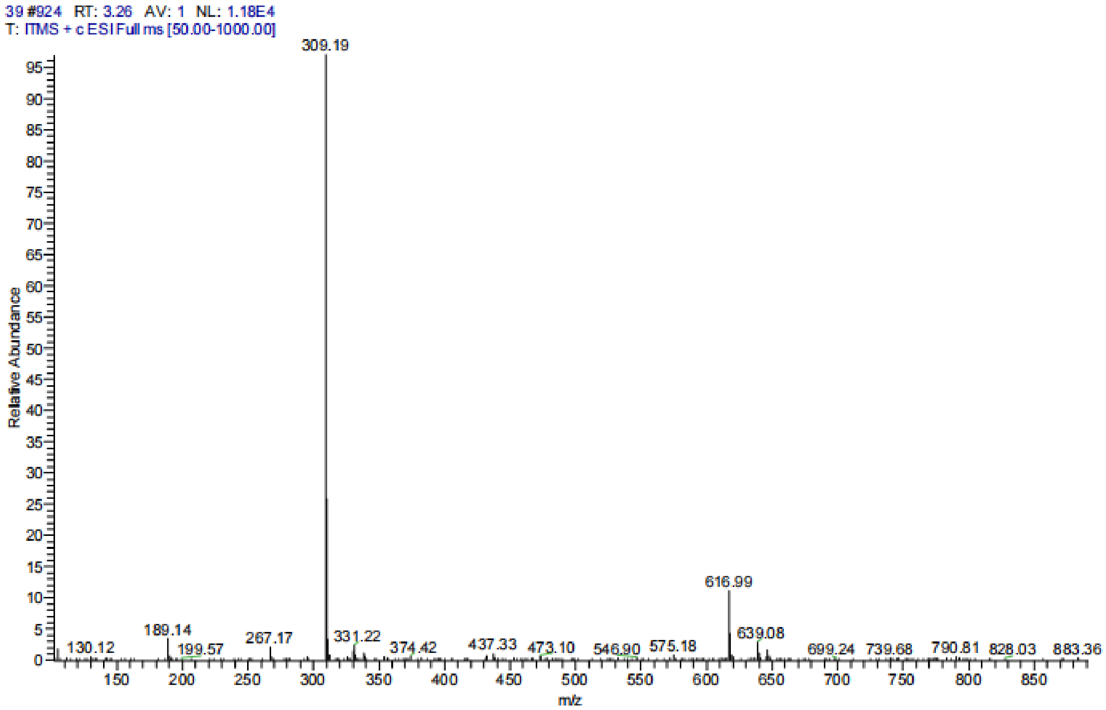

Figure S69. ESI-MS of compound **24**.

25. 3-Benzyl-4-methyl-2-oxo-2H-chromen-7-yl propionate (compound 25).

White amorphous powder, yield: 6%. ^{1}H NMR (400 MHz, CD_3OD) δ: 7.61 (1H, d, *J* = 8.7 Hz), 7.30-7.13 (5H, m), 7.09 (1H, d, *J* = 2.3 Hz), 7.04 (1H, dd, *J* = 8.7, 2.3 Hz), 2.62 (2H, q, *J* = 7.5 Hz), 2.43 (3H, s) 1.27 (3H, t, *J* = 7.5 Hz). ^{13}C NMR (100 MHz, $CDCl_3$) δ: 172.5, 161.8, 152.9, 152.6, 147.3, 138.8, 128.7 (2C), 128.4 (2C), 126.5, 125.6, 124.8, 118.4, 118.2, 110.2, 33.0, 27.8, 15.6, 9.0. ESI-MS: *m/z* 323 [M+H] [+]; calcd for $C_{20}H_{18}O_4$, 322.36.

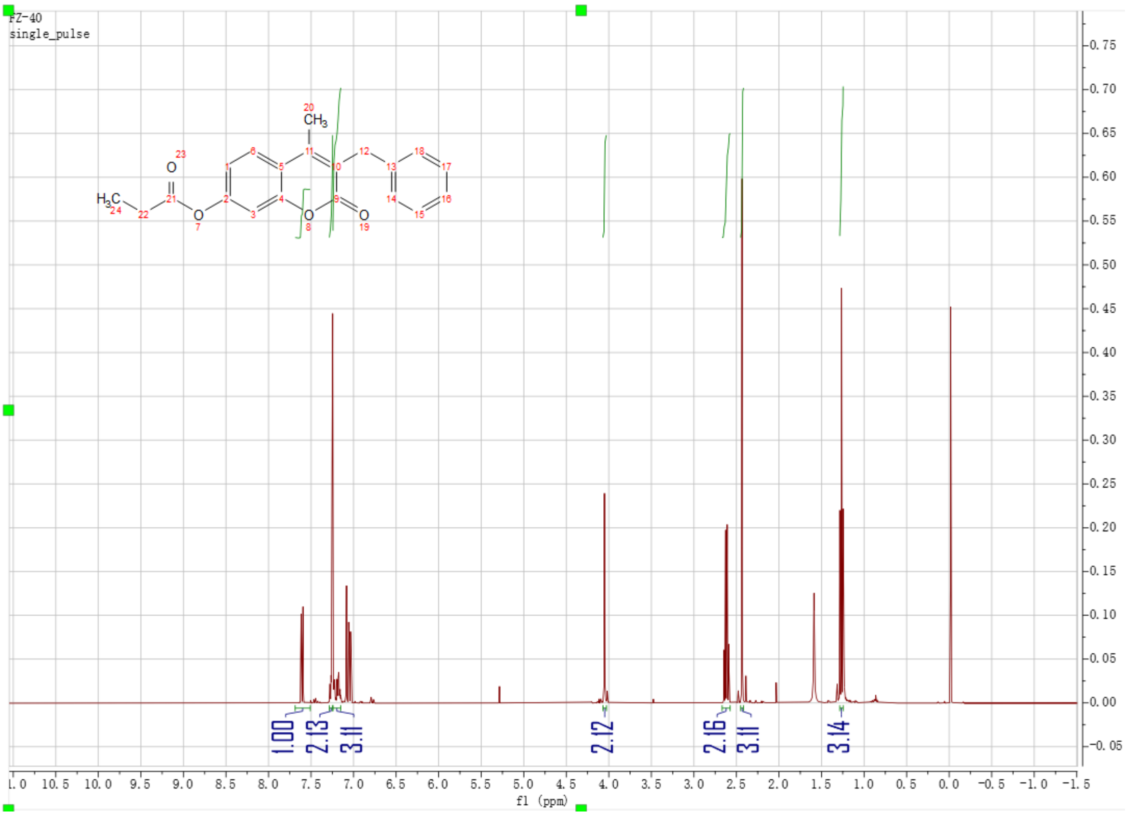

Figure S70. ^{1}H NMR of compound **25**.

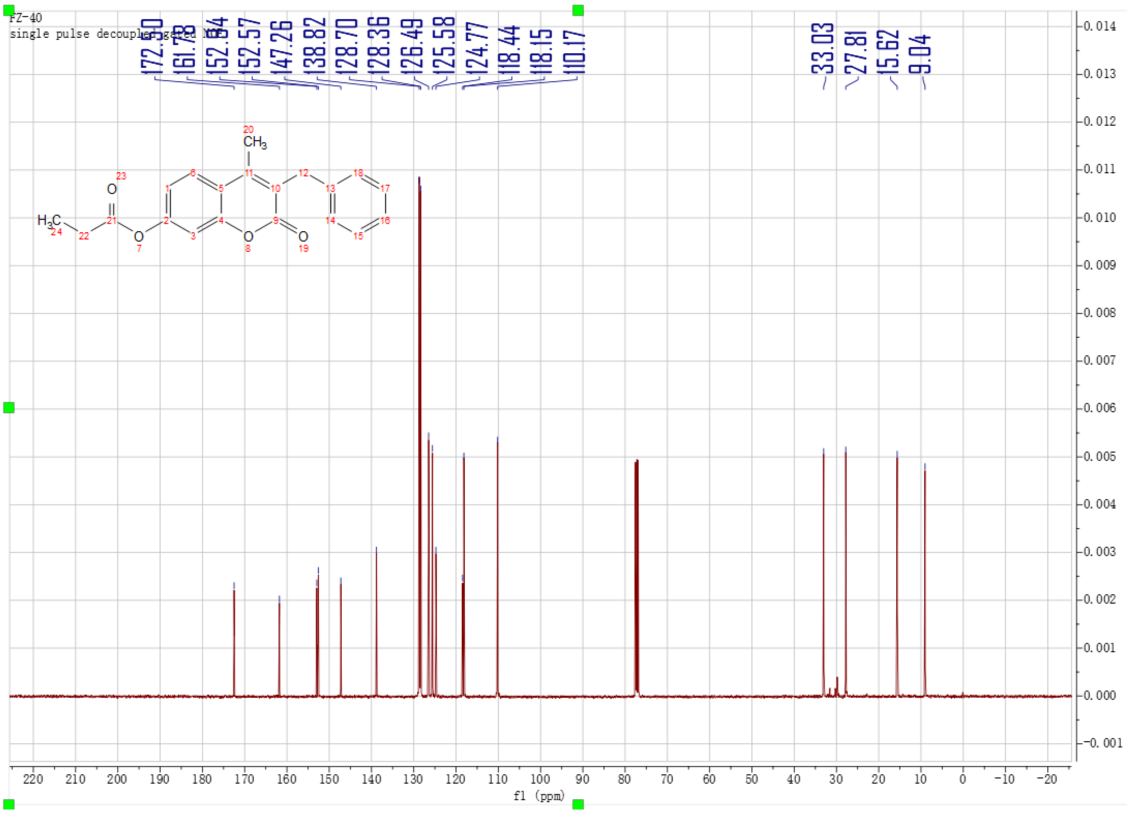

Figure S71. ^{13}C NMR of compound **25**.

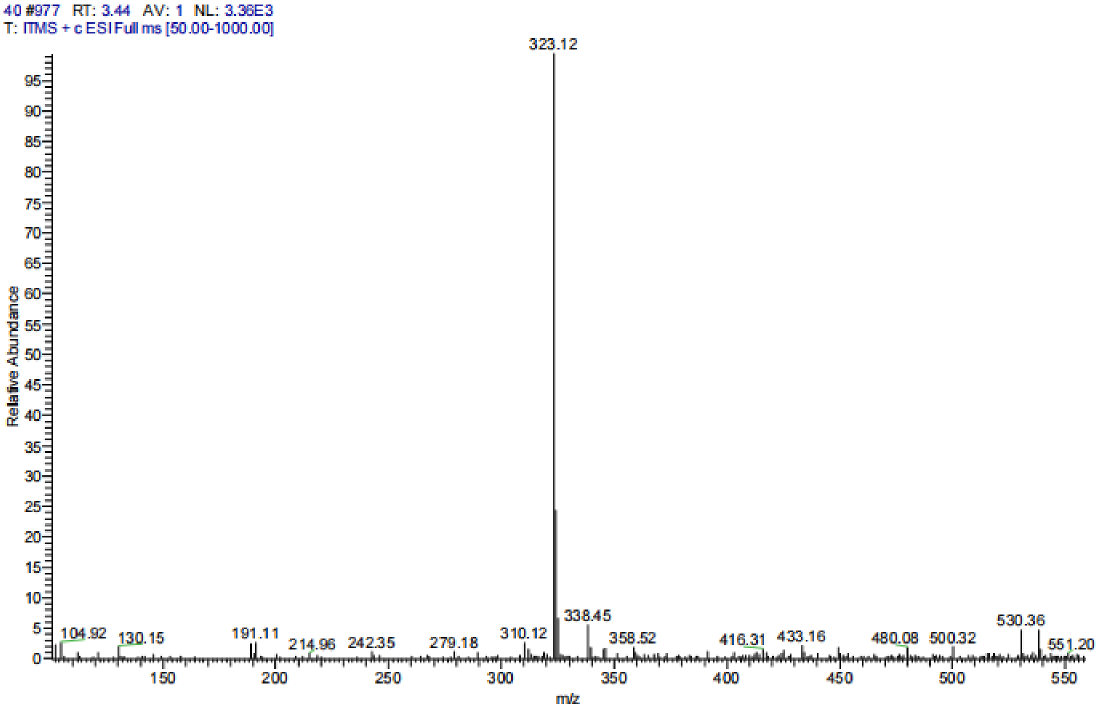

Figure S72. ESI-MS of compound **25**.

26. Biological Evaluation of The Synthesized Coumarin Derivatives at GPCRs in vitro

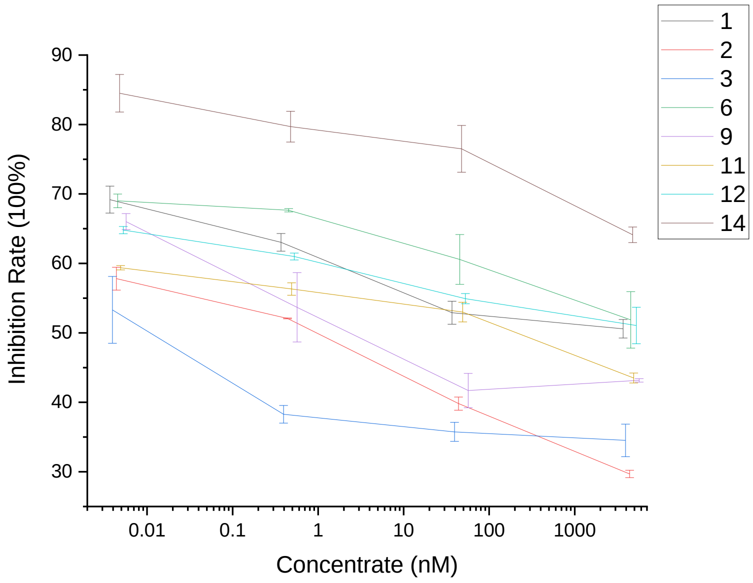

Figure S73. The relationship between concentrate and inhibition rate of compounds **1–3**/ **6**/ **9**/ **11–12**/ **14**.

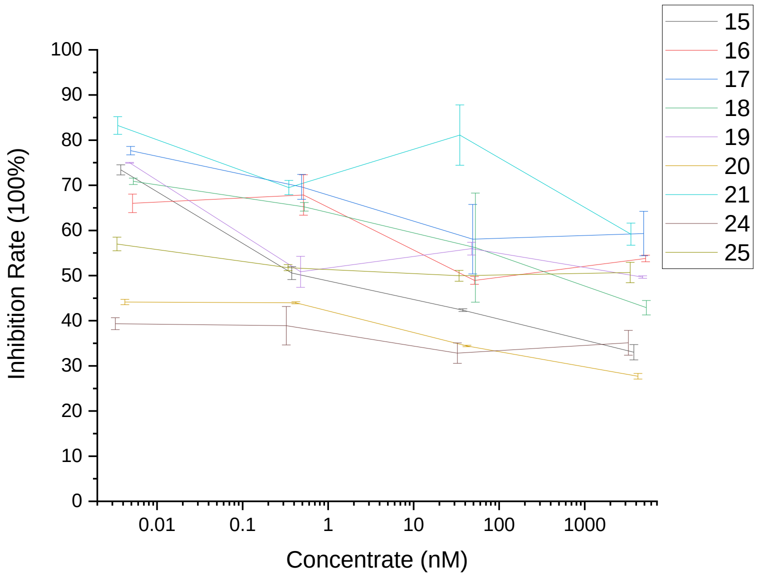

Figure S74. The relationship between concentrate and inhibition rate of compounds **15–21** and **24–25**.

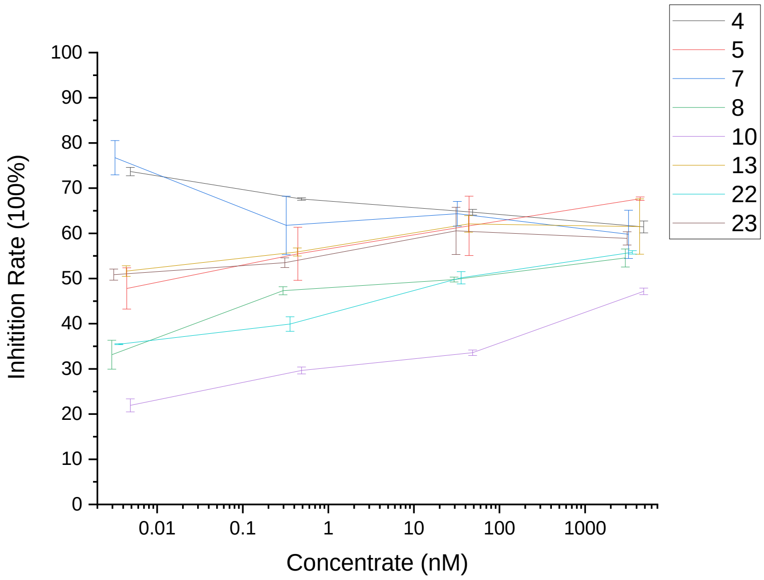

Figure S75. The relationship between concentrate and inhibition rate of compounds **4**–**5**/ **7**–**8**/ **10**/ **13** and **22**–**23**.
